# Self-explaining artificial intelligence for the classification of B cell non-Hodgkin lymphoma: A diagnostic decision support study

**DOI:** 10.1371/journal.pmed.1004889

**Published:** 2026-07-13

**Authors:** Michael C. Thrun, Jörg Hoffmann, Stefan W. Krause, Peter Krawitz, Quirin Stier, Andreas Neubauer, Cornelia Brendel, Alfred Ultsch

**Affiliations:** 1 Mathematics and Computer Science, Philipps University Marburg, Marburg, Germany; 2 IAP-GmbH Intelligent Analytics Projects, Adelheidsdorf, Germany; 3 Department of Hematology, Oncology and Immunology, Philipps University Marburg, University Hospital Giessen and Marburg, Marburg, Germany; 4 Department of Medicine 5, Universitätsklinikum Erlangen, Erlangen, Germany; 5 Institute for Genomic Statistics and Bioinformatics, University Bonn, Bonn, Germany; University of Augsburg: Universitat Augsburg, GERMANY

## Abstract

**Background:**

Multiparameter flow cytometry is a cornerstone of B cell non-Hodgkin lymphoma (B-NHL) diagnostics, but interpretation requires substantial expertise and is complicated by high-dimensional data, variable sample quality, limited data for rare entities, and evolving clinical classification systems. Current artificial intelligence approaches often require large training datasets and provide limited insight into the rationale behind individual diagnostic decisions.

**Methods and findings:**

We developed FlowXAI, a self-explaining artificial intelligence system designed to support B-NHL classification while explicitly reporting case-level diagnostic trustworthiness. FlowXAI combines unsupervised structural analysis with a clinically motivated, multi-level diagnostic framework reflecting routine diagnostic priorities. An unsupervised Tile Mining (TM) procedure performs pre-diagnostic sample-quality assessment by identifying structurally atypical samples. TM is applied to filter training data, enabling substantial reduction of training requirements while preserving unbiased evaluation on independent test samples.

FlowXAI was evaluated using repeated cross-validation on 19,493 peripheral blood samples and further assessed on an independent external benchmark dataset generated at a separate diagnostic center using a different antibody panel. Across diagnostic levels, FlowXAI achieved performance comparable to a deep learning–based system despite requiring approximately two orders of magnitude fewer training samples. When predictions were classified as confident by the system’s internal self-assessment, diagnostic performance exceeded that of the neural network baseline. Unsupervised structural analysis demonstrated clear separation between normal controls and selected lymphoma entities such as chronic lymphocytic leukemia–like lymphomas and hairy cell leukemia, while other entities were not clearly separable using the antibody panels studied.

**Conclusions:**

FlowXAI provides accurate, data-efficient, and transparent support for B-NHL immunophenotyping from nonstandardized flow cytometry data. By combining interpretable decision logic with explicit self-assessment, FlowXAI offers a clinically meaningful framework for diagnostic support and training, particularly in settings with limited expert availability or rare lymphoma subtypes. The main limitation is the retrospective evaluation using specific antibody panels, and FlowXAI requires prospective validation as a decision-support tool within integrated diagnostic workflows.

## Author summary

**Why was this study done?:**

- Diagnosing B cell non-Hodgkin lymphoma, a blood cancer disease, requires expert interpretation of flow cytometry, a laboratory technique that characterizes blood cell populations by measuring antibody-marker expression patterns and light-scatter properties.
- Interpretation of flow cytometry depends strongly on experimental knowledge of physicians and can be difficult when sample quality is impaired or lymphoma types are rare, and different diagnostic centers use different antibody panels. Therefore, there is a clinical need for transparent decision-support systems that are data-efficient and aligned with real-world diagnostic practice.
- Existing artificial intelligence approaches often require very large training datasets, provide limited insight into individual diagnostic decisions, and lack case-level self-assessment of prediction reliability.

**What did the researchers do and find?:**

- We developed FlowXAI, a self-explaining artificial intelligence system that supports lymphoma classification from flow cytometry data and self-assesses each diagnostic prediction as confident, probable, or challenging.
- FlowXAI was evaluated on 19,493 peripheral blood samples using repeated cross-validation and was further tested on an independent external dataset from a different diagnostic center using a different antibody panel.
- FlowXAI achieves diagnostic performance comparable to a deep learning-based approach, reaches performance levels previously reported for human experts, while requiring approximately 100-fold fewer training samples, and its case-level self-assessment helped identify predictions that were more likely to require expert review.

**What do these findings mean?:**

- Reducing training data requirements is a prerequisite for real-world use of artificial intelligence in flow cytometry-based lymphoma diagnosis, especially for rare lymphoma types for which large training datasets are difficult to obtain.
- FlowXAI may support clinical training, quality assurance, and expert decision-making by combining diagnostic classification with visual explanations and case-level self-assessment that helps identify cases more likely to require closer expert review.
- The main limitation is that the study employs retrospective datasets of specific antibody panels requiring further prospective validation; FlowXAI does not replace expert judgment or additional diagnostic tests such as histopathology and molecular diagnostics.

## Abstract 3

In this diagnostic decision support study, Michael C. Thrun and colleagues develop and validate Self-explaining artificial intelligence system (FlowXAI) for the classification of B-cell non-Hodgkin lymphoma.

## Introduction

Artificial intelligence (AI) has delivered tremendous changes and opportunities to the field of clinical diagnostics; particularly, neural networks (NN) have enabled a new era of image analysis techniques. Multiparameter flow cytometry (MFC) has been used for digital surface protein analysis of cell populations from peripheral blood for about four decades. Moreover, novel high-parametric technologies such as mass cytometry and spectral flow cytometry provide numerous simultaneous measurements of variables. Thus, algorithms that facilitate the selection of relevant cell populations in machine learning–assisted analyses have been proposed [1,2]. Several AI algorithms were developed for automated diagnosis of leukemia and lymphoma [3–5], and an NN-based approach for automated immunophenotyping of mature B cell non-Hodgkin lymphoma (B-NHL) has recently been suggested [6].

Due to the limited availability of well-trained expert physicians, automation of immunophenotyping is highly desirable to support and educate hematologists in the interpretation of antigen expression patterns of different cell populations. However, several obstacles complicate this task. First, lymphoma classification has historically been fluid, and therefore assessing whether the clinical labels used in routine diagnostics are adequately represented in a given dataset is essential. Exact labels of disease entities are important for patient care and follow internationally accepted classifications involving morphology, immunophenotype and genetics. They evolve over time as biological knowledge expands and inevitably incorporate expert judgment. Accordingly, recommendations for the optimal diagnostic panel of markers to dissect different B cell lymphomas are still under investigation [7–9]. In clinical routine, MFC represents an essential and, in selected entities such as chronic lymphocytic leukemia (CLL) and hairy cell leukemia (HCL), often sufficient diagnostic modality [10,11]. However, for the majority of B cell lymphomas, definitive diagnosis requires integration of histopathological findings from lymph node or tissue biopsies, which remain the diagnostic gold standard [12]. Importantly, according to current standards, some lymphoma entities cannot be reliably diagnosed by flow cytometry alone, irrespective of the specific antibody panel used [13]. However, an assignment of B cell lymphoma without subtyping is frequently possible and a "best guess" for assignment of a subtype can be attempted.

Rare lymphoma entities and the generally limited availability of representative samples further impair the performance of NN-based algorithms, thereby restricting their use mainly to university hospitals and high-throughput diagnostic centers. Transfer learning has been proposed for automated B-NHL immunophenotyping to address the requirement for large datasets, and it has been claimed that such AI systems achieve performance comparable to that of human experts. However, performance remained strongly dependent on the amount of training data, and rare lymphoma entities were excluded from evaluation [14,15].

Beyond performance and data availability, self-explanation is a critical aspect of diagnostic AI. Many AI systems, particularly those based on NN, are subsymbolic [16–18]. While subsymbolic systems may assign a diagnosis to a sample, they are typically unable to provide human-understandable reasons or explanations for their decisions [19,20], despite being highly effective learners [21]. Consequently, concerns regarding trustworthiness and patient safety have become subjects of intense debate [22], and European Union regulations increasingly require causal justification for decisions made by AI systems [23,24]. Moreover, transparency of machine learning algorithms is one of the major aspects of the harmonized TRIPOD+AI consensus guidelines which define the major prerequisites for reporting prediction and diagnosis models [25,26].

Finally, biological variability implies that individual samples may deviate from the typical immunophenotype associated with a given clinical label. To address this challenge, Matutes and colleagues proposed a scoring system for CLL [27,28], reflecting how human experts routinely qualify diagnostic decisions instead of relying on binary classifications. In light of these considerations, we designed a self-explaining AI system, termed FlowXAI, to explicitly address the following key elements:

- Integrating medical knowledge into the classification framework after assessing the structural embodiment of clinically assigned labels in the data.
- Achieving robust diagnostic performance even when only very few samples are available for learning.
- Providing self-explanation by reporting a case-level reliability estimate and delivering human-understandable explanations for each diagnostic decision.

The novelty of FlowXAI lies in the combination of interpretable committee-based decision support, native trustworthiness stratification, and reduced training requirements, rather than in a claim of universal superiority over all alternative classifiers. We therefore asked whether FlowXAI can provide accurate diagnostic classification from flow-cytometry data while reducing training-data requirements and providing interpretable case-level outputs for expert review.

## Methods

### Characterization of the data sets

The MLL9 and PUM2 datasets are entirely independent, both medically and technically. They originate from two separate diagnostic laboratories and were generated using different antibody panels. The MLL9 dataset contains 19,493 peripheral blood samples, which were selected from a larger cohort for this work [6]. The panel of the Munich Leukemia Laboratory (MLL) consists of three 9-color tubes with the following antibodies:

1. CD19(APCA750) + CD45(KrOr) + FMC7(FITC) + CD10(PE) + IgM(ECD) + CD79b(PC5.5) + CD20(PC7) + CD23(APC) + CD5(PacBlue);
2. CD19(APCA750) + CD45(KrOr) + kappa(FITC) + lambda(PE) + CD38(ECD) + CD25(PC5.5) + CD11c(PC7) + CD103(APC) + CD22(Pac Blue);
3. CD19(APCA750) + CD45(KrOr) + CD8(FITC) + CD4(PE) + CD3(ECD) + CD56(APC) + HLA-DR(PacBlue).

The PUM2 dataset was collected unicentrically in Marburg [7,29] and employs two tubes:

1. CD45(KrOr) + kappa/CD8(FITC) + lambda/CD7(PE) + CD23(ECD) + CD79b/CD4(PC5.5) + CD5(PC7) + CD38(APC) + CD19(AP-A700) + CD20/CD3(APC-A750) + FMC7/CD2(PacBlue);
2. CD19(KrOr) + CD103(FITC) + CD43(PE) + CD25(ECD) + CD10(PC5.5) + CD200(PC7) + CD11c(AP-A700) + CD20(APC-A750) + IgM(Pac Blue).

Flow cytometer measurements consist of *N* = 50,000 (MLL9) and *N* = 100,000 (PUM2) events per tube and sample.

### Overview of FlowXAI

FlowXAI was developed as a decision-support system for B-NHL classification based on multiparameter flow cytometry data. Its workflow comprises three linked stages: First, Tile Mining (TM) which screens samples for structural atypicality, second, a hierarchical committee of interpretable diagnostic experts, and, third, case-level self-assessment, which reports the trustworthiness of each predicted diagnosis.

### Training data reduction by Tile Mining

In stage one, TM is an unsupervised, label-independent step that assesses sample-level structural atypicality before supervised learning. It is used to flag atypical files for review and to prevent structurally atypical samples from disproportionately influencing representative-case selection and model training. After compensation and signed-log transformation, each sample was represented by the concatenated nine-tile summaries of all unique marker pairs as follows. For each sample and tube, TM considers all unique bivariate combinations of the d measured markers, yielding


(d2)=d(d−1)2

marker pairs. For each marker pair, the observed value range is determined per case, and the corresponding bivariate space is partitioned into a fixed grid of nine rectangular tiles.

For a marker set of size d, let $H_{i}(d)$ denote the concatenated tile profile of case i. Across all marker pairs, this procedure yields


$$\begin{array}{c} \#H_{i}(d)=\text{9}*\frac{d(d-\text{1})}{\text{2}}. \end{array}$$

tiles per sample. For d = 11 markers, this corresponds to #H(d=11)=495 tiles. For each tile h∈H(d), the density p(h) is defined as the proportion of events falling into that tile:


$$\begin{array}{c} p(h)=\frac{1}{\left|E\right|}\sum_{e\;\in E\;}f_{e},{\;f}_{e}=\left\{ \begin{array}{cc} \text{1} & e\;\epsilon \;h\\ \text{0} & e\notin h \end{array}\right. \end{array}$$

where E denotes the set of all measured events in the sample. For each variable pair, the resulting matrix of proportions is normalized by tile area and rescaled to percentages ensuring comparability across tiles within each sample.

Let $\bar{T}$ denote the robust reference tile profile estimated from the cohort. TM centers each $H_{i}(d)$ in relation to $\bar{T}$ by applying robust feature-wise standardization, yielding values $p_{ij}^{*}$. For a given sample i and tube t, the normalized tile values are aggregated to yield a strangeness value:


$$\begin{array}{c} \sigma_{i,t}=\frac{\text{100}}{\#H_{i}(d)}\sum_{j=\text{1}}^{\#H_{i}(d)}p_{ij}^{*}. \end{array}$$

Hence, $\sigma_{i,t}$ summarizes the average robustly standardized deviation of the case from the reference tile profile. Between-sample comparison is therefore performed in a common normalized tile-feature space rather than on identical absolute raw-value borders.

Empirically, the distribution of $\sigma_{i,t}$ across samples exhibits an approximately normal shape under typical conditions. We provide visualizations of the empirical strangeness distributions for the study cohorts and discuss where the robust Gaussian approximation is adequate and where departures from Gaussian shape likely reflect cohort heterogeneity in Text H in S1 Appendix: Strangeness Distributions and Tile Mining Parameters. This observation provides a heuristic justification for modeling strangeness values using an empirical normal distribution ${N_{\sigma }}_{i,t}(m,sd)$ estimated robustly from the data. A robust Gaussian reference model was fitted to the empirical strangeness distribution and used as a pragmatic decision rule for structural atypicality. We therefore use the normal model as an empirical thresholding device rather than as an assertion of exact Gaussianity in every dataset. Cases were classified as atypical when their strangeness values fell outside the two-sided limits implied by the chosen central confidence region. For a chosen central confidence level 1−α, a case was classified as atypical when $\sigma_{i,t}<m-k*sd$ or $\sigma_{i,t}>m+k*sd$, where m and sd denote the robustly estimated location and scale of the strangeness distribution, respectively, and k is the corresponding two-sided critical factor.

Any sample identified as atypical in at least one tube is excluded from subsequent learning steps but may still be retained for downstream evaluation. In this way, TM ensures that only structurally typical and internally consistent samples contribute to the training of supervised components, while preserving the ability to analyze atypical cases separately.

### Self-explanatory diagnostic AI committee

**Fig 1:**
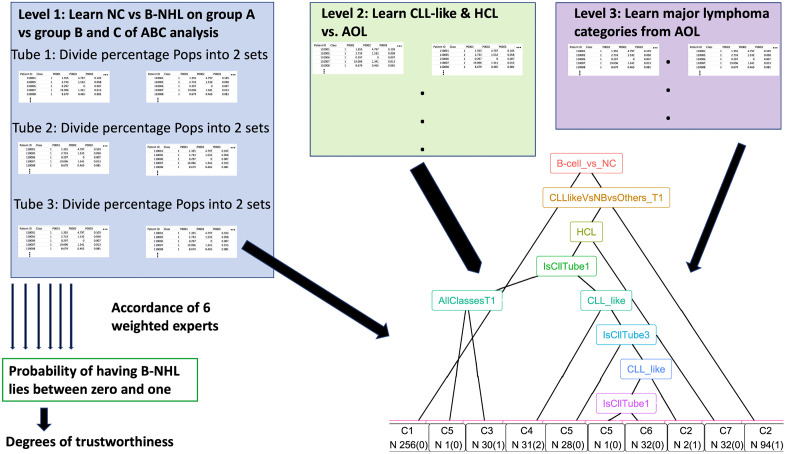
Flow chart for the ALPODS committee assembly. Flow chart illustrating the assembly of the ALPODS expert committee within FlowXAI. Tile Mining (TM) identifies structurally typical samples, from which representative cases are selected for supervised learning. ALPODS experts are trained separately per diagnostic level and tube, reflecting clinically defined decision tasks. Expert opinions are combined within a committee structure, and final diagnostic decisions are produced by a standard decision tree operating on expert-level outputs. ALPODS yields cell populations which are used within the committee of experts to judge the degree of trustworthiness for self-assessment and to yield of the final diagnosis. For each case consisting of one or more tube/sample files, ALPODS-derived cell populations provide the basis for tube- and diagnostic-level–specific expert decisions. The committee integrates these expert outputs to learn the final case-level classification and to assign a self-assessed degree of trustworthiness, indicating whether the diagnosis is confident, probable, or challenging.

The second stage of FlowXAI consists of a committee of AI experts that performs self-explanatory diagnosis in a process analogous to human expert reasoning. This supervised component consists of tube- and task-specific ALPODS experts, an extension of the original ALPODS algorithm [30]. In this context, an ‘expert’ denotes a data-derived rule-based decision module. Each expert is trained for a specific tube and diagnostic task. During training, ALPODS identifies diagnostically informative cell subpopulations, derives rules for these populations, and returns a task-specific output. The relevant cell populations are defined by rules learned from a representative sample of the training data. The rules are composed of conditions which recursively partition the data set on the most informative features at each level. The resulting decision support logic can be expressed as explicit marker-threshold logic. To make the decision support logic inspectable, we provide exported PUM2 decision logic as supplementary digital material S1 Data. The provided rule files represent the tube 1 and tube 2 experts. Each rule file assigns samples to rule-defined populations and diagnostic outputs through explicit marker conditions. The accompanying subject-level population-frequency files link the rule logic to case-level explanatory outputs. Full algorithmic details are provided in Fig 1, the Text A and Fig A in S1 Appendix.

We have created a live, interactive demonstration of our FlowXAI platform at https://plait.mathematik.uni-marburg.de/ which is intended as an optional interactive demonstration. This online portal is specifically designed so that physicians, computational scientists, and readers can explore our proposed step-by-step classification workflow: visitors can upload data (or select from example datasets) and see how FlowXAI automatically identifies relevant cell populations, performs classification, and provides self-explaining visual outputs. Each FlowXAI decision is grounded in traceable rules that mimic the logic of human experts. FlowXAI provides these understandable explanations stored in the FCS format. We provide inspectable case-level explanatory outputs (S1 Data), an interactive portal, and exemplary decision logic that allow readers to trace diagnostically relevant populations and decision-support output.

### Self-assessment by case-level trustworthiness degrees

In the third stage, FlowXAI performs a case-level self-assessment to estimate the trustworthiness of its diagnosis. The ALPODS committee produces a reliability estimate for self-competence. This estimate reflects the internal consistency and agreement of expert decisions across diagnostic levels. The resulting score is therefore used in an ordinal manner only.  To support intuitive interpretation in clinical contexts, the internal reliability score is mapped onto a three-point trustworthiness scale inspired by Likert-type assessments [31]. A diagnosis is labeled *confident* when expert agreement is maximal, *probable* when agreement remains high but not unanimous, and *challenging* when expert decisions exhibit substantial disagreement. This stratification enables FlowXAI to communicate diagnostic certainty transparently while preserving interpretability and alignment with clinical reasoning.

For the prediction of each sample, the reliability estimate $p_{c}$ is binned as follows: The FlowXAI is *confident* about the diagnosis of a sample if either $p_{c}$ =1 or $p_{c}$=0, *probable* in the two bins of $\text{1}>p_{c}\geq \;\text{0}.\text{8}$ or $\text{0}<p_{c}\leq \text{0}.\text{2}$, and *challenging* for the remaining bin of the central zone $\text{0}.\text{8}>p_{c}>\text{0}.\text{2}$.

### Generalization and validation protocol

To evaluate generalizability and robustness, FlowXAI was assessed using repeated cross-validation on two independent external benchmark dataset. All FCS files were processed using the available laboratory compensation information and transformed by signed-log scaling. Because MLL9F and PUM2 were acquired in different laboratories with different antibody panels and tube configurations, the PUM2 analysis was employed as a cross-site benchmark of the FlowXAI framework under heterogeneous conditions. MLL9F was measured using several instruments of the same type. For the MLL9F dataset, 100 repetitions of class-balanced 80/20 train–test splits were performed (*N* = 15,594 training samples and *N* = 3,899 test samples). Unless explicitly stated otherwise for a given experimental condition, TM–identified atypical samples were excluded from the training set. TM was applied as an unsupervised preprocessing step to the full dataset before repeated train/test splitting and was used exclusively for training-sample curation. Recomputing TM separately within each training fold would represent a stricter design and the current TM implementation is acknowledged as a methodological limitation. In contrast to the prior study, which relied on a single-split evaluation. [6], repeated cross-validation was employed to simultaneously estimate expected generalization performance and assess variability across random partitions, thereby reducing the risk of optimistic bias [32,33].

To quantify the effect of training data reduction, an additional experiment was conducted in which only 512 representative training samples (256 normal controls and 256 B cell lymphoma samples) were used. For this experiment, 32 samples per lymphoma entity were selected once from the pool of typical samples identified by TM, and the resulting model was evaluated on all remaining samples, with performance reported separately for typical and atypical cases.

Given the pronounced class imbalance across lymphoma entities, diagnostic performance at levels L2 and L3 was primarily quantified using the Matthews correlation coefficient (MCC), which provides a robust measure under imbalanced conditions [34,35]. Accuracy, false positive rate, and false negative rate were additionally reported, particularly for level L1 tasks with balanced class sizes. Performance metrics were visualized using mirrored-density plots (MD-plots) [36], allowing assessment of the probability density functions (pdfs) of the evaluated metrics. MD-plot visualization enables detection of multimodality in the distributions; when internal statistical testing indicated compatibility with a Gaussian distribution, a corresponding reference curve (magenta line) was displayed, allowing the point estimate to be summarized by its average. Non-Gaussian distributions may indicate heterogeneity introduced by random sampling between training and test sets.

To assess robustness under domain shift, FlowXAI was further evaluated using the PUM2 dataset, an independent external benchmark dataset originating from a separate diagnostic center and employing a different antibody panel and tube configuration [7,29]. In this setting, 100 cross-validation trials were conducted using 200 training samples per trial (100 normal controls and 100 B cell lymphoma samples), all drawn from the subset of samples classified as typical by TM. Test sets remained fully independent and performance was reported separately for typical and atypical cases. This benchmark evaluation was designed to assess transferability of the approach rather than strict replication of performance across laboratories.

Across all experiments, diagnostic performance stratified by the self-assessed degrees of trustworthiness (confident, probable, challenging) was analyzed descriptively as a post-hoc evaluation, providing insight into how predictive performance relates to FlowXAI’s internal reliability estimation. This study is reported as per TRIPOD+AI guideline (S1 Checklist and S2 Checklist, based on the TRIPOD+AI checklist by Collins and colleagues, BMJ 2024;385:e078378, https://doi.org/10.1136/bmj-2023-078378. Licensed under CC BY 4.0. The checklist wording was not modified in S1 Checklist; study-specific responses were added in S2 Checklist).

### Exploratory analysis of structural embodiment in lymphoma immunophenotypes

To explore how clinically defined lymphoma entities are reflected in high-dimensional MFC data, we performed an exploratory structural-embodiment analysis independently of the clinical labels. Clinical labels remain the authoritative diagnostic standard for patient care, but they are assigned within evolving WHO classification systems [37] and may show inter-observer and inter-institutional variability. In addition, some clinically relevant distinctions depend on information not contained in the present immunophenotyping panel, such as morphology, cytogenetics, molecular data, or clinical context. We therefore sought to assess how strongly the measured MFC data embody clinically used categories, rather than to validate or challenge the clinical diagnoses themselves. In this context, structural embodiment refers to two complementary aspects: first, whether clinically established knowledge is represented in the measured data, and second, whether the resulting structures in data correspond to expert clinical labels.

For this purpose, we used the Databionic swarm, a swarm-intelligence and self-organization framework for high-dimensional structure analysis [38]. The method combines projection-based self-organization with generalized U-matrix visualization [39,40], yielding a topographic map in which valleys indicate regions of high similarity and hills indicate structural dissimilarity between samples. Because this step is fully unsupervised, any separation between presumed categories emerges from the self-organization of the data rather than from label information.

We chose this framework because the number and geometry of biologically meaningful structures in the immunophenotypic space were not known a priori. In contrast to many commonly used clustering algorithms, which optimize a predefined objective function and often require assumptions about cluster number, compactness, or geometry, the present exploratory approach does not require specification of the number of groups in advance and is well suited to assessing heterogeneous, irregularly shaped structures in high-dimensional biomedical data [41,42]. In conjunction with clusterability diagnostics, such self-organizing approaches are also useful for evaluating whether the data support partitioning into distinct groups at all, rather than forcing a cluster solution where the underlying structure is weak or continuous [43,44]. For the present study, computation was performed using a multicore CPU implementation, because the data volume made single CPU runtimes impractical for the final analysis. A multicore CPU implementation of the Databionic Swarm is available [77].

## Results

### Bioinformatic view on lymphoma immunophenotype classes

The presence or absence of discernible groups or classes within a given disease entity influences both the interpretation of high-parametric expression data and the capabilities of machine learning approaches [42,45,46]. At the same time, the nomenclature and categorization of lymphoma entities have progressively shifted due to increasing knowledge of molecular genetic aberrations and pathogenic pathways [47,48], posing a particular challenge for AI-based diagnostic systems. To obtain a label-independent bioinformatic view of lymphoma immunophenotypes, we applied the structural-embodiment analysis to the previously published MLL9 dataset, comprising normal controls (NC) and the entities CLL, PL, MBL, HCL, MCL, MZL, LPL, and FL.

**Fig 2:**
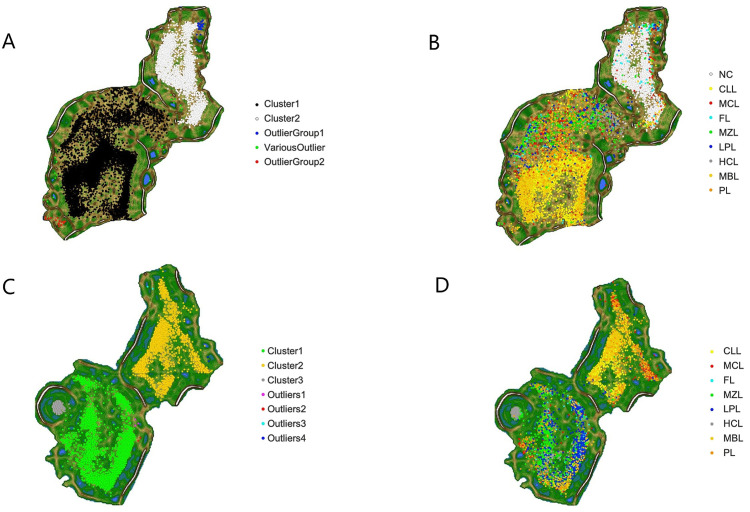
Topographic map of lymphoma based on the Databionic swarm and the generalized U-matrix technique. The topographic map visualizes high-dimensional distances and densities between Databionic-swarm-projected points and allows assessment of the structural-embodiment, with structures in data represented by valleys and mountain ranges. Each point represents a subject sample composed of three tube-specific files from the MLL9F dataset. Coloring indicates clinical labels for lymphoma classes (B,D) or interactive clustering [41,74] (A,C) and was not used during self-organization of the Databionic swarm. Within the topographic map, valleys and basins are depicted as clusters, while the watersheds of hills and mountains serve as cluster boundaries by the following color scheme. The blue colors represent lower elevations (e.g., sea level), green and brown represent intermediate elevations (e.g., low hills), and various shades of white represent higher elevations (e.g., snow-covered mountains). Hypsometric tints use distinct surface colors to represent elevation ranges, with contour lines integrated into the specific color scheme. The high-dimensional data distances and densities of the projected points are mapped to the elevation ranges. The exact mapping is defined in [75]. Additionally, the visual borders in the topographic map are toroidal, i.e., exhibit cyclic connections with periodicity. **A)** The structures in a subset of the MLL9F dataset (3,000 NC, 3,000 CLL-like, and 3,000 non-CLL-like B-NHL samples), visualized by the topographic map, reveal two dominant valleys containing the clusters, two outlier-associated structures, and isolated atypical samples. Isolated atypical samples are shown as individual outliers, whereas the outlier-associated structures are listed as outlier groups in Table A in S1 Appendix, the former as outlier groups in the same table. **B)** The same topographic map highlights a pronounced structural separation between normal controls (NC) and B-NHL samples (see Table A in S1 Appendix for contingency table)**.** Without TM incorporation, many single outliers scatter across the topographic map, illustrated as single dots in single valleys. **C)** After application of Tile Mining (TM) for elimination of structurally atypical samples, the topographic map representing the self-organization of lymphoma cases reveals three dominant valleys that contain the clusters and markedly fewer isolated outliers, (see Table B in S1 Appendix for contingency table.). **D)** Mapping of clinical labels onto the TM-filtered topographic map reveals correspondence between the major structures and CLL-like entities, HCL, and all other lymphomas (AOL), while some entities remain not clearly separable (see Table B in S1 Appendix for contingency table). Figure 2 was newly generated by the authors for this manuscript in R using the packages DatabionicSwarm (https://CRAN.R-project.org/package=DatabionicSwarm) [38,76,77] and GeneralizedUmatrix(https://CRAN.R-project.org/package=GeneralizedUmatrix) [39,41,74,75,78] and does not reproduce or adapt any previously published figure.

The resulting topographic map (Fig 2a) revealed two dominant structures, two outlier-associated structures, and a number of highly atypical individual samples. At the global level, the analysis showed a clear separation between presumed healthy individuals and B cell non-Hodgkin lymphomas (B-NHL) (Fig 2b). Only 3 of 2,998 NC samples were structurally located within the B-NHL-dominated region, whereas a substantial subset of B-NHL samples was embedded within the NC valley (compare cluster with classes in Table A in S1 Appendix). This asymmetric pattern indicates strong global separation between normal and neoplastic samples, while at the same time highlighting marked structural heterogeneity within the lymphoma group.

Within the lymphoma compartment, some entities were structurally more distinct than others. In particular, CLL-like cases formed a comparatively well-separated structure, whereas entities with more subtle or overlapping immunophenotypic features, such as MCL and FL, were less clearly isolated at this global level of analysis.

Subsequently, the TM algorithm was applied to eliminate structurally atypical samples, and a second topographic map was computed based on the remaining lymphoma cases. This analysis revealed three dominant structures and only a small number of remaining isolated or paired outliers (Fig 2c). Mapping clinical labels onto this projection (Fig 2d) showed that the AI-driven immunophenotypic organization of B-NHL does not fully align with WHO-based lymphoma categories. CLL, MBL, and PL formed one coherent structure, while FL, LPL, and MZL were associated with a second structure. HCL appeared as a clearly distinct structure surrounded by pronounced topographic separations, although this structure also contained a limited number of MZL cases (Table B in S1 Appendix). In addition, PL and MCL occupied closely related structural areas, whereas MZL, LPL, and FL, which are often clinically indolent lymphomas, were located more distantly.

Empirically distinct treatment strategies have emerged for entities such as CLL, HCL, and more common lymphocytic lymphomas including MZL, LPL, FL, or MCL. The observed immunophenotypic structures are consistent with these biological similarities and divergences. Notably, even within a well-defined entity such as CLL, prediction of treatment requirement and response remains highly variable and extends beyond B cell morphology to include clinical parameters and T-cell distribution patterns [49]. Whether the AI-derived immunophenotypic organization reflects treatment-related groupings or clinical outcome parameters requires further investigation using additional comprehensive lymphoma datasets.

Taken together, the swarm-based self-organization analysis revealed substantial structural-embodiment of clinically defined lymphoma entities and immunophenotypic structures in data for the most common lymphomas, whereas MZL and LPL were not clearly separable within the limitations of the applied flow cytometry panel.

### Strategy for AI training on B-NHL data

**Fig 3:**
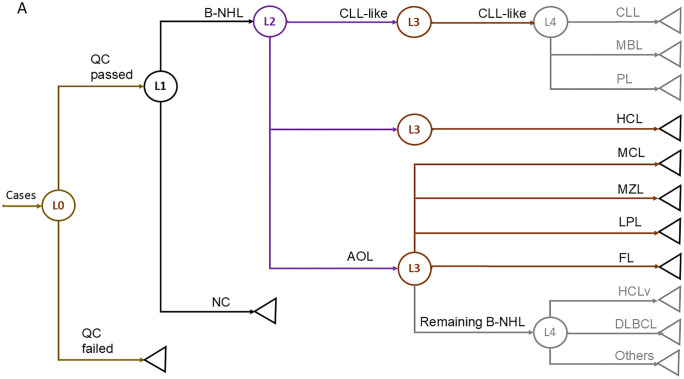
Hierarchical diagnostic decision tree used for FlowXAI. The diagnostic tree summarizes the stepwise decision process across levels L0 to L4, reflecting computed aggregates or diagnoses in alignment with the clinically prioritized diagnostic workflow. L0 (QC) performs sample-quality assessment using Tile Mining (TM). L1 distinguishes normal controls (NC) from B cell non-Hodgkin lymphoma (B-NHL). L2 separates CLL-like entities (CLL, MBL, PL) and hairy cell leukemia (HCL) from all other lymphomas (AOL). L3 differentiates common mature B cell lymphomas (MCL, MZL, LPL, FL). L4 comprises remaining rare entities and large B cell lymphomas and is not considered for further automated subtype classification (gray). Abbreviations: QC, quality control, B-NHL, B cell non-Hodgkin lymphoma; NC, normal control; CLL-like = CLL&MBL&PL; HCL, hairy cell leukemia, AOL = all other malignant lymphoma; CLL, chronic lymphocytic leukemia; MBL, monoclonal B cell lymphocytosis; PL, prolymphocytic leukemia; MCL, mantle cell lymphoma; MZL, marginal zone lymphoma; LPL, lymphoplasmacytic lymphoma; FL, follicular lymphoma; HCLv = hairy cell leukemia variant; DLBCL, diffuse large B cell lymphoma; Others = all remaining rare entities like Burkitt lymphoma (BL).

The observation that AI-derived immunophenotypic organization does not entirely correspond to predefined lymphoma diagnoses indicates potentially compromised learning conditions for a fully automated lymphoma immunophenotyping approach. Guided by the structural-embodiment analysis and by clinical diagnostic priorities, the diagnostic tree was therefore designed as a stepwise, level-based decision framework spanning levels L0 to L4 (Fig 3).

At level L0 (QC), the TM algorithm performs sample-quality assessment, reflecting that quality control is an integral component of routine diagnostic reporting and that low-quality samples may impair reliable AI learning and interpretation. At level L1, samples are classified as either NC or B-NHL. Accurate discrimination at this level is of particular clinical importance, as a false-positive lymphoma diagnosis has immediate and substantial implications for patient management.

Once B-NHL has been identified at L1, level L2 distinguishes CLL-like entities—CLL, monoclonal B cell lymphocytosis (MBL), and prolymphocytic leukemia (PL)—and hairy cell leukemia (HCL) from all other malignant lymphomas (AOL). This separation corresponds to pronounced structural differentiation observed in the unsupervised analysis (Fig 2: clusters 1–3). From a clinical perspective, these distinctions are prioritized: histopathological confirmation is not required for the diagnosis of CLL [50], and immunophenotyping is an essential diagnostic criterion for HCL [51].

At level L3, subtype differentiation within the AOL category focuses on the most common mature B cell lymphomas—mantle cell lymphoma (MCL), marginal zone lymphoma (MZL), lymphoplasmacytic lymphoma (LPL), and follicular lymphoma (FL)—to ensure comparability with previous studies [6,15]. Rare entities were grouped at level L4 as remaining B-NHL and were outside the scope of detailed automated subtype classification, acknowledging that comprehensive classification according to the 5th WHO edition would require inclusion of additional categories such as B lymphoblastic leukemia, diffuse large B cell lymphoma, Burkitt lymphoma, and immune deficiency–associated rare subtypes [47].

In summary, the diagnostic tree mirrors the four evaluated levels depicted in Fig 3 (L0–L3), with L4 representing residual categories outside the scope of detailed subtype analysis. This structure operationalizes an initial quality control step followed by clinically prioritized diagnostic decisions, aligning AI training and evaluation with established diagnostic workflows.

### Diagnostic performance of FlowXAI across hierarchy levels

The diagnostic performance of the FlowXAI system was evaluated on the MLL9F dataset comprising *N* = 19,493 three-tube samples using repeated cross-validation and was compared to a previously published deep learning–based approach [6]. Across diagnostic levels L2 and L3, the number of cases per malignant lymphoma entity ranged from 202 for HCL to 4,274 for CLL, resulting in pronounced class imbalance. The distribution of classes was as follows: B-NHL 45.3% and normal controls (NC) 54.7%; within B-NHL, CLL-like entities (CLL, PL, and MBL) accounted for 33.5%, HCL for 1.0%, and all other lymphomas (AOL) for 11.8%. Detailed class assignments are summarized in Table C in S1 Appendix. We retained the historical dataset label structure to preserve comparability with prior automated approaches, while acknowledging that this structure differs from the current WHO terminology.

**Fig 4:**
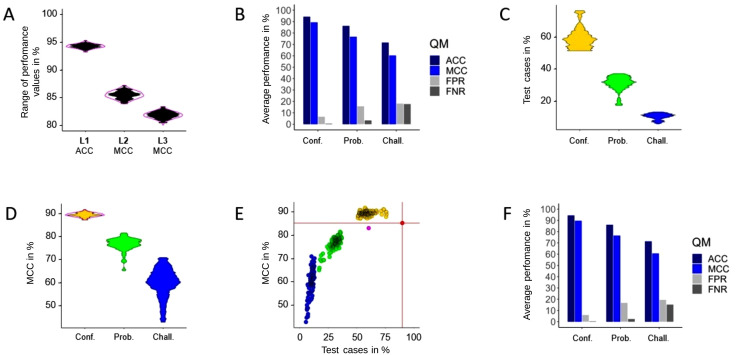
AI cross-validation for different diagnostic levels and different degrees of trustworthiness. **A)** FlowXAI performance was evaluated on the MLL9F dataset across 100 repeated class-balanced 80/20 train–test splits, with 15,594 training samples and 3,899 test samples per split and without TM-based exclusion of atypical samples, covering L1 discrimination of healthy controls from lymphoma, L2 classification of major lymphoma groups, and L3 differentiation of lymphoma diagnoses. Accuracy (ACC) is reported for level L1, and Matthews correlation coefficient (MCC) for levels L2 and L3. Mirrored-density plots (MD-plots) visualize the distributions of performance metrics; magenta outlines indicate Gaussian distributions where supported by internal statistical testing. Shown in the MD-plot, each test set comprised 20% of the dataset (*N* = 3,899 samples). The average performance for L1 is 94.2 ± 0.4% (ACC), for L2 85.4 ± 0.7% (MCC), and for L3 81.8 ± 0.7% (MCC). The estimated PDFs of ACC and MCC values are approximately Gaussian (magenta outline). **B)** Average performance metrics for level L3 stratified by FlowXAI’s self-assessed degrees of trustworthiness (confident, probable, challenging), aggregated across all cross-validation trials. All entities other than the NC samples were aggregated to B-NHL to calculate the quality measures ACC, FNR, and FPR. In addition, a contingency table was computed for each cross-validation trial, and all contingency tables were summed for each entry and provided in the Tables D-G in S1 Appendix. **C)** MD-plot of the number and distribution of test samples assigned to each degree of trustworthiness across cross-validation trials: confident (gold), probable (green), and challenging (blue). The estimated PDFs exhibit bimodality and illustrate that the training data are not homogeneous, indicating that preselection by chance influences performance. **D)** MD-plot showing the FlowXAI performance as the estimated distribution of MCC values for level 3 stratified by degree of trustworthiness. The estimated PDF of MCC values for the confident degree is approximately Gaussian (magenta outline), whereas the distribution for the probable degree is slightly skewed and that for the challenging degree has a large variance. **E)** Comparison between the deep learning AI and FlowXAI. Relationship between the proportion of test samples assigned to each degree of trustworthiness and corresponding MCC values at level L3. Each triplet of points represents one cross-validation trial and sums to 100% of test samples. Performance of the deep learning baseline is shown for comparison (magenta, 83% (MCC) for 12% of data samples (N = 2,348)). The y-axis depicts the MCC value in percent for each cross-validation trial, and the x-axis shows the percentage of test data samples assigned to each degree of trustworthiness. **F)** Average performance metrics for level L3 after exclusion of atypical samples from the training sets using Tile Mining (TM). Abbreviations: PDF: Probability density function, ACC = accuracy, MCC = Matthews correlation coefficient, FPR = false positive rate, FNR = false negative rate, Conf = confident, Prob = probable, Chall = challenging.

Without TM-based exclusion of atypical training samples, FlowXAI distinguished NC samples (*N* = 2,133) from B-NHL samples (*N* = 1,766) at diagnostic level L1 with an average accuracy of 94.2 ± 0.4%. At level L2, classification of CLL-like entities, HCL, and AOL against NC samples yielded an average MCC of 85.4 ± 0.7%. At level L3, differentiation among AOL subtypes resulted in an average MCC of 81.8 ± 0.7%. The distributions of performance metrics across the 100 cross-validation trials are shown as mirrored-density plots (MD-plots) in Fig 4a, illustrating stable performance across random partitions.

Beyond overall performance, FlowXAI provides a self-assessed degree of trustworthiness for each diagnostic decision, categorized as confident, probable, or challenging. Incorporating this stratification, performance metrics were recalculated for level L3 decisions (Fig 4b). Across cross-validation trials, FlowXAI assigned between 52% and 73% of test samples to the confident category, 20% to 35% to the probable category, and 7% to 12% to the challenging category (5th to 95th percentile range; Fig 4c). Notably, the distribution of test samples across trustworthiness categories exhibited bimodality, reflecting heterogeneity across random splits. A detailed look reveals 16 cross-validation cycles in which less than 8% of the predictions are assigned to the challenging and probable degree of trustworthiness, with correspondingly more predictions self-assessed by FlowXAI as confident. This heterogeneous pattern of the cross-validation results underscores the necessity of conducting at least 100 cross-validation trials because the results may appear superior (or inferior) by chance.

Performance within the confident category showed consistently high MCC values, ranging from 88% to 91% (median 90%), with an approximately Gaussian distribution (magenta frame) (Fig 4d). The probable category yielded intermediate performance (median MCC 77%), while the challenging category exhibited lower and more variable performance (median MCC 60%). These results indicate that FlowXAI’s internal self-assessment is informative with respect to diagnostic reliability.

Direct comparison with the deep learning AI system revealed that the overall performance of FlowXAI, aggregated across confident and probable predictions, was comparable to that of the neural network approach (MCC 85.1% versus 83%; Fig 4e). Importantly, this level of performance was achieved for 89% of test samples (*N* = 3,488). Within the confident category alone, FlowXAI yielded higher MCC values than the deep learning baseline. Of note, the previously published deep neural network–based AI system reported hematologist‐level classification of mature B‐cell neoplasm based on only a single train–test split; however, this performance was not stratified by self-assessed diagnostic reliability.

**Fig 5:**
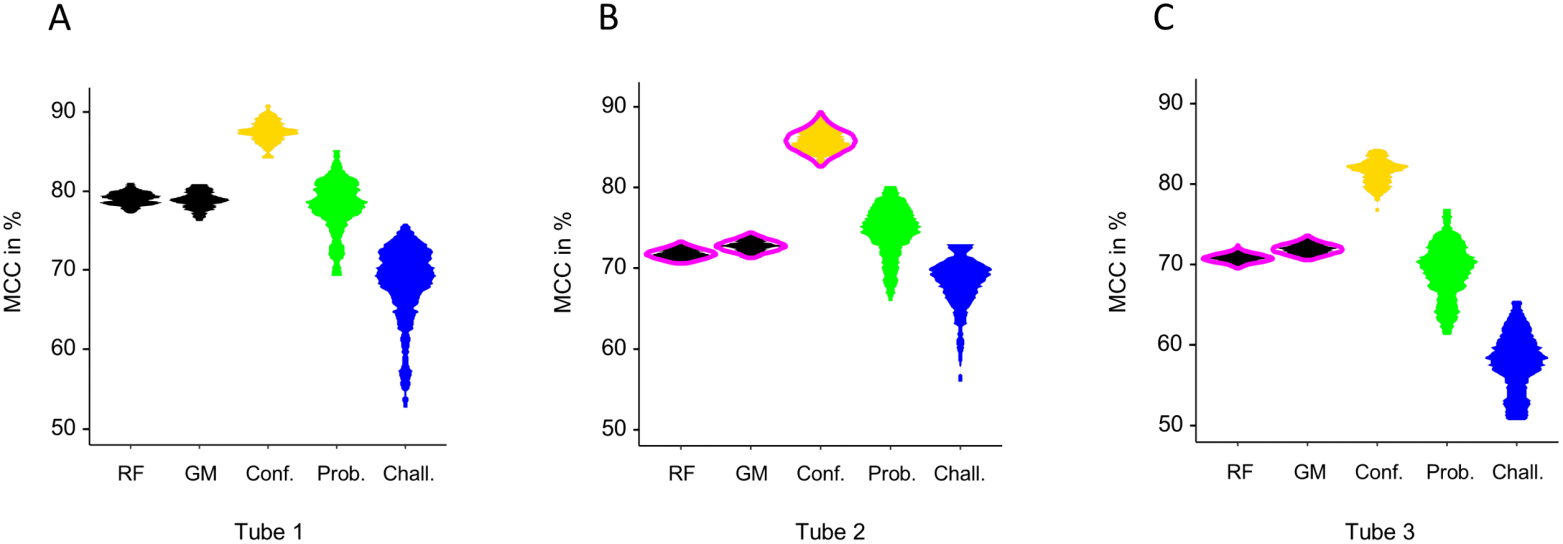
Benchmarking FlowXAI against conventional single-tube baselines on MLL9F at hierarchy level L3. Benchmarking was performed using the same 100 repeated class-balanced 80/20 train-test splits as in Fig 4A (15,594 training samples and 3,899 test samples per repetition). For each tube, sample-level flowFP fingerprints were generated and used as input to a random forest (RF) and a glmnet classifier (GM). MD-plots show the distributions of held-out Matthews correlation coefficient (MCC) values across the 100 test folds for the two baseline classifiers and for FlowXAI stratified by trustworthiness: confident (gold), probable (green), and challenging (blue). Where shown, magenta outlines denote Gaussian fits to the empirical MCC distributions. Mean ± SD MCC values were: **A)** tube 1: RF 79% ± 1%, GM 79% ± 1%, FlowXAI confident 88% ± 1%, probable 78% ± 3%, challenging 68% ± 5%. **B)** tube 2: RF 72% ± 1%, GM 73% ± 1%, FlowXAI confident 86% ± 1%, probable 74% ± 3%, challenging 68% ± 3%. **C)** tube 3: RF 71% ± 1%, GM 72% ± 1%, FlowXAI confident 81% ± 2%, probable 69% ± 3%, challenging 56% ± 6%. Abbreviations: MCC = Matthews correlation coefficient; MD = mirrored density, RF = random forest, GM = multinomial elastic-net classifier (glmnet), Conf, confident, Prob=Probable, Chall=challenging.

Within FlowXAI, the native trustworthiness degrees were informative with respect to diagnostic reliability. Because the published comparator systems considered here do not provide directly analogous confident, probable, and challenging outputs for the same task, this analysis is presented as a within-framework validation rather than as a cross-algorithm benchmark (see Text D in S1 Appendix: Literature evaluation for seeking benchmark algorithms). To provide a conventional single-tube reference, we benchmarked random forest [52,53] and multinomial elastic-net classifier (GM) [54,55] trained on flowFP fingerprints [56] against FlowXAI at hierarchy level 3 across the same 100 repeated train-test splits (Fig 5), see Text G in S1 Appendix for details. Mean ± SD test MCC values for random forest were 0.79 ± 0.01, 0.72 ± 0.01, and 0.71 ± 0.01 for tubes 1–3, and for GM 0.79 ± 0.01, 0.73 ± 0.01, and 0.72 ± 0.01, respectively (Fig 5A,5B,5C). The FlowXAI confident stratum reached 0.88 ± 0.01, 0.86 ± 0.01, and 0.81 ± 0.02 across tubes 1–3. Because trustworthiness categories are native outputs of FlowXAI, the present analysis should be interpreted as a further validation of the framework’s internal trustworthiness stratification rather than as a direct algorithm-to-algorithm comparison.

When TM-based elimination of atypical samples was applied to the training sets, a modest improvement in accuracy and MCC was observed across all degrees of trustworthiness (Fig 4f). This effect was most pronounced in the challenging category, where false negative rates decreased. Detailed entity-specific contingency tables for each cross-validation trial are provided in Tables D–G in S1 Appendix.

Overall, these results show that FlowXAI yields stable diagnostic performance across clinically prioritized decision levels, provides informative self-assessment of diagnostic trustworthiness, and achieves performance comparable to deep learning–based systems while offering transparent, case-level stratification of reliability.

### Reduction of training data and extended validations of FlowXAI

**Fig 6:**
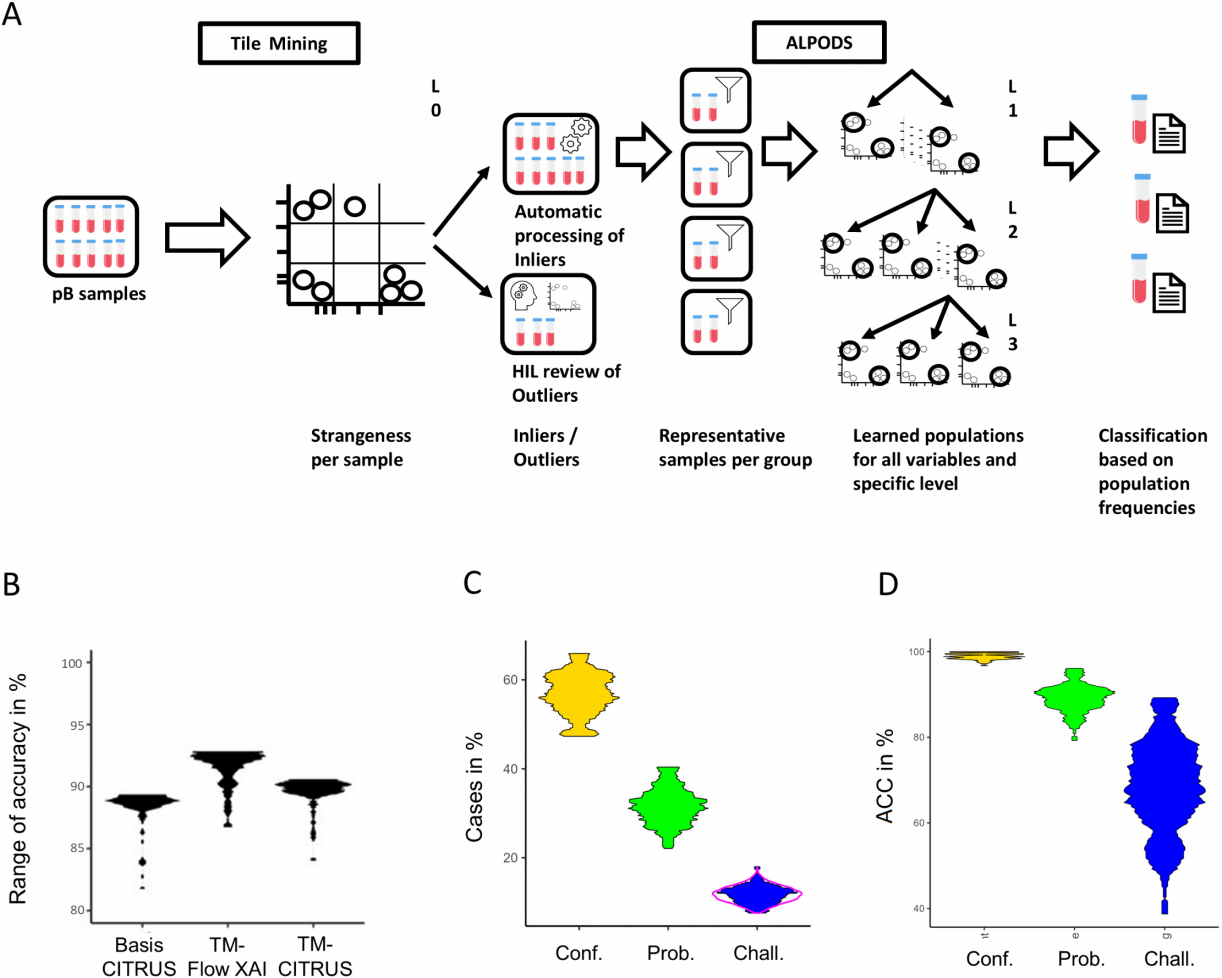
Workflow and validation of FlowXAI in combination with TM. **A)** Schematic overview of the FlowXAI workflow integrating Tile Mining (TM) for unsupervised sample-quality assessment and representative training sample selection, followed by supervised learning using a committee of ALPODS experts. Strangeness per sample and outlier assignment corresponds to physician’s judgement on sample quality. Human-in-the-loop (HIL) review is needed for an atypical sample to determine if the sample is usable. Classification decisions are based on cell populations. The detailed construction of FlowXAI using a committee of ALPODS experts is illustrated in Fig A in S1 Appendix. **B)** MD-plot of accuracy distributions for level L1 (NC versus B-NHL) comparing FlowXAI and the population classifier CITRUS, with and without TM-based filtering of training samples. Analysis is restricted to tube 1. **C-D)** Performance of FlowXAI on the independent external benchmark dataset (PUM2) from a second diagnostic laboratory evaluated in 100 cross-validation trials using 200 training samples per trial (100 NC, 100 B-NHL), all drawn from TM-identified typical samples, see also Text C in S1 Appendix. **C)** MD-plot of number of test samples assigned to each degree of trustworthiness across cross-validation trials. The estimated PDFs do not exhibit bimodality. For all different lymphoma subgroups FlowXAI classified on average 57% of N = 117 test samples as confident, 31% as probable*,* and 12% as challenging. **D)** MD-plots show the distribution of accuracy values stratified by degree of trustworthiness*.* The magenta frame indicates a Gaussian distribution. Abbreviations: Conf = confident; Prob = probable; Chall = challenging, pB = peripheral blood.

Small class sizes either due to short collection periods of diagnostic data or rare lymphoma subtypes substantially limit the applicability of data-intensive learning approaches. To address this constraint, TM was integrated into FlowXAI to enable representative sample selection and training data reduction (Fig 6a; Text A and H in S1 Appendix).

The outlier discrimination capability of TM was first validated using a randomly selected subset of 100 samples classified as atypical. Manual inspection by experienced immunophenotyping experts confirmed that 96 of these samples exhibited technical or biological alterations that would impair reliable diagnostic interpretation, whereas only 4% were judged usable across all tubes for routine diagnostic reporting (Fig B in S1 Appendix). These findings support the role of TM as a general sample-quality assessment applicable pre-diagnostically.

Using FlowXAI with TM (see Fig.6A for workflow), the training set was reduced to 512 representative samples, comprising 256 normal controls and 256 B-NHL cases equally distributed across lymphoma entities. Despite this substantial reduction in training data, FlowXAI classified 49% of the test samples as confident, 30% as probable, and 21% as challenging. For the 7,684 test samples assigned to the confident category, an MCC of 87% was achieved at diagnostic level L3, compared with 62% for probable and 23% for challenging cases. Detailed contingency tables for these results are provided in Tables H–J in S1 Appendix. Notably, when restricted to confident predictions, FlowXAI outperformed the deep learning–based system in the automated classification of six lymphoma groups (MCC 87% versus 83%), despite being trained on only 512 samples compared with 18,274 samples used for neural network training.

To further contextualize the benefit of TM-based representative sampling, FlowXAI performance at level L1 was benchmarked against the cluster identification, characterization, and regression algorithm (CITRUS) [1]. Among available population-based approaches for supervised analysis [2,57–61], CITRUS was selected because of its conceptual similarity to population-driven immunophenotyping and its previously demonstrated performance relative to other methods [1,30]. When TM was applied prior to training, CITRUS performance improved, but FlowXAI achieved the highest median accuracy across 100 cross-validation trials (Fig 6b; Text E in S1 Appendix).

Finally, FlowXAI was evaluated on an independent external benchmark dataset (PUM2) comprising 638 samples collected at a separate diagnostic center using a different antibody panel and two-tube configuration. TM identified 517 samples as typical and 121 as atypical. In 100 cross-validation trials, training sets consisted of 100 normal controls and 100 B-NHL cases drawn exclusively from typical samples (30 CLL, 15 MCL, 10 FL, 10 MZL, 5 LPL, 10 HCL, 10 MBL, and 10 NS), while test sets included both typical and atypical cases. 100 trials of cross-validation were performed on the remaining test sets of 317 typical samples.

At diagnostic level L1, FlowXAI classified 57% of test samples as confident, 31% as probable, and 12% as challenging. Accuracy reached 99% for confident, 89% for probable, and 69% for challenging cases (Fig 6c, 6d). Even with this limited training set size, FlowXAI achieved highly accurate discrimination of B-NHL from normal controls in more than half of typical cases. Performance on atypical samples remained high, with confident and probable classifications reaching accuracies of 98% and 90%, respectively (Text C and Fig F in S1 Appendix).

In addition to quantitative performance, FlowXAI provides case-specific explanations by selecting diagnostically relevant cell populations and expression patterns in a human-interpretable manner. Representative examples of correct and incorrect classifications, including false-positive and false-negative HCL cases, are presented in Text B in S1 Appendix and in Figs C–E in S1 Appendix. Finally, we provide an interactive evaluation of model-generated reports: https://plait.mathematik.uni-marburg.de/.

### Trustworthiness validation

FlowXAI’s trustworthiness output was informative with respect to diagnostic reliability [62,63]. The observed correctness rate increased from 79.5% (95% CI [79.3%, 79.8%]) for challenging cases to 86.9% (95% CI [86.6%, 87.1%]) for probable cases and 94.3% (95% CI [94.2%, 94.4%]) for confident cases. Overall, calibration of predicted correctness showed low average miscalibration (expected calibration error - ECE 0.024; Brier score 0.089). In the upper reliability bins, calibrated predicted correctness ranged from 90.1% to 90.6%, whereas observed correctness ranged from 93.5% to 94.0%, indicating conservative calibration in the highest-confidence region.

Selective one-vs-rest ROC and precision-recall analyses based on the ordered FlowXAI trust strata showed strongest selective discrimination for NC, CLL-like, and HCL, intermediate performance for MZL, and limited selective utility for MCL, FL, and LPL (Text F in S1 Appendix Selective one-vs-rest receiver operating characteristic (ROC) and precision-recall analysis from FlowXAI degrees of trustworthiness Figs G–H in S1 Appendix).

## Discussion

The FlowXAI system was designed as a supportive tool for assisting and teaching the diagnostic process of B-NHL using multiparameter flow cytometry–based immunophenotyping. To ensure that diagnostic performance was assessed against clinically relevant reference standards, both datasets were accompanied by comprehensive clinical evidence, including genetic and histopathological information, as previously published [6,7]. Following the initial descriptions of Hodgkin lymphoma in 1832 and non-Hodgkin lymphoma in 1925, multiple classification systems coexisted until the Revised European and American Lymphoma (R.E.A.L.) classification was introduced in 1994, incorporating immunopathological aspects for the first time. This framework was subsequently replaced by the World Health Organization (WHO) classification, with its 5th edition published in 2022. Importantly, the term B prolymphocytic leukemia was eliminated in WHO-HAEMS5, and prolymphocytic leukemia is now considered a progression subtype of CLL [47]. These historical developments underscore that clinical labels are authoritative and indispensable for patient care, yet subject to revision as medical knowledge evolves.

To better understand how such clinically defined entities are reflected in multiparameter flow cytometry (MFC) data, we deliberately adopted an unbiased MFC-based perspective on the structural embodiment of lymphoma diagnoses through the self-organization of data by swarm intelligence. This approach was chosen to elucidate the data-intrinsic taxonomy available to AI systems and to reduce the risk of learning spurious correlations, a known limitation of deep neural network–based models [64]. Using unsupervised machine learning via the Databionic swarm [38], we identified high-dimensional structures in the data that were visualized as topographic maps. As shown previously, such unsupervised approaches can correspond well with disease entities associated with divergent treatment decisions [38,42,46]. In the present study, these analyses revealed a clear separation between normal controls and lymphoma samples, as well as distinct structures corresponding to CLL-like entities and HCL. However, other lymphoma entities were not clearly separable when analysis was restricted to the specific antibody panels used. Importantly, the absence of a distinct structure in this setting does not imply the absence of a valid disease entity; rather, it reflects the limited discriminatory power of the selected immunophenotypic markers. It has been shown that addition of antigens in a given diagnostic panel may enhance the separability for certain lymphoma subtypes such as CD43, CD200 and ROR1 for B CLL [7,9,65–67], or transferrin receptor in high grade lymphomas [68]. However, an optimal antibody panel for B cell immunophenotyping has not been defined yet [9] but resolution could be enhanced with identification of further discriminatory antigens in future [65].

The overall diagnostic performance of FlowXAI was evaluated using 100 cross-validation trials with class-balanced train–test splits [32,33,69] and compared to a deep neural network system [6], CITRUS [1] and conventional fingerprint based baselines [52–56]. Overall, the results show that FlowXAI performs within the range and sometimes above established AI and machine-learning approaches for flow cytometry–based B-NHL classification. Its main contribution, however, lies not in aggregate classification performance alone, but in combining competitive performance with hierarchical multi-tube decision support, interpretable case-level outputs, and native trustworthiness assessment for identifying cases that may require closer expert review. We did not perform dedicated perturbation experiments for compensation, fluorescence drift, lot-to-lot variability, or instrument-specific effects although the MLL9F dataset was measured on several instruments. Accordingly, we restrict our claims to the robustness directly evaluated here: repeated held-out testing within MLL9F with limited instrument variability, and cross-site benchmarking on PUM2 under routine laboratory preprocessing. Dedicated technical robustness analyses remain an important topic for future work.

A key methodological contribution of FlowXAI lies in its ability to substantially reduce training data requirements: by integrating TM for unsupervised sample-quality assessment and representative case selection, reliable prototype-based learning was achieved using only a small number of samples per lymphoma entity. This property is particularly relevant for rare lymphoma subtypes, where large training cohorts are often unavailable. TM defines structural atypicality relative to the reference cohort and the measured antibody-panel space; it cannot determine whether an atypical profile reflects a technical artefact, genuine biological variation, or insufficiently represented lymphoma phenotype. Consequently, excluding TM-atypical cases from representative-case selection and supervised training may stabilize learning for common phenotypes but may also reduce the representation of heterogeneous entities in the learned decision rules. TM should therefore be interpreted as a human-in-the-loop curation and flagging step rather than as an autonomous exclusion criterion.

In the present study, atypical cases require expert review and were excluded only from representative-case selection and supervised training, whereas all independent test sets retained both typical and atypical cases. To quantify the effect of TM on class composition, we report the numbers of typical and atypical cases for each lymphoma entity in MLL9F and PUM2 and cross-reference the entity-specific performance tables in Tables C-J and Text C in S1 Appendix. We therefore interpret TM as a training-stabilization step and human-in-the-loop flagging mechanism, rather than as a method for excluding clinically difficult cases from evaluation. Because the TM threshold is empirically estimated from cohort-specific strangeness distributions, its numerical value should not be interpreted as a universal biological cutoff. Rather, TM provides a representation of population-level organization within the measured marker-pair space, while the exact threshold may vary with cohort composition, preprocessing, antibody panel, and tube configuration. Future work should therefore evaluate TM threshold sensitivity prospectively and across matched-panel datasets.

Uneven performance across lymphoma entities does not appear to be explained primarily by class prevalence. In the one-vs-rest ROC analyses, HCL showed better separability than LPL and MZL (Figs G and H in S1 Appendix), and in additional experiments using the same number of training sample files per class (*N* = 32; Tables H–J in S1 Appendix), the relative performance differences between entities persisted. Together, these findings indicate that entity-specific performance is driven mainly by the biological separability of lymphoma entities in the measured immunophenotypic space and by the discriminatory information content of the available antibody panel. This interpretation is consistent with the structural-embodiment analysis in Fig 2, which showed that some lymphoma entities form clearer emergent structures than others. Accordingly, reduced performance for some entities should not be interpreted solely as algorithmic failure, but also as a consequence of limited panel information content and intrinsic immunophenotypic overlap between biologically related lymphoma entities. Clinically, these results suggest that FlowXAI is most reliable for immunophenotypically distinct entities, whereas more overlapping categories require more cautious interpretation and continued integration with morphology, molecular findings, and expert review.

Beyond performance and data efficiency, FlowXAI was explicitly designed to address the requirements of real-world decision support and clinical education through self-explanation. This self-explanatory capability comprises two complementary components.

First, FlowXAI provides a self-assessed degree of trustworthiness for each diagnostic decision, which can be viewed as analogous to established diagnostic scoring systems used in hematology [28]. Validation of the association between the assigned degrees of trustworthiness and observed diagnostic performance supports the clinical relevance of this stratification. Probable and challenging predictions carried a substantially higher error risk, whereas confident predictions were associated with markedly higher observed diagnostic reliability. Selective one-vs-rest ROC and precision-recall analyses indicate that FlowXAI degrees of trustworthiness have entity-specific clinical value: high-trustworthiness predictions are most actionable for NC, CLL-like cases, and HCL; MZL shows intermediate decision-support utility; and MCL, FL, and LPL remain predominantly expert-review entities.

Second, FlowXAI identifies and visualizes diagnostically relevant cell populations, enabling interactive inspection and explanation of AI-driven decisions. Such direct interaction between clinicians and data-driven AI systems has been shown to foster effective human–AI collaboration [70]. From both informatics and social perspectives, self-explanatory clinical decision support systems are considered essential for trust, reliance, and responsible use [24,71,72]. Given the increasing influence of AI in clinical medicine, preparing clinicians to critically engage with AI-supported diagnostics is becoming an inevitable and necessary task [73]. To our knowledge, the design and deployment of a self-explaining symbolic AI system for lymphoma immunophenotyping has not yet been carried out.

Several limitations of the present study have to be acknowledged. First, the identified immunophenotypic structures in data are panel-specific and it requires future work to investigate whether they generalize to other antibody configurations. Diagnostic performance depends on flow cytometry data quality, and not all WHO-defined lymphoma entities were included due to limited sample sizes. Further performance optimization of FlowXAI may be achieved by incorporating advances in AI based MFC lymphoma classification and suitable B cell selection algorithms (see Text D in S1 Appendix: Literature evaluation for seeking benchmark algorithms). Notably, unselective data approaches may permit the discovery of novel putative target cell populations even beyond the well-known pathologic B cells following the basic principle of knowledge discovery.

Second, evaluation of a fixed-model in an independent cohort acquired with an identical or matched antibody panel would further strengthen assessment of model generalizability and remains an important next step beyond the scope of the current work.

Third, TM is not intended to substitute conventional laboratory quality-control procedures such as instrument monitoring, compensation validation, or staining controls. Instead, TM operates at the level of the sample file and evaluates whether the multivariate event structure of a case is typical relative to the reference cohort after standard preprocessing. The TM threshold and the number of representative training samples were treated as pragmatic design parameters rather than biologically fixed constants. We therefore make no claim that these parameters are uniquely optimal.

FlowXAI is intended as a decision-support and triage tool for expert users, rather than as a fully autonomous diagnostic system. Diagnostic integration with morphology, molecular findings, and expert review remains necessary for specific clinical distinctions. Taken together, FlowXAI enables accurate diagnosis of B-NHL from a comparatively small amount of non-standardized flow cytometry data, achieving performance levels equal to or exceeding those of deep learning–based and other benchmark algorithms. The system is self-explanatory and knowledge-based by design, and it explicitly integrates clinical diagnostic priorities while respecting the limitations of immunophenotypic data. Its self-assessment capabilities facilitate a transparent and interactive use by human experts. Through its transparent self-assessment and interactive explanations, FlowXAI offers a practical foundation for an open-access, AI-supported teaching platform in lymphoma immunophenotyping.

### Ethics statement

The study used previously collected clinical flow cytometry data from MLL, Munich Leukemia Laboratory and University Hospital Marburg. The use of these data for methodological evaluation was approved by the institutional review board/ethics committee in previously cited papers and was conducted in accordance with the Declaration of Helsinki. This study only conducted a retrospective analysis of routinely collected clinical data and all analyses were performed on de-identified data. In addition, the ethics committee of the University of Marburg approved the study (100/21). All flow cytometry files were processed in de-identified form, and no directly identifying patient information is included in the shared data or in the online demonstration.

## Supporting information

S1 Appendix S1 Appendix component legends. Table A. Confusion matrix between data-driven structures identified by the Databionic swarm and clinical lymphoma labels for Fig 2A and 2B. Table B. Confusion matrix between structures in data identified after Tile Mining–based exclusion of structurally atypical samples and clinical lymphoma labels for Fig 2C and 2D. Table C. Designation of classes and lymphoma categories. Table D. Average contingency table in L3 for the probable and confident trustworthiness degrees. Table E. Average contingency table in L3 for the confident trustworthiness degree. Table F. Average contingency table in L3 for the probable trustworthiness degree. Table G. Average contingency table in L3 for the challenging trustworthiness degree. Table H. Contingency table for 512 training cases in L3 for the confident trustworthiness degree. Table I. Contingency table for 512 training cases in L3 for the probable trustworthiness degree. Table J. Contingency table for 512 training cases in L3 for the challenging trustworthiness degree. Fig A. Exemplary decision logic of the ALPODS expert committee for the PUM2 dataset. Fig B. Illustration of outlier samples using dot plots. Fig C. Explaining FlowXAI diagnostic decisions. Fig D. Explanations for HCL misdiagnosis using bivariate dot plots in log scale - Three false positive results. Fig E. Explanations for HCL misdiagnosis using bivariate dot plots in log scale - Three false negative results. Fig F. FlowXAI-based lymphoma classification results for atypical cases in the PUM2 dataset. Fig G. FlowXAI one-vs-rest selective ROC curves by diagnosis. Fig H. FlowXAI one-vs-rest selective precision-recall curves by diagnosis. Fig I. Tube-specific TM strangeness distributions and robust Gaussian thresholding for the MLL9F dataset. Fig J. Tube-specific TM strangeness distributions and robust Gaussian thresholding for the external PUM2 dataset. Text A. ALPODS expert committee, Tile Mining-based sample selection, and model construction. Text B. FlowXAI explainability. **Text C.** Atypical cases in the PUM2 dataset. **Text D.** Literature evaluation for benchmark algorithms. **Text E.** Benchmarking FlowXAI with CITRUS. **Text F.** Selective one-vs-rest ROC and precision-recall analysis from FlowXAI degrees of trustworthiness. **Text G.** Conventional single-tube baselines based on flowFP fingerprints. **Text H.** Strangeness distributions and Tile Mining parameters. Supplementary References: References cited in S1 Appendix.
(DOCX)

S1 Checklist Completed TRIPOD+AI reporting checklist. The checklist was reproduced from Collins GS, Moons KGM, Dhiman P, and colleagues. The official checklist is available from https://www.tripod-statement.org/wp-content/uploads/2019/12/TRIPODAI_checklist.pdf. The checklist is licensed under the Creative Commons Attribution 4.0 International Licence (CC BY 4.0; https://creativecommons.org/licenses/by/4.0/). The original checklist wording was not modified; manuscript locations and study-specific responses were added by the authors.
(PDF)

S2 Checklist TRIPOD+AI statement: updated guidance for reporting clinical prediction models that use regression or machine learning methods. BMJ. 2024;385:e078378. https://doi.org/10.1136/bmj-2023-078378.
(PDF)

S1 Data S1_Data_PUM2_DecisionLogic.zip. (ZIP)
