# Supplementary material for: Self-explaining artificial intelligence for the classification of B cell non-Hodgkin lymphoma: A diagnostic decision support study
Source: PLoS Med. 2026 Jul 13;23(7):e1004889. doi: 10.1371/journal.pmed.1004889 (PMC13421771; doi:10.1371/journal.pmed.1004889)
Supplement: S1 Appendix — Table A. Confusion matrix between data-driven structures identified by the Databionic swarm and clinical lymphoma labels for Fig 2A and 2B. Table B. Confusion matrix between structures in data identified after Tile Mining–based exclusion of structurally atypical samples and clinical lymphoma labels for Fig 2C and 2D. Table C. Designation of classes and lymphoma categories. Table D. Average contingency table in L3 for the probable and confident trustworthiness degrees. Table E. Average contingency table in L3 for the confident trustworthiness degree. Table F. Average contingency table in L3 for the probable trustworthiness degree. Table G. Average contingency table in L3 for the challenging trustworthiness degree. Table H. Contingency table for 512 training cases in L3 for the confident trustworthiness degree. Table I. Contingency table for 512 training cases in L3 for the probable trustworthiness degree. Table J. Contingency table for 512 training cases in L3 for the challenging trustworthiness degree. Fig A. Exemplary decision logic of the ALPODS expert committee for the PUM2 dataset. Fig B. Illustration of outlier samples using dot plots. Fig C. Explaining FlowXAI diagnostic decisions. Fig D. Explanations for HCL misdiagnosis using bivariate dot plots in log scale - Three false positive results. Fig E. Explanations for HCL misdiagnosis using bivariate dot plots in log scale - Three false negative results. Fig F. FlowXAI-based lymphoma classification results for atypical cases in the PUM2 dataset. Fig G. FlowXAI one-vs-rest selective ROC curves by diagnosis. Fig H. FlowXAI one-vs-rest selective precision-recall curves by diagnosis. Fig I. Tube-specific TM strangeness distributions and robust Gaussian thresholding for the MLL9F dataset. Fig J. Tube-specific TM strangeness distributions and robust Gaussian thresholding for the external PUM2 dataset. Text A. ALPODS expert committee, Tile Mining-based sample selection, and model construction. Text B. FlowXAI explainabi [file pmed.1004889.s001.docx]

**Supplementary information (SI)**

**Self-explaining artificial intelligence for the classification of B cell non-Hodgkin lymphoma**

Michael C. Thrun^1,5^*, Jörg Hoffmann^2^, Stefan W. Krause^3^, Peter Krawitz^4^, Quirin Stier^1^, Andreas Neubauer^2^, Cornelia Brendel^2&^ and Alfred Ultsch^1&^

^1^Mathematics and Computer Science, Philipps University Marburg, Hans-Meerwein-Strasse 6, 35032 Marburg, Germany

^2^Department of Hematology, Oncology and Immunology, Philipps University Marburg, University Hospital Giessen and Marburg, Baldingerstrasse, 35043 Marburg, Germany

^3^Department of Medicine 5, Universitätsklinikum Erlangen, Ulmenweg 18, 91054 Erlangen, Germany

^4^Institute for Genomic Statistics and Bioinformatics, University Bonn, Venusberg-Campus 1, 53127 Bonn, Germany

^5^IAP-GmbH Intelligent Analytics Projects, In den Birken 10A, 29352 Adelheidsdorf, Germany

***Corresponding author**

Michael C. Thrun (MCT)

***** mthrun@informatik.uni-marburg.de

^&^AU and CB contributed equally and share the last author title.

**S1 Appendix component legends**

**Table A.** Confusion matrix between data-driven structures identified by the Databionic swarm and clinical lymphoma labels for Fig 2A and 2B.

**Table B.** Confusion matrix between data-driven structures identified after Tile Mining–based exclusion of structurally atypical samples and clinical lymphoma labels for Fig 2C and 2D.

**Table C.** Designation of classes and lymphoma categories.

**Table D.** Average contingency table in L3 for the probable and confident trustworthiness degrees.

**Table E.** Average contingency table in L3 for the confident trustworthiness degree.

**Table F.** Average contingency table in L3 for the probable trustworthiness degree.

**Table G.** Average contingency table in L3 for the challenging trustworthiness degree.

**Table H.** Contingency table for 512 training cases in L3 for the confident trustworthiness degree.

**Table I.** Contingency table for 512 training cases in L3 for the probable trustworthiness degree.

**Table J.** Contingency table for 512 training cases in L3 for the challenging trustworthiness degree.

**Fig A.** Exemplary decision logic of the ALPODS expert committee for the PUM2 dataset

**Fig B.** Illustration of outlier samples using dot plots.

**Fig C.** Explaining FlowXAI diagnoses decisions.

**Fig D.** Explanations for HCL misdiagnosis using bivariate dot plots in log scale - Three false positive results.

**Fig E.** Explanations for HCL misdiagnosis using bivariate dot plots in log scale - Three false negative results.

**Fig F.** FlowXAI-based lymphoma classification results for atypical cases in the PUM2 dataset.

**Fig G.** FlowXAI one-vs-rest selective ROC curves by diagnosis.

**Fig H.** FlowXAI one-vs-rest selective precision-recall curves by diagnosis.

**Fig I.** Tube-specific TM strangeness distributions and robust Gaussian thresholding for the MLL9F dataset.

**Fig J.** Tube-specific TM strangeness distributions and robust Gaussian thresholding for the external PUM2 dataset.

**Text A.** ALPODS expert committee, Tile Mining-based sample selection, and model construction.

**Text B. FlowXAI explainability.**

**Text C. Atypical cases in the PUM2 dataset.**

**Text D. Literature evaluation for benchmark algorithms.**

**Text E. Benchmarking FlowXAI with CITRUS.**

**Text F. Selective one-vs-rest ROC and precision-recall analysis from FlowXAI categories degrees of trustworthiness**

**Text G. Conventional single-tube baselines based on flowFP fingerprints.**

**Text H. Strangeness distributions and Tile Mining parameters.**

**Supplementary References. References cited in S1 Appendix.**

**Table A** Confusion matrix between data-driven structures identified by the Databionic swarm and clinical lymphoma labels for Fig 2A and 2B. Clinical labels reflect routine diagnostic decisions and are shown to assess correspondence between unsupervised structural embodiment and clinically assigned lymphoma entities.

|  | **CLL** | **FL** | **HCL** | **LPL** | **MBL** | **ML** | **MZL** | **NC** | **PL** |
| --- | --- | --- | --- | --- | --- | --- | --- | --- | --- |
| **Cluster1** | 2990 | 116 | 114 | 217 | 946 | 307 | 597 | 3 | 548 |
| **Cluster2** | 9 | 62 | 10 | 8 | 41 | 57 | 32 | 2995 | 10 |
| **OutlierGroup** | 0 | 8 | 0 | 5 | 4 | 5 | 2 | 0 | 3 |
| **OutlierGroup** | 0 | 0 | 0 | 0 | 10 | 1 | 3 | 0 | 2 |
| **VariousOutlier** | 1 | 0 | 4 | 6 | 5 | 2 | 3 | 2 | 0 |

**Table B** Confusion matrix between data-driven structures identified after Tile Mining–based exclusion of structurally atypical samples and clinical lymphoma labels for Fig 2C and 2D.This comparison illustrates improved structural coherence after sample-quality filtering, while highlighting entities that remain not clearly separable given the antibody panel used.

|  | **CLL** | **FL** | **HCL** | **LPL** | **MBL** | **MCL** | **MZL** | **PL** |
| --- | --- | --- | --- | --- | --- | --- | --- | --- |
| **Cluster1** | 9 | 166 | 64 | 490 | 132 | 140 | 747 | 159 |
| **Cluster2** | 491 | 1 | 0 | 21 | 365 | 75 | 23 | 264 |
| **Cluster3** | 0 | 1 | 82 | 2 | 0 | 1 | 25 | 1 |
| **Outliers1** | 0 | 1 | 0 | 0 | 1 | 1 | 0 | 0 |
| **Outliers2** | 0 | 0 | 0 | 0 | 2 | 0 | 0 | 0 |
| **Outliers3** | 0 | 0 | 0 | 0 | 0 | 2 | 0 | 0 |
| **Outliers4** | 0 | 0 | 0 | 0 | 0 | 2 | 0 | 0 |

**Table C Designation of classes and lymphoma categories**

| **Class No** | **Entity** | **MLL9F Typical No.** | **MLL9F Atypical No.** | **PUM2**  **Typical No.** | **PUM2**  **Atypical No.** |
| --- | --- | --- | --- | --- | --- |
| **1** | NC | 8,692 | 1,967 | 177 | 48 |
| **2** | CLL | 3,325 | 949 | 173 | 95 |
| **3** | MCL | 237 | 60 | 16 | 20 |
| **4** | FL | 195 | 38 | 10 | 5 |
| **5** | MZL | 867 | 170 | 11 | 8 |
| **6** | LPL | 555 | 120 | 6 | 4 |
| **7** | HCL | 171 | 31 | 11 | 11 |
| **(2*)** | DLBCL | 0 | 0 | 2 | 5 |
| **(2*)** | BL | 0 | 0 | 1 | 1 |
| **2** | MBL | 1,267 | 247 | 12 | 3 |
| **2** | PL | 432 | 121 | 0 | 0 |
| **(2*)** | HCLv | 50 | 10 | 0 | 0 |
| **(2*)** | NOS | 0 | 0 | 14 | 5 |

PUM2 = Philipps-University Marburg data, MLL9F = Munich Leukemia Laboratory data. CLL, MBL, and PL are classified as CLL-like. In the first column, the table shows the diagnoses that were combined with the class numbers used for the learning task for the MLL9F data. Because of the small number of patients, only NC and B-cell lymphoma samples were used for supervised learning in the PUM2 dataset, and the HCLV entity was disregarded[1]. NOS = not otherwise specifiable, NC = normal controls. *: these diagnoses were considered for distinguishing NC and B-NHL samples in L1 but not in L2 or L3.

All contingency tables (TabD-J) summarize diagnostic performance using clinical labels as reference categories. Reported values represent averages across repeated cross-validation trials (Tab H-J one trial, otherwise 100 trials), and degrees of trustworthiness are used descriptively as post-hoc strata.

**Table D** **Average contingency table in L3 for the probable and confident trustworthiness degrees.**

| **Diagnosis**  **\FlowXAI** | **NC** | **CLL-like** | **MCL** | **FL** | **MZL** | **LPL** | **HCL** | **No** | **Percent** |
| --- | --- | --- | --- | --- | --- | --- | --- | --- | --- |
| **NC** | 55.7 | 1 | 0.1 | 0.5 | 1.1 | 1.1 | 0.1 | 2081 | 59.6 |
| **CLL-like** | 0.2 | 31.4 | 0.6 | 0 | 0.4 | 0.3 | 0 | 1149 | 32.9 |
| **MCL** | 0 | 0.3 | 0.4 | 0 | 0.1 | 0 | 0 | 28 | 0.8 |
| **FL** | 0 | 0 | 0 | 0.2 | 0 | 0 | 0 | 7 | 0.2 |
| **MZL** | 0.2 | 0.3 | 0.1 | 0.1 | 2.3 | 0.7 | 0 | 129 | 3.7 |
| **LPL** | 0.3 | 0.1 | 0 | 0.1 | 0.4 | 0.6 | 0 | 52 | 1.5 |
| **HCL** | 0.1 | 0 | 0 | 0 | 0.1 | 0 | 1 | 42 | 1.2 |
| **No** | 1973 | 1156 | 42 | 31 | 154 | 94 | 38 | 3488 | 99.9 |
| **Percent** | 56.2 | 33.1 | 1.2 | 0.9 | 4.4 | 2.7 | 1.1 | 99.9 | 85.1% |

The average FlowXAI performance for all test cases with either a *confident* or *probable* degree of trustworthiness reached an MCC of 85.1%. The columns depict the diagnoses according to the MLL9 dataset, and the rows present the predictions given by FlowXAI. The sum of the cross-validation trials was taken per table entry and then normalized to the sum of all entries. For the case numbers, the percentages per entry were multiplied by the average number of cases. (Corresponding to the red dot in Fig 3e).

**Table E** **Average contingency table in L3 for the confident trustworthiness degree.**

| **Diagnosis**  **\FlowXAI** | **NC** | **CLL-like** | **MCL** | **FL** | **MZL** | **LPL** | **HCL** | **No** | **Percent** |  |
| --- | --- | --- | --- | --- | --- | --- | --- | --- | --- | --- |
| **NC** | 56.4 | 0.7 | 0.1 | 0.3 | 0.8 | 0.8 | 0 | 1362 | 59.1 |  |
| **CLL-like** | 0.1 | 35.1 | 0.5 | 0 | 0.4 | 0.2 | 0 | 836 | 36.3 |  |
| **MCL** | 0 | 0.2 | 0.3 | 0 | 0 | 0 | 0 | 12 | 0.5 |  |
| **FL** | 0 | 0 | 0 | 0.1 | 0 | 0 | 0 | 2 | 0.1 |  |
| **MZL** | 0.1 | 0.3 | 0.1 | 0.1 | 1.7 | 0.5 | 0 | 65 | 2.8 |  |
| **LPL** | 0.1 | 0.1 | 0 | 0.1 | 0.2 | 0.3 | 0 | 18 | 0.8 |  |
| **HCL** | 0 | 0 | 0 | 0 | 0 | 0 | 0.3 | 7 | 0.3 |  |
| **No** | 1307 | 839 | 23 | 14 | 71 | 41 | 7 | 2302 | 99.9 |  |
| **Percent** | 56.7 | 36.4 | 1 | 0.6 | 3.1 | 1.8 | 0.3 | 99.9 | 89.3% |  |

The average FlowXAI performance in percent for all test cases with a confident degree of trustworthiness is shown in Fig 3d, with an average MCC of 89.3%. The columns depict the diagnoses according to the MLL9 dataset, and the rows present the predictions given by FlowXAI. For percentages, the sum of the cross-validation trials was taken per table entry and then normalized to the sum of all entries.

**Table F** **Average contingency table in L3 for the probable class.**

| **Diagnosis**  **\FlowXAI** | **NC** | **CLL-like** | **MCL** | **FL** | **MZL** | **LPL** | **HCL** | **No** | **Percent** |
| --- | --- | --- | --- | --- | --- | --- | --- | --- | --- |
| **NC** | 54.2 | 1.5 | 0.2 | 0.8 | 1.7 | 1.7 | 0.1 | 715 | 60.2 |
| **CLL-like** | 0.4 | 24.2 | 0.8 | 0.1 | 0.5 | 0.4 | 0 | 313 | 26.4 |
| **MCL** | 0 | 0.4 | 0.6 | 0 | 0.1 | 0.1 | 0 | 14 | 1.2 |
| **FL** | 0 | 0 | 0 | 0.3 | 0 | 0 | 0 | 4 | 0.3 |
| **MZL** | 0.5 | 0.4 | 0.1 | 0.2 | 3.5 | 1.1 | 0 | 69 | 5.8 |
| **LPL** | 0.5 | 0.2 | 0.1 | 0.2 | 0.8 | 1.3 | 0 | 37 | 3.1 |
| **HCL** | 0.3 | 0.1 | 0 | 0 | 0.3 | 0.1 | 2.2 | 36 | 3 |
| **No** | 664 | 318 | 21 | 19 | 82 | 56 | 27 | 1186 | 100 |
| **Percent** | 55.9 | 26.8 | 1.8 | 1.6 | 6.9 | 4.7 | 2.3 | 100 | 76.9% |
|  |  |  |  |  |  |  |  |  |  |

The average FlowXAI performance in percent for all test cases with a probable degree of trustworthiness using an 80/20 split is shown in Fig 3d, with an average MCC of 76.9%. The columns depict the diagnoses according to the MLL9 dataset, and the rows present the predictions given by FlowXAI. For percentages, the sum of the cross-validation trials was taken per table entry and then normalized to the sum of all entries.

**Table G** **Average contingency table in percent in L3 for the challenging trustworthiness degree.**

| **Diagnosis**  **\FlowXAI** | **NC** | **CLL-like** | **MCL** | **FL** | **MZL** | **LPL** | **HCL** | **No** | **Percent** |
| --- | --- | --- | --- | --- | --- | --- | --- | --- | --- |
| **NC** | 33.1 | 1.9 | 0.5 | 1.1 | 2.7 | 2.3 | 0.1 | 165 | 41.7 |
| **CLL-like** | 2.2 | 25.3 | 1.6 | 0.2 | 0.9 | 0.9 | 0 | 123 | 31.1 |
| **MCL** | 0.3 | 0.8 | 1.3 | 0 | 0.1 | 0.2 | 0 | 11 | 2.7 |
| **FL** | 0.3 | 0.1 | 0 | 0.7 | 0.1 | 0.2 | 0 | 6 | 1.4 |
| **MZL** | 2.6 | 0.7 | 0.3 | 0.5 | 7.4 | 2.6 | 0.1 | 56 | 14.2 |
| **LPL** | 1.8 | 0.5 | 0.3 | 0.7 | 2 | 3.9 | 0 | 36 | 9.2 |
| **HCL** | 0 | 0 | 0 | 0 | 0 | 0 | 0 | 0 | 0 |
| **No** | 160 | 116 | 16 | 13 | 52 | 40 | 1 | 395 | 100.3 |
| **Percent** | 40.3 | 29.3 | 4 | 3.2 | 13.2 | 10.1 | 0.2 | 100.3 | 60.1% |

The average FlowXAI performance in percent for all test cases with *challenging* degrees of trustworthiness using an 80/20 split is shown in Fig 3d, with an average MCC of 60.1%. The columns depict the diagnoses according to the MLL9 dataset, and the rows present the predictions given by FlowXAI. For percentages, the sum of the cross-validation trials was taken per table entry and then normalized to the sum of all entries.

**Table H** **Contingency table for 512 training cases in L3 for the confident trustworthiness degree.**

| **Diagnosis**  **\FlowXAI** | **NC** | **CLL-like** | **MCL** | **FL** | **MZL** | **LPL** | **HCL** | **No** | **Percent** |
| --- | --- | --- | --- | --- | --- | --- | --- | --- | --- |
| **NC** | 49.64 | 0.61 | 0.03 | 0.14 | 0.56 | 0.31 | 0.08 | 3818 | 49.69 |
| **CLL-like** | 0 | 39.76 | 0.29 | 0.04 | 0.55 | 0.18 | 0.03 | 3262 | 42.45 |
| **MCL** | 0 | 1.11 | 0.52 | 0.01 | 0.21 | 0.05 | 0 | 82 | 1.07 |
| **FL** | 0.01 | 0.52 | 0.16 | 0.3 | 0.85 | 0.42 | 0.01 | 48 | 0.62 |
| **MZL** | 0.01 | 0.34 | 0.03 | 0.08 | 1.08 | 0.38 | 0.04 | 281 | 3.66 |
| **LPL** | 0.03 | 0.1 | 0.05 | 0.05 | 0.35 | 0.16 | 0 | 115 | 1.5 |
| **HCL** | 0 | 0.01 | 0 | 0 | 0.07 | 0 | 0.86 | 78 | 1.02 |
| **No** | 3947 | 3138 | 146 | 174 | 150 | 57 | 72 | 7684 | 100.0 |
| **Percent** | 51.37 | 40.84 | 1.90 | 2.26 | 1.95 | 0.74 | 0.94 | 100.0 | 86.6% |

The FlowXAI performance in percent trained with 512 cases, and for all test cases judged as *probable* by Flow XAI; the MCC was 86.6%. The columns depict the diagnosis, and the rows present the predictions made by FlowXAI. It covers 49% (N=7684) of the whole test data. The third level of diagnosis (consisting of additional B-NHL diagnoses 3, 4, 5, and 6) has to diagnosed by human experts based on additional data (morphology, molecular biology).

**Table I** **Contingency table for 512 training cases in L3 for the probable trustworthiness degree.**

| **Diagnosis**  **\FlowXAI** | **NC** | **CLL-like** | **MCL** | **FL** | **MZL** | **LPL** | **HCL** | **No** | **Percent** |
| --- | --- | --- | --- | --- | --- | --- | --- | --- | --- |
| **NC** | 53.36 | 1.47 | 0.17 | 0.33 | 1.65 | 1.14 | 0.21 | 3099 | 63.65 |
| **CLL-like** | 0.06 | 31.44 | 0.26 | 0.03 | 0.43 | 0.15 | 0.02 | 1111 | 22.82 |
| **MCL** | 0.18 | 1.02 | 0.43 | 0.02 | 0.17 | 0.05 | 0 | 60 | 1.23 |
| **FL** | 0.56 | 0.45 | 0.16 | 0.31 | 0.79 | 0.46 | 0.01 | 52 | 1.07 |
| **MZL** | 0.46 | 0.34 | 0.04 | 0.06 | 0.97 | 0.35 | 0.02 | 283 | 5.81 |
| **LPL** | 0.34 | 0.11 | 0.07 | 0.06 | 0.4 | 0.38 | 0 | 205 | 4.21 |
| **HCL** | 0.14 | 0.01 | 0 | 0 | 0.09 | 0.02 | 0.84 | 59 | 1.21 |
| **No** | 3373 | 929 | 87 | 169 | 132 | 114 | 65 | 4869 | 100.0 |
| **Percent** | 63.65 | 22.82 | 1.23 | 1.07 | 5.81 | 4.21 | 1.21 | 100.0 | 62.4% |

The FlowXAI performance in percent, trained with 512 samples, for all test samples was judged as *probable* by FlowXAI*,* resulting in an MCC of 62.4%. It covers 30% (N=4869) of the test data. The columns depict the physician’s manual diagnosis according to [1], and the rows show the predictions made by FlowXAI. The percentages of correct diagnoses of the L3 lymphoma entities are highlighted in green, and the MCC is marked in blue.

**Table J** **Contingency table for 512 training cases in L3 for the challenging trustworthiness degree.**

| **Diagnosis**  **\FlowXAI** | **NC** | **CLL-like** | **MCL** | **FL** | **MZL** | **LPL** | **HCL** | **No** | **Percent** |
| --- | --- | --- | --- | --- | --- | --- | --- | --- | --- |
| **NC** | 45.43 | 12.14 | 1.43 | 1.47 | 7.96 | 5.24 | 0.59 | 2025 | 74.26 |
| **CLL-like** | 0.59 | 7 | 0.07 | 0 | 0.11 | 0.15 | 0 | 216 | 7.92 |
| **MCL** | 0.92 | 0.48 | 0.51 | 0.07 | 0.26 | 0.22 | 0 | 67 | 2.46 |
| **FL** | 3.37 | 0.18 | 0.18 | 0.55 | 0.4 | 0.44 | 0 | 140 | 5.12 |
| **MZL** | 2.31 | 0.33 | 0.07 | 0 | 0.84 | 0.51 | 0 | 111 | 4.06 |
| **LPL** | 2.27 | 0.22 | 0.07 | 0.22 | 0.33 | 0.88 | 0 | 109 | 3.99 |
| **HCL** | 0.84 | 0.04 | 0 | 0.04 | 0.15 | 0.04 | 1.06 | 59 | 2.17 |
| **No** | 1520 | 556 | 64 | 64 | 274 | 204 | 45 | 2727 | 100.0 |
| **Percent** | 55.73 | 20.39 | 2.33 | 2.35 | 10.05 | 7.48 | 1.65 | 100.0 | 23.6% |

The FlowXAI performance in percent, trained with 512 samples, for all test samples was judged as *challenging* by FlowXAI*,* resulting in an MCC of 23.6%. It covers 21% (N=2727) of the test data. The columns depict the physician’s manual diagnosis according to[1], and the rows show the predictions made by FlowXAI. The percentages of correct diagnoses of the L3 lymphoma entities are highlighted in green, and the MCC is marked in blue.

**Fig A.** **Exemplary decision logic of the ALPODS expert committee for the PUM2 dataset.**


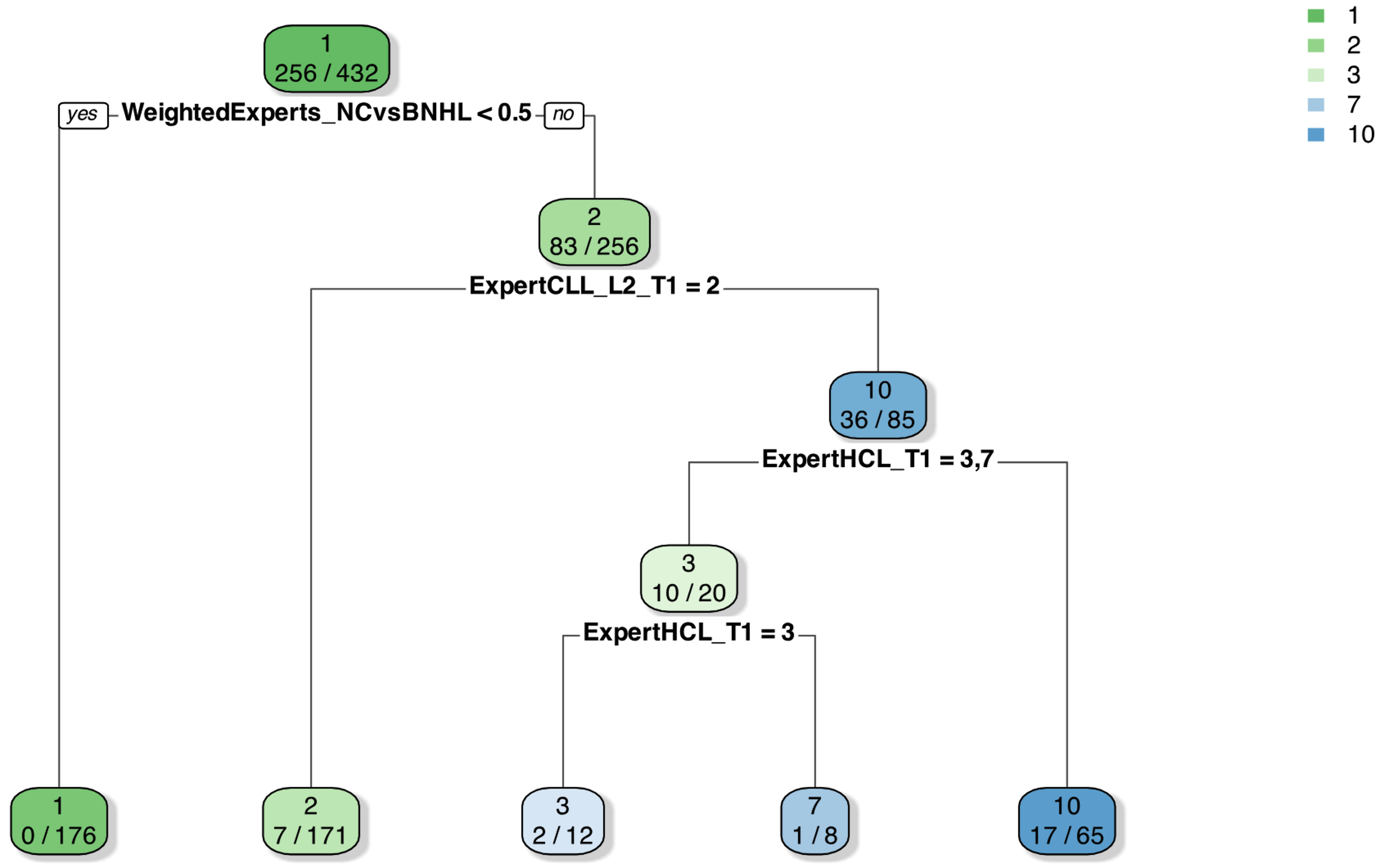


For the exemplary decision logic of the ALPODS expert committee it should be noted, that we did not meet minimal typical learning sample requirements for HCL or any other level 3 lymphoma (see SI table 1) within the PUM2 dataset. Explicit marker thresholds and clearly defined decision paths are detailed in a digital attachment PUM2_DecisionLogic.zip.

Abbreviations 1= normal controls, 2= CLL-like, 3=MCL, 7=HCL, 10=all other lymphoma

**Text A. ALPODS expert committee, Tile Mining-based sample selection, and model construction.**

Building on the curated sample set, the supervised ALPODS expert committee—provides self-explanation through a structured decision framework that integrates medical knowledge with data-driven population analysis.

For each expert, a recursive process constructs a directed acyclic graph (DAG). At each node, a marker is selected using the Simpson index [2], which quantifies population homogeneity with respect to the task-specific labels defined at the corresponding diagnostic level. These labels are not global clinical labels but are internally defined per diagnostic level, reflecting medically meaningful decision stages specified by domain experts. Conditional relationships between parent and child nodes are then derived in a Bayesian-inspired manner, yielding score-based quantities that reflect relative likelihoods. Recursion terminates when further splitting produces subpopulations with homogeneous internal labels or when population sizes fall below a predefined threshold.

The resulting DAGs identify phenotypically relevant cell subpopulations. For explanatory purposes, these structures are distilled into fast-and-frugal trees [3] by merging decisions on identical markers into range-based conditions and ranking resulting subgroups according to effect sizes measured by Cohen’s d [4].

To capture the hierarchical nature of hematologic diagnostics, multiple tube- and task-specific ALPODS experts are trained at three diagnostic levels and aggregated into an expert committee. At level 1 (distinguishing normal controls from B-cell lymphomas), Tile Mining first selects representative cases per class. Computed ABC analysis [5] is then applied to partition learned cell populations into high-frequency (set A) and low-frequency (sets B and C) groups, each analyzed by a dedicated expert. Depending on the dataset, four to six experts (one per group per tube) are trained, with inter-tube weighting reflecting differential diagnostic relevance.

At levels 2 and 3, additional ALPODS experts are trained to resolve progressively finer diagnostic distinctions. Three experts distinguish CLL-like entities from other lymphomas across all tubes, while a dedicated expert analyzes the most informative tube for hairy-cell leukemia. Each ALPODS expert independently produces a task-specific decision score and an associated explanation. These outputs are aggregated by a higher-order meta-classifier implemented as a standard decision tree using the Gini impurity measure for categorical data. The meta-classifier is trained separately within each cross-validation split and combines expert opinions using a weighting scheme that reflects diagnostic relevance at each level and for each tube. Further details are provided in Figs 6 and Fig A.

TM is used to identify the curated sample set. TM identifies typical cases, from which at minimum 32 cases per diagnosis (512) serve to define representatives per diagnosis. In the first step, ALPODS experts learn the relevant populations for the specific task described below and store the frequencies within tables. This means that each ALPODS model is an expert that answers a specific question for a particular tube of a patient sample. All ALPODS experts learn populations for each tube separately for a specific task. The L1 task distinguishes whether the sample is an NC or B-cell lymphoma.

After the populations are identified, they are divided into two groups per tube using computed ABC analysis [5]. The ABC curve of the computed ABC analysis can be used to visualize the data by graphically representing the cumulative distribution function closely related to the Lorenz curve. Using the ABC curve, the algorithm calculates optimal limits by exploiting mathematical properties pertaining to the distribution of the analyzed populations. The first group of populations has large frequencies (set A in the computed ABC analysis) and is analyzed by the first ALPODS expert. The second group of populations is identified as sets B and C within the algorithm. Populations in the second group have smaller frequencies and are used by the second ALPODS expert. Both ALPODS experts learn to distinguish the NC from B-cell lymphoma. In sum, six (four) experts are employed for the L1 task since the MLL9F dataset (PUM2 dataset) consists of three (two) tubes. Here, expert opinions on samples from tube two are weighted more than those from tube one, and those from tube one are weighted more than those from tube three by the ALPODS experts for the L1 task. Four experts are used for the PUM2 dataset, which incorporates only two tubes.

In case of L2 and L3, more ALPODS experts are trained on the populations extracted for specific tasks. Three experts investigated the three tubes for CLL-like lymphoma, while three other ALPODS experts investigated the tubes for other types of lymphoma. One ALPODS expert determines whether a patient has HCL, using samples from tube two, the samples from other tubes not being useful to human expert when diagnosing HCL. In summary, ALPODS experts at specific levels (L1, L2 and L3) diagnose the samples context-specific and separately. Their opinions are combined to an AI committee to form a mixture of experts. The opinion of the full committee that provides the final diagnosis is generated by a decision tree using an impurity function that is applicable to categorical data [6].

**Fig B. Illustration of outliner samples with dot plots.**


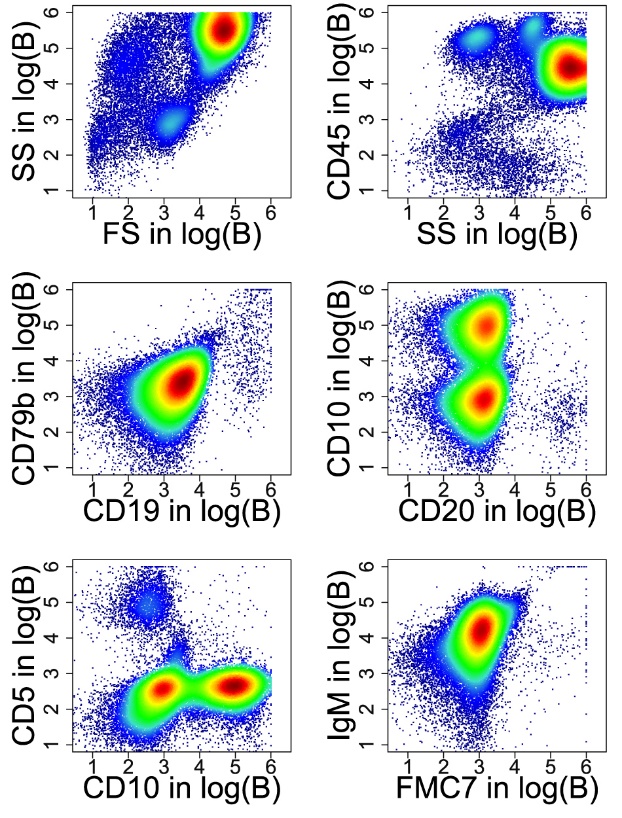

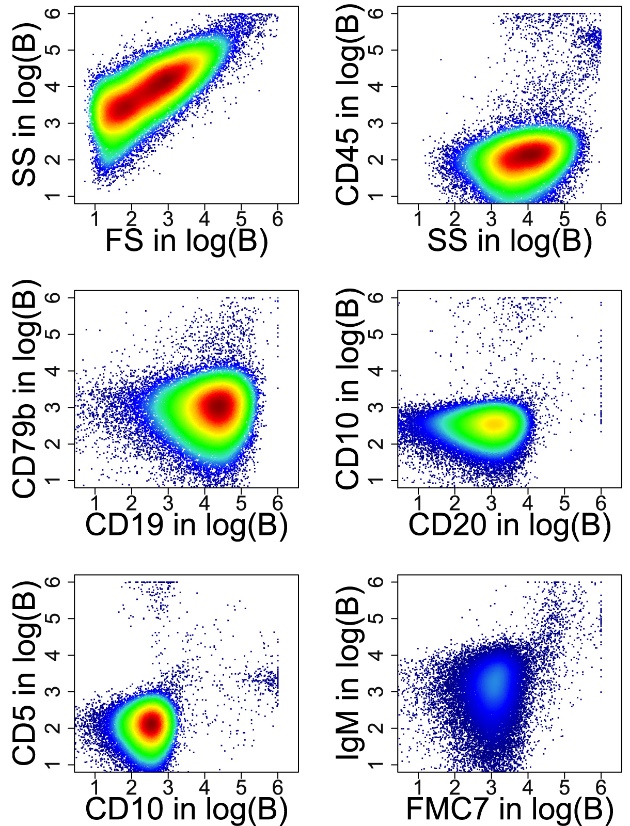


Quality control of sample files identified with TM revealed that 19% of the samples within the MLL9F sample files were atypical. Manual validation was performed on 100 samples hereby confirming that TM reliably distinguished structurally typical from atypical samples. All 100 evaluated samples are available as fcs files at 10.5281/zenodo.19681022. Tube 1 of a typical three-tube panel for a typical patient (A; left) and an atypical patient (B; right) are presented here in log scale. Human experts assume that red blood cells are predominant in atypical samples for the reason that most recorded events do not stain positive for CD45 or other leukocyte antigens. Other presumed reasons for the strong alterations were incomplete erythrocyte lysis, irregular antibody staining patterns or other aberrations, most likely acquisition artifacts.

**Fig C.** **Explaining FlowXAI diagnoses decisions.**


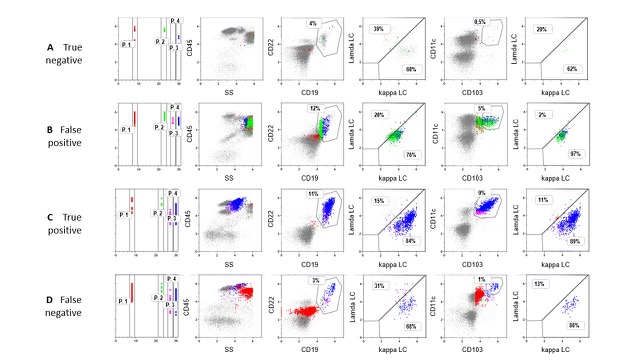


Four HCL samples (rows A-D) were analyzed manually via FlowXAI extracted FCS files using commercial (Kaluza™) software (version 2.1) in log scale. The classification of the samples is as follows: a: true negative, b: false positive, c: true positive, d: false negative. The populations selected by Flow XAI as relevant for its decisions are highlighted in the first column (P1-P4) and colored in all diagrams. The conventional manual gating procedure involved lymphocyte versus monocyte and granulocyte imaging (second column), B-cell imaging (third column), light chain distribution on B cells (fourth column), depiction of hairy cells (fifth column) and assessment of light chain distributions on hairy cells (sixth column). For simplification, not all typical hairy cell epitopes are depicted.

**Text B.** FlowXAI explainability.

FlowXAI justifies its diagnostic decisions by providing case-specific explanations that are interpretable by physicians [7]. For example, the diagnosis of HCL is supported when FlowXAI identifies small but characteristic B-cell populations with antigen expression patterns consistent with clinical diagnostic criteria (e.g., characterized as CD103+++, CD25+++, CD19++, CD25++, or SS++). For the confident trustworthiness degree, on average, all HCL cases were classified correctly. The false-positive (N=4) and false-negative HCL (N=4) cases were, on average, assigned to the probable degree of trustworthiness. FlowXAI learned and subsequently used four particular cell populations to classify the cases as HCL. The decision depended solely on tube 2, containing the relevant HCL antibodies. These four populations, considered to be highly relevant to FlowXAI’s diagnostic decision-making, are highlighted in the left column (SI Fig C). In the first row, an NC case (named a true negative) is depicted; the B-cells show a typical light chain distribution pattern for kappa and lambda, and the “hairy cell gate” (CD103 vs. CD11c) is almost empty (SI Fig C,a). In the second row, HCL was incorrectly suspected because ALPODS revealed populations in the B-cell or hairy cell regions (SI Fig C, b). A human expert can recognize that CD19+ cells do not appear as lymphocytes in CD45+ vs. SS images and “understands” that FlowXAI’s reasoning is incorrect. Sample degradation could be a biological reason for granulocytes' presumably unselective antibody capture. A real HCL case is depicted as a “true positive” in the third row (SI Fig C,c), which illustrates that the hairy cells correspond to “highly scattered lymphocytes” in the CD45 vs. SS image and are indeed CD103++CD11c+++, i.e., they highly express these epitopes. Additionally, as depicted in the right column, the correctly classified hairy cells exhibited light chain restrictions, i.e., a significant predominance of kappa vs. lambda light chains. CD25 and other B-cell epitope expression patterns are not shown to simplify the graphic. Finally, a false negative sample was chosen for further illustration (SI Fig C.d). Although the algorithm correctly recognized that the events in the hairy cell region were not B cells, the subtle population masked by the irregular staining pattern of a dominant granulocytic fraction was not detected. Of note, there were fewer than 700 relevant events in each population. Dot plot images for the remaining six incorrectly classified HCL cases are provided in Figs D and E.

**Fig D. Explanations for HCL misdiagnosis using bivariate dot plots in log scale: Three false positive results for distinguishing HCL patients from NCs illustrating how FlowXAI explanations allow retrospective clinical interpretation.**


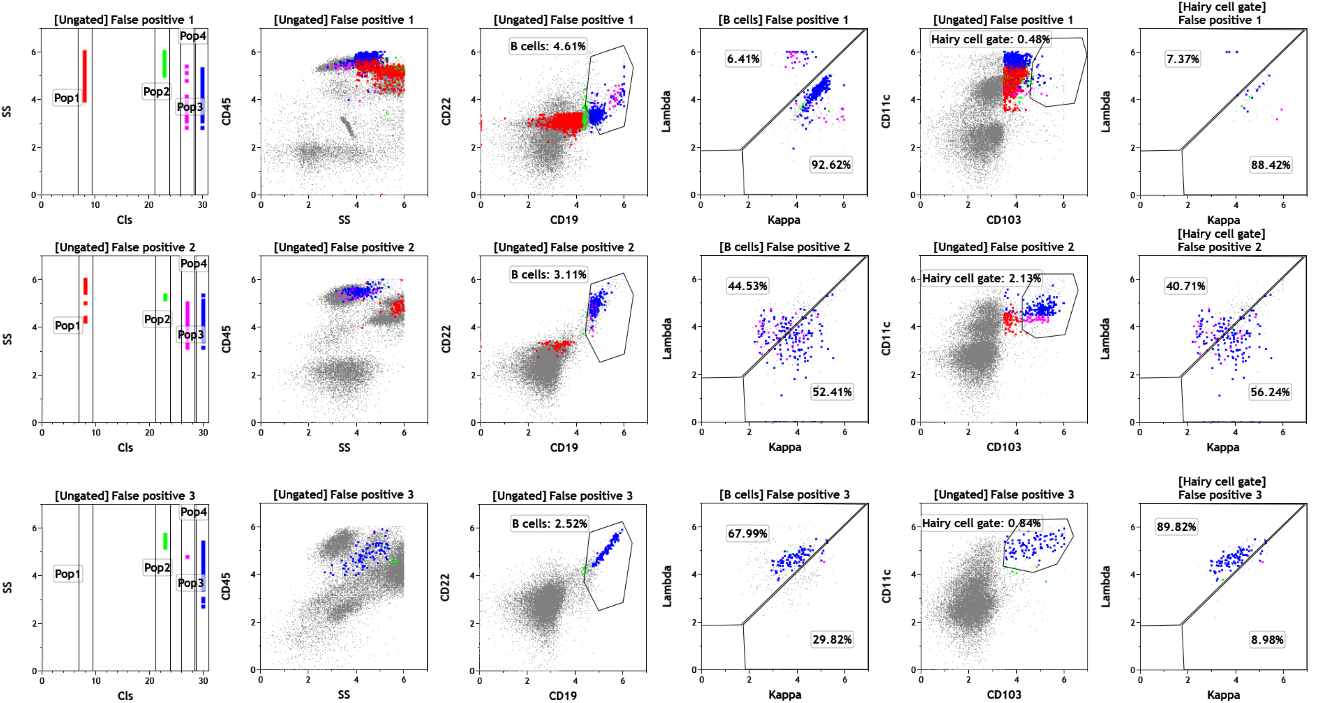


**Fig E. Explanations for HCL misdiagnosis using bivariate dot plots in log scale: Three false negative results for distinguishing HCL patients from NCs.**


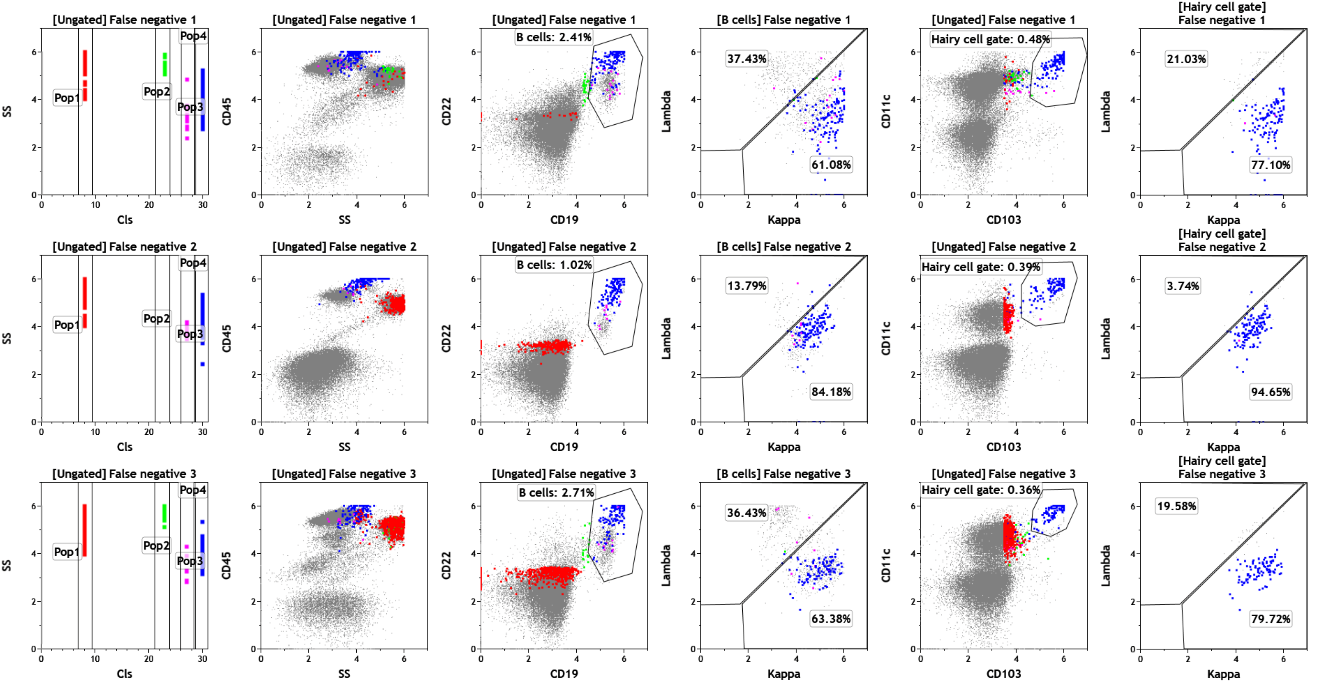


**Fig F. FlowXAI-based lymphoma classification results for atypical cases in the PUM2 dataset.**


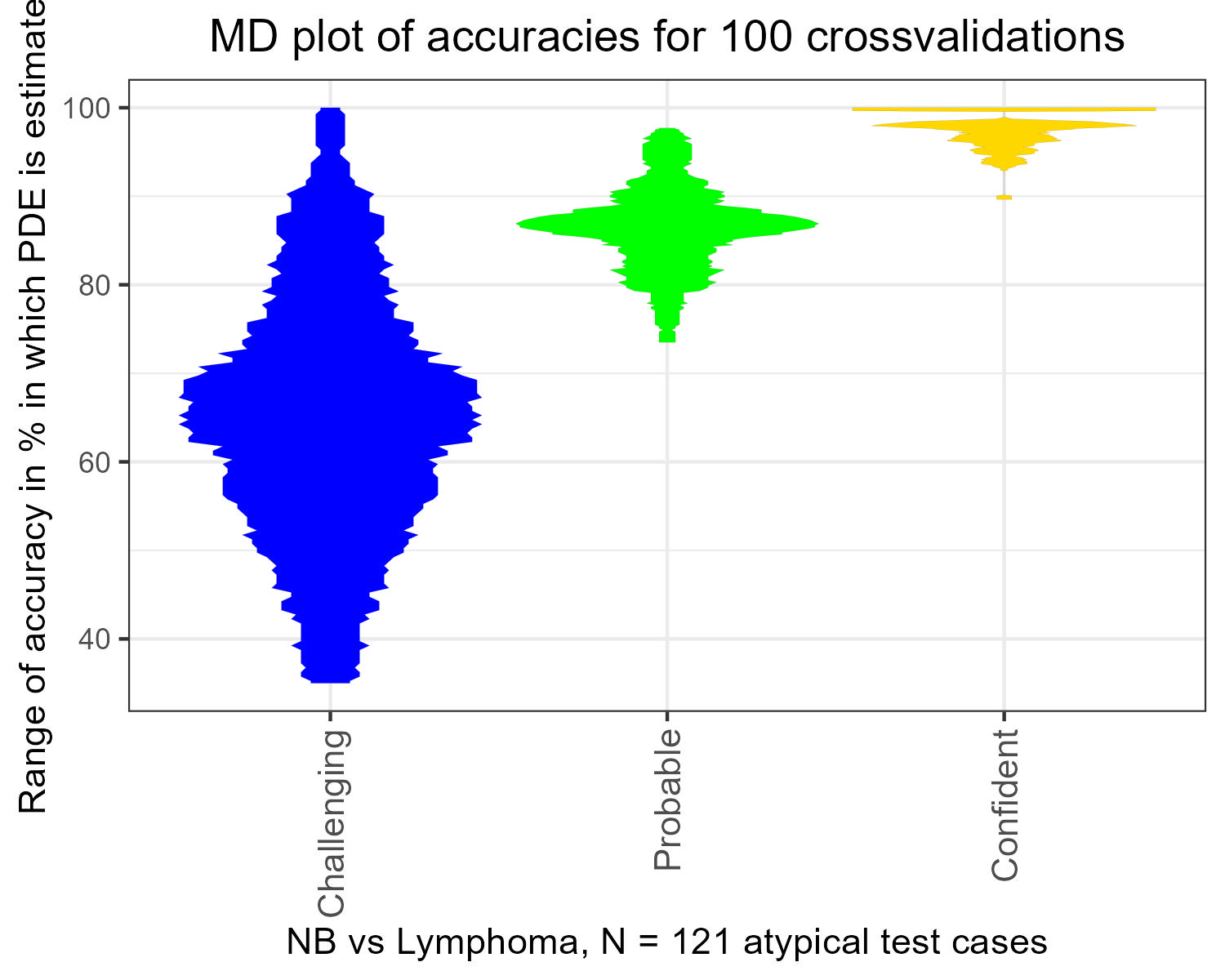

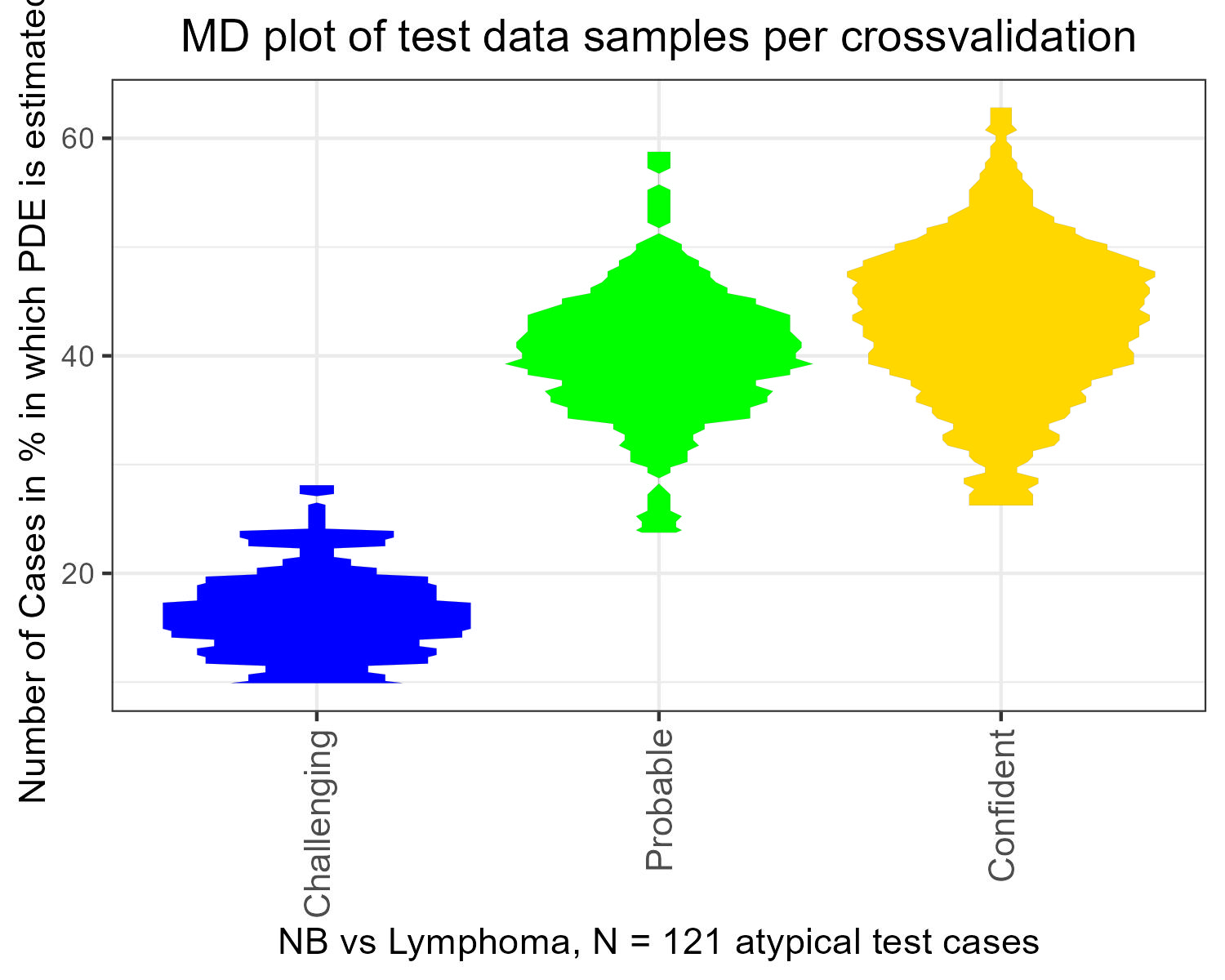


One hundred cross-validation steps were visualized with an MD-plot for the three degrees of trustworthiness. MD-plots were used to estimate and visualize the probability density function. The absence of Gaussian distributions in the estimated probability density functions suggests increased heterogeneity in this independent dataset. This observation likely reflects both limited training sample size and panel-specific differences, rather than failure of the diagnostic framework.

**Text C. Atypical cases in the PUM2 dataset.**

To validate the applicability of FlowXAI to diverse B-NHL immunophenotyping datasets, we utilized a second dataset comprising 638 samples obtained from the diagnostic center Marburg (PUM2). This diagnostic laboratory employs a panel targeting distinct B-cell epitopes, measured across two tubes samples per case/patient, as previously described[8].

Initially, the TM algorithm was used to distinguish typical from atypical cases. Subsequently, 100 cross-validation trials were conducted on L1 cases. All training cases were selected exclusively from the typical category. For each trial, the training dataset consisted of 100 normal controls (NC) and 100 B-NHL cases, distributed as follows: 30 CLL, 15 MCL, 10 FL, 10 MZL, 5 LPL, 10 HCL, 10 MBL, and 10 NS.

The evaluation results for the typical cases in the test sets are detailed in the main text. A total of 121 test cases were considered as atypical in SI Fig F. In Fig F, left, the Accuracy metric was selected to account for balanced group sizes. The accuracy was 99% when FlowXAI was confident, 89% when XAI estimated that the appropriate diagnosis was probable, and 69% when XAI judged a case as challenging (SI, Fig F, left). The variance in the confident degree of trustworthiness is very low, while the challenging degree exhibits a high variance, as shown in the MD plot.

Despite the relatively small training set size, FlowXAI classified 57% of cases as confident, 31% as probable, and 12% as challenging (SI, Fig F, right). Interestingly, none of the estimated PDFs exhibited a Gaussian distribution (magenta frame). This observation suggests that learning structural patterns in the data could be improved with a larger dataset.

**Text D. Literature evaluation for seeking benchmark algorithms.**

A comprehensive literature screening was conducted to identify relevant sources about cell population gating in flow cytometry. This evaluation was conducted to contextualize FlowXAI relative to existing open-source approaches rather than to establish a definitive benchmark ranking. Special attention was given to seminal studies, recent advancements, and established gating methodologies. While the primary focus of the selection of gating algorithms, widely utilized gating software, including FlowJo, FCS Express, WinMDI, CytoPaint, Leukobyte, VenturiOne, CellQuest Pro, and Cytospec, was acknowledged. However, the primary investigation did not extend to these proprietary gating software programs. To select a subset of algorithms, a systematic search was performed on Google Scholar using the keywords "gating" and "flow cytometry" to identify relevant publications and research in the field. Citations from the identified publications were meticulously followed to ensure a comprehensive understanding of the current landscape and recent advancements in gating methodologies.

In addition, algorithms used in the FlowCap challenges were thoroughly examined. These challenges provided valuable insights into the evaluation of gating algorithms and their performance across various datasets, contributing to a robust assessment of the efficacy and applicability of the proposed gating algorithm. A relevance assessment was conducted to ascertain the significance and impact of the identified literature by normalizing citations through the number of years until 2023. This approach facilitated a comprehensive evaluation of each publication's enduring contributions and relevance within the context of cell population gating in contemporary flow cytometry research. In the next step, only publications referencing open-source code in R or Python were considered.

On the remaining publications, a recursive ABC analysis [5] using the R package “ABCanalysis” available on CRAN (<http://cran.r-project.org/web/packages/ABCanalysis/index.html>) was applied to prioritize key publications and gating methodologies. The ABC curve can visualize the data by graphically representing the cumulative distribution function closely related to the Lorenz curve. Using the ABC curve, the algorithm calculates the optimal limits by exploiting the mathematical properties of the distribution of analyzed items. This method allows for systematical identification of the most effective and innovative gating techniques based on their normalizing citations, which then can be identified in group A. In analogy to [9], ABC analysis was applied recursively for group A until the eight most influential publications could be identified.

The investigation revealed that a significant proportion, approximately 70% of 95 algorithms in 68 publications of the identified algorithms, employed unsupervised techniques. The analysis of auto-gating publications delineated distinct "waves" of prominent research activity, notably in the years 2001, 2009/2010, and 2019. These pivotal periods marked heightened interest in developing and applying automated gating methodologies within the field of flow cytometry. Next, recursive ABCanalysis on normalized citations was employed. Consequently, among the identified algorithms, eight were deemed highly relevant by recursive ABCanalysis, with the added advantage of being open-source software. Notable algorithms included PhenoGraph, Citrus, FlowSOM, CellCNN, diffCyt, FLAME, FlowPeak, and FlowClust, each presenting unique features and approaches to streamline and enhance the accuracy of automated gating procedures. Diffcyt does not provide the necessary functionality. Citrus and CellCNN mention the required capabilities, but in their open-source code, they need a class per sample file and return a class per sample file, which did not fit the problem structure we defined. FlowSOM, PhenoGraph, FlowClust, and FlowPeak were tried out, but we could not generate a sufficient B-cell gate on the two given datasets.

**Text E. Benchmarking the FlowXAI Algorithm with CITRUS.**

Benchmarking with CITRUS was performed to assess whether pre-diagnostic selection of structurally typical samples by TM improves performance of an established population-based algorithm. Both CITRUS and FlowXAI were restricted to a single tube to ensure methodological comparability. Differences in accuracy distributions were assessed descriptively and statistically, acknowledging that algorithmic objectives and internal representations differ substantially. 512 samples were randomly selected for training, with 256 NC samples and 256 B-NHL samples equally distributed for diagnoses (see SI Table 3) were randomly drawn 100 times. The samples were either drawn randomly or restricted to the category of typical samples as defined by TM. For simplicity, both AI systems CITRUS and FlowXAI, as the committee of ALPODS experts, were restricted in this experiment to a single tube. Fig 4B shows the accuracies of the pdfs based on 96.7% (n=15,271) of the test cases in the MD plots. The L1 performance of TM-CITRUS was computed to test whether TM selection of typical training cases improves the CITRUS algorithms' usage. The median accuracies for the CITRUS, TM-FlowXAI, and TM-CITRUS models were, on average, 82%, 90%, and 89%, respectively, although it should be noted that all three distributions were skewed and not normally distributed. The differences of accuracies were tested with an unpaired two-sided Wilcoxon rank sum tests with continuity $p(BasicCitrus,TM$ correction with the alternative hypothesis being that the true location shift is not equal to zero, $-Citrus)<2e-16, W=1240, N=100$, and p$(FlowXAI,TM-Citrus)<2e-16, W=8414, N=100$.

**Text F. Selective one-vs-rest ROC and precision-recall analysis from FlowXAI categories of trustworthiness.**

We generated selective one-vs-rest ROC and PR curves using the FlowXAI trustworthiness categories: confident, probable and challenging in SI Figs G and H. All held-out predictions the 100 cross validation trials were pooled. For each diagnosis, the true class label was recoded into a one-vs-rest binary endpoint, and the trustworthiness categories were treated as an ordered three-level selection variable. Because the trustworthiness mapping defines clinically meaningful categories rather than a calibrated probability scale, the selective analysis was based on nested category thresholds: confident only, probable or better, and challenging or better (= all test data predictions for that diagnosis). The ROC curves in SI Fig G summarize the trade-off between sensitivity and false-positive rate across thresholds, whereas PR curves in SI Fig H summarize the trade-off between precision and recall. In imbalanced diagnostic settings, PR curves are often more informative than ROC curves because they directly quantify the positive predictive utility of model calls at different acceptance thresholds [10]. We therefore report both curve types, with AUC summarizing selective ROC performance and AP summarizing selective PR performance.

The selective curves showed marked entity-specific differences. NC and CLL-like displayed the strongest selective discrimination, with AUC values of 0.953 and 0.961 and AP values of 0.937 and 0.925, respectively. HCL also showed strong selective utility despite its low prevalence, with AUC 0.935 and AP 0.685. MZL showed intermediate performance (AUC 0.756, AP 0.355). In contrast, MCL, FL, and LPL showed limited selective utility, with AUC/AP values of 0.655/0.162, 0.601/0.152, and 0.632/0.136, respectively. Overall, these curves indicate that stricter trustworthiness category selection substantially enriches correctness for NC, CLL-like, and HCL, whereas for MCL, FL, and LPL stricter selection tends to reduce recall more than the gain in precision.

At the level of clinically interpretable operating points, NC and CLL-like retained high precision across all three thresholds while gaining recall as the selection rule was relaxed, indicating that the FlowXAI trustworthiness categories provide a useful ranking of prediction reliability for these entities. HCL showed a similar pattern, with a strong gain in recall when moving from confident only to probable or better, while maintaining high precision. For HCL, the probable and challenging operating points overlapped, indicating that inclusion of the challenging category did not add further HCL predictions in the pooled held-out data. By contrast, MCL, FL, and LPL showed only limited improvements in precision under stricter selection, suggesting that trustworthiness alone does not fully resolve the intrinsic overlap of these entities. MZL occupied an intermediate position, consistent with partial but not uniform clinical decision-support value.

Taken together, these selective ROC and PR analyses support a selective-use interpretation of FlowXAI. The trustworthiness categories appear clinically most useful for NC, CLL-like, and HCL, where high-trust predictions are strongly enriched for correctness. For more difficult entities, especially MCL, FL, and LPL, the curves support use as expert-support rather than stand-alone automated decision-making.

**

**

**Fig G. FlowXAI one-vs-rest selective ROC curves by diagnosis.**

Receiver operating characteristic curves were generated for each diagnosis in a one-vs-rest setting using the ordered FlowXAI trustworthiness degrees. The three labeled operating points correspond to **confident only, probable or better,** and **challenging or better** (= all predictions of the respective diagnosis). Curves were obtained after pooling all held-out predictions across matrix columns. The area under the ROC curve (AUC) is shown in each panel. ROC analysis summarizes the trade-off between true-positive rate and false-positive rate across thresholds [11].

**

**

**Fig H.** **FlowXAI one-vs-rest selective precision-recall curves by diagnosis.**

Precision-recall curves were generated for each diagnosis in a one-vs-rest setting using the same degrees of trustworthinessas in Supplementary Fig SG. The three labeled operating points correspond to **confident only, probable or better,** and **challenging or better** (= all predictions of the respective diagnosis). Curves were obtained after pooling all held-out predictions. Average precision (AP) is shown in each panel. Because the lymphoma classes are imbalanced, precision-recall curves are particularly informative for judging the positive predictive utility of selective acceptance rules [10].

**Text G. Conventional single-tube baselines based on flowFP fingerprints.**

To provide a conventional reference for MLL9F tube 1, we trained two standard sample-level classifiers on flowFP-derived fingerprints[12] using the same 100 repeated train–test splits as in the main cross-validation framework through a Mac Studio M4 max, 16Cores, 128 GB RAM. Within each split, a flowFP model was constructed on training samples only to avoid leakage. Training and test samples of that split were then fingerprinted with the same training-derived flowFP model, yielding fixed-length sample-level feature vectors suitable for conventional machine-learning models. The use of flowFP as a fingerprinting step follows the original flowFP framework for transforming flow-cytometry event data into fixed sample-level representations. In the baseline runs used here, the flowFP procedure used n_recursions = 7, fp_transform = "normalized", and computationally tractable subsampling settings (fp_model_max_samples = 100, fp_model_event_subsample = 500, fp_apply_event_subsample = 1000, chunk_size = 64). No class weighting was applied.

Two classifiers were trained on these fingerprints. First, a multinomial elastic-net logistic regression model was fitted using glmnet[13], with alpha_grid = {0, 0.5, 1}, inner 5-fold cross-validation, and lambda.1se as the model-selection rule. The final alpha value was selected by the smallest inner cross-validated classification error. Second, a random forest was trained using ranger[14] with 500 trees, and minimum node size 1 with all other parameters set by default. In both models, predictions were generated for all samples, but performance evaluation used only the held-out test samples of each outer split. Multiclass performance was summarized by Matthew’s correlation coefficient (MCC) computed on the test sets. The elastic-net fitting strategy follows the standard glmnet formulation for penalized multinomial generalized linear models, and the random-forest implementation follows the ranger reference implementation.

**Text H. Strangeness distributions and Tile Mining parameters.**

TM strangeness distributions were modeled separately for each tube using a robust Gaussian reference model, whose location (M) and standard deviation (SD) were estimated by a genetic algorithm. For the MLL9F dataset, the estimated parameters were M = 9.51, SD = 12.65 for tube 1, M = 9.39, SD = 13.38 for tube 2, and M = 9.03, SD = 14.19 for tube 3.

In the density panels, the empirical strangeness distribution is shown by Pareto density estimation (PDE), while the fitted robust Gaussian is overlaid in magenta in SI Figs I-J. The red vertical lines indicate the lower and upper TM thresholds. For MLL9F, these thresholds were −11.37 and 30.39 for tube 1, −16.75 and 35.55 for tube 2, and −12.97 and 31.04 for tube 3.

The corresponding quantile-quantile (QQ) plots compare the empirical strangeness values with the fitted Gaussian reference. Approximate linearity of the central quantiles indicates that the robust Gaussian provides a reasonable thresholding model for the bulk of the distribution, whereas deviations in the tails indicate departures from exact normality. Thus, the Gaussian should be interpreted here as a pragmatic reference model for TM thresholding, rather than as a strict assumption that the full empirical distribution is perfectly normal. SI Fig I shows these results for the three tubes of the MLL9F dataset. SI Fig J presents the corresponding analyses for the two tubes of the external PUM2 dataset.

Methodological details on Pareto density estimation (PDE) are described in [15, 16].

**Fig I. Tube-specific TM strangeness distributions and robust Gaussian thresholding for the MLL9F dataset.**

**

**

Left panels show the empirical distribution of TM strangeness values estimated by Pareto density estimation (PDE) for **tubes 1–3** of MLL9F. The fitted robust Gaussian reference model is shown in **magenta,** and the corresponding lower and upper TM thresholds are indicated by **red vertical lines.** Right panels show **quantile-quantile (QQ) plots** comparing the empirical strangeness values with the fitted Gaussian reference. Approximate linearity in the central range indicates that the Gaussian provides a reasonable reference for TM thresholding of the bulk distribution, while tail deviations reflect departures from exact normality.
**Abbreviations:** TM, Tile Mining; PDE, Pareto density estimation; QQ, quantile-quantile; SD, standard deviation.

**Fig J. Tube-specific TM strangeness distributions and robust Gaussian thresholding for the external PUM2 dataset.**

**

**

Left panels show the empirical distribution of TM strangeness values estimated by Pareto density estimation (PDE) for the **two PUM2 tubes**. The fitted robust Gaussian reference model is shown in **magenta**, and the corresponding lower and upper TM thresholds are indicated by **red vertical lines.** Right panels show **quantile-quantile (QQ) plots** comparing the empirical strangeness values with the fitted Gaussian reference. These plots illustrate how well the robust Gaussian captures the central part of the empirical strangeness distribution and support its use as a pragmatic thresholding model for TM in the external dataset.
**Abbreviations:** TM, Tile Mining; PDE, Pareto density estimation; QQ, quantile-quantile; SD, standard deviation.

Values underlying reported distributions, means, standard deviations, calibration summaries, ROC/precision-recall curves, figures, and related summary measures are provided in the Supporting Information and supplementary data files accessible via <https://doi.org/10.5281/zenodo.20554716>

**Supplementary References**

1. Rawstron AC, Kreuzer KA, Soosapilla A, Spacek M, Stehlikova O, Gambell P, et al. Reproducible diagnosis of chronic lymphocytic leukemia by flow cytometry: An European Research Initiative on CLL (ERIC) & European Society for Clinical Cell Analysis (ESCCA) Harmonisation project. Cytometry Part B: Clinical Cytometry. 2018;94(1):121-8.

2. Simpson EH. Measurement of Diversity. Nature. 1949;163(4148):688-. doi: 10.1038/163688a0.

3. Luan S, Schooler LJ, Gigerenzer G. A signal-detection analysis of fast-and-frugal trees. Psychological review. 2011;118(2):316.

4. Cohen J. Statistical power analysis for the behavioral sciences. New York: Academic Press; 2013.

5. Ultsch A, Lötsch J. Computed ABC Analysis for Rational Selection of Most Informative Variables in Multivariate Data. PloS one. 2015;10(6):e0129767. doi: 10.1371/journal.pone.0129767.

6. Breiman L, Friedman J, Stone CJ, Olshen RA. Classification and regression trees: CRC press; 1984.

7. Miller T, Howe P, Sonenberg L, AI E, editors. Explainable AI: Beware of inmates running the asylum. International Joint Conference on Artificial Intelligence, Workshop on Explainable AI (XAI); 2017. p. 36-42.

8. Hoffmann J, Rother M, Kaiser U, Thrun MC, Wilhelm C, Gruen A, et al. Determination of CD43 and CD200 surface expression improves accuracy of B-cell lymphoma immunophenotyping. Cytometry Part B: Clinical Cytometry. 2020;98(6):476-82. doi: 10.1002/cyto.b.21936.

9. Thrun MC, Mack E, Neubauer A, Haferlach T, Frech M, Ultsch A, et al. A Bioinformatics View on Acute Myeloid Leukemia Surface Molecules by Combined Bayesian and ABC Analysis. Bioengineering. 2022;9(11):642. doi: bioengineering9110642.

10. Saito T, Rehmsmeier M. The precision-recall plot is more informative than the ROC plot when evaluating binary classifiers on imbalanced datasets. PloS one. 2015;10(3):e0118432.

11. Fawcett T. An introduction to ROC analysis. Pattern recognition letters. 2006;27(8):861-74.

12. Rogers WT, Holyst HA. FlowFP: A bioconductor package for fingerprinting flow cytometric data. Advances in bioinformatics. 2009;(1):193947. doi: 10.1155/2009/193947.

13. Friedman JH, Hastie T, Tibshirani R. Regularization paths for generalized linear models via coordinate descent. Journal of statistical software. 2010;33:1-22.

14. Wright MN, Ziegler A. ranger: A fast implementation of random forests for high dimensional data in C++ and R. Journal of statistical software. 2017;77:1-17.

15. Thrun MC, Gehlert T, Ultsch A. Analyzing the Fine Structure of Distributions. PLoS ONE. 2020;15(10):e0238835. doi: 10.1371/journal.pone.0238835

16. Stier Q, Hoffmann J, Thrun MC. Classifying with the Fine Structure of Distributions: Leveraging Distributional Information for Robust and Plausible Naïve Bayes. Machine Learning and Knowledge Extraction. 2026;8(1):13. doi: 10.3390/make8010013.
