# Supplementary material for: Self-explaining artificial intelligence for the classification of B cell non-Hodgkin lymphoma: A diagnostic decision support study
Source: PLoS Med. 2026 Jul 13;23(7):e1004889. doi: 10.1371/journal.pmed.1004889 (PMC13421771; doi:10.1371/journal.pmed.1004889)
Supplement: S2 Checklist — BMJ. 2024;385:e078378. https://doi.org/10.1136/bmj-2023-078378. (PDF) [file pmed.1004889.s003.pdf]

TRIPOD+AI statement for FlowXAI

1. Title: The title identifies the study as the development and evaluation of a self-explaining artificial-intelligence system for diagnostic classification of suspected B-cell non-Hodgkin lymphoma (B-cell NHL) using multiparameter flow cytometry. The target population should be understood as samples from patients undergoing diagnostic work-up for suspected B-cell lymphoproliferative disease/B-cell NHL, rather than patients with already confirmed B-cell NHL. The model target is the correct diagnostic classification according to the reference diagnostic label.
2. Abstract: The abstract summarizes the clinical background, the development and evaluation datasets, the diagnostic-classification task, the main performance results, and the intended use as decision support. The relevant outcome for this study is correct diagnostic classification, not a clinical time-to-event endpoint such as progression-free or overall survival. We therefore report diagnostic performance measures rather than clinical outcome parameters.
3. Introduction: The Introduction describes the clinical context of diagnostic immunophenotyping for suspected B-cell lymphoma and the need for physician-support tools that can classify flow-cytometry samples in an interpretable manner. The study does not aim to predict clinical endpoints such as progression-free survival or overall survival. Instead, it addresses whether FlowXAI can support correct diagnostic categorization from flow-cytometry data. Because the source datasets were anonymized, patients or samples could not be assigned to sociodemographic subgroups such as age, sex, or ethnicity.
4. Objectives: The objectives are to develop and evaluate FlowXAI for diagnostic classification of suspected B-cell lymphoproliferative disease/B-cell NHL, to assess performance under repeated held-out evaluation and external benchmarking, and to provide interpretable case-level outputs for physician review. Thus, both development and evaluation of the diagnostic-classification system are within the scope of the study.
5. Methods/Data: The Methods begin with a detailed characterization of the datasets, including cohort origin, panel structure, sample numbers, and diagnostic reference labels. The datasets represent retrospective flow-cytometry samples from routine diagnostic workflows and are used to evaluate diagnostic classification rather than clinical prognosis.
6. Methods/Participants: The numbers and locations of participating centers are described and referenced. Further background information on the original cohorts is provided in the cited source publications. The effective analysis units are anonymized flow-cytometry sample files from patients undergoing diagnostic work-up, together with normal controls where applicable. Detailed treatment information and longitudinal clinical outcome data were not available.
7. Methods/Data preparation: The manuscript describes the main components of FlowXAI and the preprocessing steps used for flow-cytometry data. Additional information on compensation, transformation, and cross-site preprocessing was added in response to the reviewers. Sociodemographic variables were not available in the retrospective anonymized datasets and were therefore not used as predictors or subgroup variables.
8. Methods/Outcome: For the purposes of TRIPOD+AI, the outcome is the reference diagnostic category assigned to each sample and, in evaluation, whether FlowXAI correctly reproduces that reference diagnostic classification. The study does not investigate patient-level clinical outcomes such as progression-free survival, overall survival, treatment response, or prognosis. These clinical outcomes were not part of the research question and were not available in the source datasets.

9. Methods/Predictors: The model inputs are multiparameter flow-cytometry measurements derived from fluorescently labelled antibodies and light-scatter features. These are used as diagnostic predictors for classifying the reference diagnostic category. Clinical prognostic predictors were not collected or analyzed. The manuscript also discusses the challenge that lymphoma categories and diagnostic criteria evolve over time and that some WHO-defined entities may be only partially represented by a given immunophenotyping panel.
10. Methods/Sample size: Training, validation, data splitting, and repeated held-out evaluation are described in the Methods under “Generalization and validation protocol”. The study used the available retrospective datasets rather than a prospectively powered sample-size calculation. Sample numbers, class imbalance, and split sizes are reported to make the available evidence transparent.
11. Methods/Missing data: The MLL9F and PUM2 datasets were selected and processed as described in the Methods and Supplementary Methods. Missingness in clinical metadata could not be analyzed because demographic, treatment, and longitudinal clinical variables were not available in a complete and standardized form. Selection or exclusion of certain lymphoma entities is described in the Results and discussed.
12. Analytical Methods: The analytical workflow, including training/test separation, repeated held-out evaluation, external benchmarking, and performance metrics, is described in the Methods and Results. Because sociodemographic data were unavailable, subgroup fairness analyses could not be performed. The present manuscript focuses on model development and evaluation; deployment-level usability testing of a permanent clinical user interface was beyond the scope of this study.
13. Methods/Class imbalance: The manuscript describes class imbalance and the measures used to address it, including class-balanced train-test splitting and the use of Matthews correlation coefficient (MCC) as the primary global multiclass performance metric. Additional entity-specific and clinically oriented metrics are provided in the manuscript and Supplementary Information.
14. Methods/Fairness: Formal fairness assessment across sociodemographic groups could not be performed because age, sex, ethnicity, and other demographic variables were not available in the anonymized retrospective datasets. Benchmarking against alternative computational methods is described separately in the Methods, Results, Supplementary Methods.
15. Methods/Model output: FlowXAI outputs a diagnostic classification and a case-level trustworthiness score that stratifies cases into confident, probable, and challenging categories. The manuscript describes the performance measures used to evaluate these outputs, including MCC, accuracy, entity-specific contingency tables, one-vs-rest predictive values, ROC/PR analyses, and calibration of the trustworthiness output.
16. Methods/Training versus evaluation: The separation of training and test data is described in the “Generalization and validation protocol” section. Performance estimates are based on held-out test samples from repeated class-balanced train-test splits, while the PUM2 analysis is described as cross-site benchmarking under heterogeneous panel and tube conditions.
17. Methods/Ethical approval: Ethical approval and data-use information are supplied in the manuscript and Supplementary Information as applicable to the retrospective anonymized datasets.
18. Open science: Funding and competing-interest statements are provided. Protocol and registration were not applicable to this retrospective model-development and evaluation study. The data sources are previously published and referenced. Code availability has been updated in the revised manuscript, including access to the PLAIT platform/source code and analysis material where available for review and reproducibility.

19. Patient and public involvement: Patients and the public were not involved in study design, conduct, reporting, or interpretation. The study was based on retrospective anonymized flow-cytometry datasets.

20. Results/Participants: The Results report the numbers of sample files and diagnostic categories included in each dataset. Because the analysis used anonymized retrospective sample files, participant-level demographic and clinical characteristics were not available for reporting.

21. Results/Model development: The development of FlowXAI, including its unsupervised structural curation, supervised expert-committee classification, and trustworthiness output, is described in the Methods and Results.

22. Results/Model specification: FlowXAI is specified as a diagnostic classification model. The primary model output is the predicted diagnostic category, supplemented by a case-level trustworthiness score. The study does not model survival, treatment response, or other longitudinal clinical outcomes.

23. Results/Model performance: Model performance is presented in the Results with figures and detailed supplementary tables. The manuscript additionally reports clinically oriented one-vs-rest metrics, selective ROC and precision-recall analyses, and calibration of the trustworthiness output.

24. Results/Model updating: The manuscript describes the adaptation and extension of the previously described ALPODS algorithm within FlowXAI. The evaluation reported here concerns the developed diagnostic-classification workflow rather than post-deployment model updating.

25. Discussion/Interpretation: The Discussion summarizes the main findings in terms of diagnostic classification performance, interpretability, trustworthiness stratification, benchmarking, panel-dependent separability, and intended use as physician decision support.

26. Discussion/Limitations: The Discussion addresses key limitations, including retrospective data, restricted clinical metadata, evolving lymphoma classifications, class-specific separability, antibody-panel dependence, and the absence of prospective deployment testing.

27. Discussion/Usability of the model in the context of current care: The manuscript positions FlowXAI as a decision-support and teaching framework for expert users rather than an autonomous diagnostic system. Its reduced training-data requirement, case-level trustworthiness output, and interactive explanatory design may support physician review of flow-cytometry data in future clinical or educational settings.
