# Supplementary material for: Self-explaining artificial intelligence for the classification of B cell non-Hodgkin lymphoma: A diagnostic decision support study
Source: PLoS Med. 2026 Jul 13;23(7):e1004889. doi: 10.1371/journal.pmed.1004889 (PMC13421771; doi:10.1371/journal.pmed.1004889)
Supplement: Attachment — Submitted filename: response2reviewers_V9.docx [file pmed.1004889.s007.docx]

Dear Editor in Chief, dear Associate Editor Katarzyna Starowicz

thank you for handling our manuscript “Self-explaining Artificial Intelligence for the Classification of B cell Non-Hodgkin Lymphoma” and giving us the chance to accommodate the reviewer’s comments.

We also thank the editor and reviewers for their careful assessment of our manuscript and for the constructive comments. We have revised the manuscript substantially in response to these suggestions, including additional methodological clarification, e.g., more information of validation, expanded benchmarking, trustworthiness calibration, clinically oriented performance analyses, TRIPOD statement, and additional supplementary material. Please find below our detailed point-by-point responses to the reviewers comments marked in red.

Reviewer #1

“This manuscript presents FlowXAI, an AI system for BNHL classification from flow data. The topic is important and the dataset is sizable however several key methodological details are unclear and the presentation is difficult to follow.”

R1.1

**Reviewer point:** *"The purpose and advantages of TM over existing QC methods are unclear. Because tile boundaries are case-specific, it is uncertain how biological variation is distinguished from technical noise." "*

We thank the reviewer for the thorough review, and agree that the original manuscript did not position Tile Mining (TM) clearly enough relative to routine laboratory quality-control procedures. TM is not intended to replace instrument QC, compensation verification, staining controls, or laboratory SOPs. Rather TM is an unsupervised sample-level structural screening step that flags atypical cases for expert review and prevents such cases from disproportionately influencing representative-case selection and supervised training. Because TM cannot by itself determine whether atypicality is technical or biological in origin, we propose it as a human-in-the-loop failsafe: TM-flagged cases are highlighted for review, excluded from training, but retained for downstream testing and manual inspection. In the present study, TM also supported representative-case selection and substantial reduction of training requirements without filtering the evaluation sets. This positioning is consistent with the current manuscript and SI and manual review of 100 atypical samples. Tile boundaries were derived from preprocessed data, which is stated now in the revised manuscript:

“After compensation and signed-log transformation, each sample was represented by the concatenated nine-tile summaries of all unique marker pairs as follows… Hence, σ_(i,t) summarizes the average robustly standardized deviation of the case from the reference tile profile. Between-sample comparison is therefore performed in a common normalized tile-feature space rather than on identical absolute raw-value borders. “

In supplementary figure 2 we reference ”… 100 evaluated samples are available as fcs files at ~~10.5281/zenodo.7744104~~ “, link updated to h[ttps://doi.org/10.5281/zenodo.19681022](https://doi.org/10.5281/zenodo.19681022)” in which we investigated the issue of biological variation vs technical noise manually: “Human experts assume that red blood cells are predominant in atypical samples for the reason that most recorded events do not stain positive for CD45 or other leukocyte antigens. Other presumed reasons for the strong alterations were incomplete erythrocyte lysis, irregular antibody staining patterns or other aberrations, most likely acquisition artifacts.“

The discussion in the manuscript was revised to

“Tile Mining (TM) is not a substitute for conventional laboratory quality-control procedures such as instrument monitoring, compensation validation, or staining controls. Instead, TM operates at the level of the sample files and evaluates whether the multivariate event structure of a case is typical relative to the reference cohort after standard preprocessing. TM identifies structural atypicality relative to the reference cohort but does not determine whether the underlying cause is technical, biological, or both. In the present study, TM was therefore used as a human-in-the-loop structural curation step for machine-learning training: atypical cases were excluded only from representative-case selection and supervised training, whereas independent evaluation retained both typical and atypical cases.”

R1.2

**Reviewer point:** *"The strangeness metric is not mathematically convincing and between-sample comparability is not demonstrated."*

We appreciate this point and agree that the previous description of strangeness and between-sample comparability was too informal. After compensation and signed-log transformation, each sample is mapped to a fixed-length tile profile by summarizing all unique marker pairs on a fixed 3×3 grid per pair. The resulting case profile is centered against a robust reference profile and robustly standardized feature-wise; the sample-level strangeness score is then computed as the average standardized deviation across all tile features. Accordingly, comparability is achieved in the common normalized tile-feature space, not by requiring identical absolute raw-value tile borders across cases.

The manuscript is revised to

“After compensation and signed-log transformation, each sample was represented by the concatenated nine-tile summaries of all unique marker pairs as follows. …

For a marker set of size d, let $H_{i}\left( d \right)$ denote the concatenated tile profile of case $i$. Across all marker pairs, this procedure yields

\#$H_{i}\left( d \right)=9*\frac{d(d-1)}{2}$.

tiles per sample. For d=11 markers, this corresponds to$\#H\left( d=11 \right)=495$ tiles. For each tile $h\in H(d)$, the density $p(h)$ is defined as the proportion of events falling into that tile:

$$p\left( h \right)=\sum_{e \in E} f_{e},f_{e}=\left\{ \begin{matrix} 1 & e \epsilon h \\ 0 & e\notin h \end{matrix} \right.$$

where E denotes the set of all measured events in the sample. The resulting count matrix is internally normalized ensuring comparability across tiles within each sample.

Let $\overset{ˉ}{T}$ denote the robust reference tile profile estimated from the cohort. TM centers each $H_{i}(d)$ in reation to T by applying robust feature-wise standardization, yielding values $p_{ij}^{*}$. For a given patient i and tube t, the normalized tile values are aggregated to yield a strangeness value:
$\sigma_{i,t}=\frac{100}{p\#H_{i}\left( d \right)}\sum_{j=1}^{\#H_{i}\left( d \right)} p_{ij}^{*}(h)$.

Hence, $\sigma_{i,t}$ summarizes the average robustly standardized deviation of the case from the reference tile profile. Between-sample comparison is therefore performed in a common normalized tile-feature space rather than on identical absolute raw-value borders.

R1.3

**Reviewer point:** *"The manuscript claims successful generalization to an external dataset but provides no information on normalization steps, compensation harmonization, scaling across instruments, or cross-site preprocessing. Without these details, robustness claims cannot be evaluated. "*

The reviewer mentions steps which are of tremendous importance for a clinical application setting. However, these are important issues which have to the best of our knowledge not been resolved optimally yet by neither manufacturers nor flow cytometry companies and are out of scope of the current work.

We agree that normalization, compensation, and cross-site preprocessing are important for clinical deployment. In the present retrospective study, we used the available laboratory compensation information and signed-log transformation before TM and ALPODS feature construction. Because MLL9F and PUM2 differed in laboratory, antibody panel, and tube configuration, we revised the manuscript to frame PUM2 as a cross-site benchmark under heterogeneous conditions rather than as a fixed-model external validation on identical predictors. The manuscript in the methods section was revised to

“All FCS files were processed using the available laboratory compensation information and transformed by signed-log scaling. Because MLL9F and PUM2 were acquired in different laboratories with different antibody panels and tube configurations, the PUM2 analysis was employed as a cross-site benchmark of the FlowXAI framework under heterogeneous conditions. MLL9F was measured using several instruments of the same type.”

R1.4

**Reviewer point:** *"Technical robustness is not assessed as no experiments to examine the impact of instrument variability, compensation perturbations, fluorescence drift, or antibody lot differences-factors known to affect MFC-based AI systems were performed."*

Yes, dedicated perturbation experiments addressing compensation perturbation, fluorescence drift, lot-to-lot variability, or instrument-specific effects were not performed in the present study. The PUM2 benchmark captures some real-world cross-site and cross-panel variation. Of note, The dataset accessed from Zhao et al, was measured on several flow cytometers so inter-instrument variability from the same flow cytometer type does not seem to have an influence.

The present data support repeated held-out evaluation within MLL9F and cross-site benchmarking on PUM2 under routine preprocessing, but they do not isolate individual technical sources of variation. We therefore narrowed the robustness claim in the revised manuscript as follows:

“We did not perform dedicated perturbation experiments for compensation, fluorescence drift, lot-to-lot variability, or instrument-specific effects although MLL9F dataset was measured on several instruments. Accordingly, we restrict our claims to the robustness directly evaluated here: repeated held-out testing within MLL9F with limited instrument variability, and cross-site benchmarking on PUM2 under routine laboratory preprocessing. Dedicated technical robustness analyses remain an important topic for future work.”

R1.5

**Reviewer point:** *"Removing atypical cases risks eliminating biologically meaningful or rare variants, artificially inflating performance. The consequences of TM filtering on specific lymphoma subtypes are not quantified.""*

We agree that the effect of TM filtering on biologically meaningful or rare variants must be transparent. We had addressed this issue directly by surfacing the entity-wise typical/atypical counts that are already available in the supplementary information Tabe 3 and SI Figure 6. This allows readers to assess whether rare or immunophenotypically heterogeneous categories are disproportionately flagged as atypical. Importantly, TM-flagged atypical cases were removed only from representative-case selection and model training, not from the independent test sets. The detailed entity-specific contingency tables for Tables 4-9 already list typical and atypical counts per entity for both datasets. Hence, quantification of the consequence of TM filtering was shown Fig. 3f, explained on page 15: „When TM-based elimination of atypical samples was applied to the training sets, a modest improvement in accuracy and MCC was observed across all degrees of trustworthiness (Fig. 3f). This effect was most pronounced in the challenging category, where false negative rates decreased.” Thus, TM is interpreted as a training-stabilization and human-in-the-loop flagging step, rather than as a mechanism for excluding clinically difficult cases from evaluation.

In the discussion of the manuscript the following sentence was added:

‘To quantify the effect of TM on class composition, we report the numbers of typical and atypical cases for each lymphoma entity in MLL9F and PUM2 and cross-reference the entity-specific performance tables in the Supplementary Information. TM filtering was applied to representative-case selection and training only; all independent test sets retained both typical and atypical cases. We therefore interpret TM as a training-stabilization step and human-in-the-loop flagging mechanism, rather than as a method for excluding clinically difficult cases from evaluation.”

R1.6

**Reviewer point:** *"* Although the system is described as selfexplaining, few concrete examples of clinically interpretable rules are shown, and no comparison with expert gating is provided*."*

To relate the FlowXAI explanations to conventional expert gating, four HCL samples (rows A–D) were manually reviewed by an experienced flow-cytometry expert using the FlowXAI-extracted FCS files in Kaluza™ software version 2.1 on a logarithmic scale. We refer to SI Figure S3, S4 and S5 contain manual Kaluza-style review for HCL examples and the corresponding explanation given in the SI text and revise the Figure legend of Figure S3 to

“Rows A–D show four representative HCL samples in which FlowXAI’s explanatory output is compared with conventional expert gating. The FlowXAI-extracted FCS files were manually reviewed by an experienced flow-cytometry expert in Kaluza™ software version 2.1 using logarithmic scaling.”

R1.7

**Reviewer point:** *"Grouping MBL/CLL/PL and handling of HCL within the hierarchy appear model-driven rather than clinically motivated. Given the changes in the classification; its not possible to reconcile these old labels to the new WHO classification "*

We retained the historical dataset label structure to preserve comparability with prior automated immunophenotyping studies (see first chapter), and we now clarify explicitly that this label structure differs from current WHO terminology. The differentiation between MBL, CLL and PL requires morphological and clinical knowledge, i.e. the amount (percentage) of prolymphocytes among the total population of lymphocytes, the total amount of peripheral B-lymphocytes (for MBL < 5000/ul) or the absence of lymphadenopathy or splenomegaly. If the aberrant B-cell population is tiny an immunophenotypic report will refer to the differential diagnoses CLL/MBL based on clinical rather immunophenotype-based distinction. This particular problem had prompted us to perform the unbiased cluster approach described in Fig. 1 which illustrates an AI based lymphoma grouping. Of note, for comparison with previous approaches of automated immunophenotyping we had to be in line with the categorization of the published approach. Nevertheless, combining the clinical approach with the unsupervised classification allows in your view the adaption of the hierarchical decision tree provided in Figure 2 to the new WHO classification given new data.

The manuscript was revised to

“The grouping of MBL, CLL, and PL in the present study reflects routine immunophenotypic reporting constraints, because definitive distinction between these entities requires additional morphological and clinical information beyond flow cytometry alone. We retained the historical dataset label structure for comparability with prior automated approaches which differs to current WHO terminology.”

R1.8

**Reviewer point:** *"* Weblink does not work. Maybe use a weblink shortener? Code is promised but not available for review, and key algorithmic steps (TM, ALPODS committee) lack sufficient technical detail*."*

We verified the PLAIT portal link https://plait.mathematik.uni-marburg.de/ before resubmission and added a stable code-availability statement. The PLAIT source code is available through Zenodo (<https://zenodo.org/records/19661905>.), and the manuscript now specifies where the code can be inspected during review. In addition, we provide the complete exemplary decision logic for the PUM2 TM, ALPODS committee as Supplementary material.

R1.9

**Reviewer point:** *"* Perentity performance, calibration, ROC/AUC-PR curves, and confusion matrices should be included to assess realworld utility given class imbalance.*."*

Because the study addresses a hierarchical multiclass diagnostic workflow, we retained MCC as the primary global summary metric and used the contingency tables to make the class-wise behavior transparent. Confusion matrices had been given for every scenario in SI, Table 4-10, because we felt that this information would enlarge the main text and distract from the emphasis of our work. In the revised manuscrut we added trustworthiness calibration, including reliability plots, ECE, and Brier score, and selective one-vs-rest ROC and precision-recall analyses based on FlowXAI trustworthiness categories. We also improved cross-referencing to the entity-specific contingency tables in the Supplementary Information by stating in the last sentence of the manuscript result section:

“Detailed entity-specific contingency tables are provided in the Supplementary Information Table 4-10.”

We propose a new subparagraph in the revised manuscript to the results section:

“Trustworthiness validation.“

FlowXAI’s trustworthiness output was informative with respect to diagnostic reliability [1, 2]. The observed correctness rate increased from 79.5% (95% CI 79.3% to 79.8%) for challenging cases to 86.9% (95% CI 86.6% to 87.1%) for probable cases and 94.3% (95% CI 94.2% to 94.4%) for confident cases. Overall, calibration of predicted correctness showed low average miscalibration (ECE 0.024; Brier score 0.089). In the upper reliability bins, calibrated predicted correctness ranged from 90.1% to 90.6%, whereas observed correctness ranged from 93.5% to 94.0%, indicating conservative calibration in the highest-confidence region.

Selective one-vs-rest ROC and precision-recall analyses based on the ordered FlowXAI trust strata showed strongest selective discrimination for NC, CLL-like, and HCL, intermediate performance for MZL, and limited selective utility for MCL, FL, and LPL (Supplementary Selective one-vs-rest ROC and precision-recall analysis from FlowXAI categories of trustworthiness Figs. 7–8).

We revised the results section to

“Selective one-vs-rest ROC and precision-recall analyses based on the ordered FlowXAI trust strata showed strongest selective discrimination for NC, CLL-like, and HCL, intermediate performance for MZL, and limited selective utility for MCL, FL, and LPL (Supplementary Selective one-vs-rest ROC and precision-recall analysis from FlowXAI categories of trustworthiness Figs. 7–8).”

And to the discussion:

“The trustworthiness validation supports the clinical usefulness of FlowXAI’s internal trustworthiness stratification. Lower-trust cases carried a substantially higher error risk, whereas confident cases were associated with markedly higher observed diagnostic reliability. Selective one-vs-rest ROC and precision-recall analyses indicate that FlowXAI trustworthiness categories have entity-specific clinical value: high-trust predictions are most actionable for NC, CLL-like cases, and HCL; MZL shows intermediate decision-support utility; and MCL, FL, and LPL remain predominantly expert-review entities.”

R1.10

**Reviewer point:** *"The manuscript's organization is dense and repetitive.* TM, the diagnostic hierarchy, and background concepts are described multiple times.*"*

We revised the text to reduce repetition, including tile mining rationale, and trustworthiness concept. We shortened and clarified the **FlowXAI overview** so that the methods now start with the clinical purpose and three major functions instead of a dense technical description. The FlowXAI technical description is moved to the supplementary. In addition, we propose to include the denser methodological details as given in response to Query 1.9 to the Supplementary Methods where appropriate.

R1.11

**Reviewer point:** *"Some figures require clearer legends and clinical context."*

The figures and legends in the revised manuscript now provide clearer clinical guidance. Especially Figure 1 was revised to

The topographic map visualizes high-dimensional distances and densities between Databionic-swarm-projected points and allows assessment of intrinsic data structure represented by valleys and mountain ranges. Each point represents a patient sample composed of three tube-specific files from the MLL9F dataset. Coloring indicates clinical labels for lymphoma classes and was not used during self-organization. Within the topographic map, valleys, and basins are depicted as clusters, while the watersheds of hills and mountains serve as cluster boundaries by the following color scheme. The blue colors represent lower elevations (e.g., sea level), green and brown for intermediate elevations (e.g., low hills), and various shades of white for higher elevations (e.g., snow-covered mountains). Hypsometric tints use distinct surface colors to represent elevation ranges, with contour lines integrated into a specific color scheme. The elevation ranges are mapped to high-dimensional data distances and densities of the projected points. The exact mapping is defined in [65]. Additionally, the visual borders in the topographic map exhibit cyclic connections with periodicity.

**A** The by a topographic map visualized self-organization of a subset of the MLL9F dataset (3,000 NC, 3,000 CLL-like, 3,000 non-CLL-like B-NHL samples) reveals two dominant valleys that contain the clusters in SI table 1, two outlier-associated structures represented as outlier groups in the same table, and isolated atypical samples depicted as various outliers in above referenced table.

**B** The same topographic map highlights a pronounced structural separation between normal controls (NC) and B-NHL samples (see SI Tab 1 for contingency table.). Without TM incorporation, many single outliers scatter across the topographic map, illustrated as single dots in single valleys.

**C** After application of Tile Mining (TM) for elimination of structurally atypical samples, the topographic map representing the self-organization of lymphoma cases reveals three dominant valleys that contain the clusters in SI table 2 and markedly fewer isolated outliers.

**D** Mapping of clinical labels onto the TM-filtered topographic map shows correspondence of major structures with CLL-like entities, HCL, and all other lymphomas (AOL), while some entities remain not clearly separable (see SI Table 2 for contingency table).

In addition, we revised the main-text and supplementary figure legends to define abbreviations in place and clarified the clinical interpretation.

R1.12

**Reviewer point: “**The approach is interesting, however the manuscript requires substantial clarification and additional experiments—particularly around TM, domain shift, and robustness—before the publication.”

We thank the reviewer for this overall assessment. The revised manuscript addresses these concerns by expanding the TM description and thresholding rationale, clarifying domain-shift and preprocessing assumptions, narrowing claims about technical robustness, adding conventional single-tube baselines, adding trustworthiness calibration and clinically oriented performance analyses, improving case-level explanation examples, and revising figure legends and supplementary material.

Reviewer #2

“This is an interesting study on using Self-explaining Artificial Intelligence to classify B cell Non-Hodgkin Lymphoma. However, there are a few major issues needing attention.”

R2.1

**Reviewer point:** *"* 1. One standing out issue is that the paper is mostly about explaining the advantages of its own FlowXAI system, but lacks systematic and like for like comparisons with other existing machine learning and statistical methods, apart from some ad hoc comparisons in parts of the paper. The proposed FlowXAI system looks well structured, logical and useful AI diagnostic tool, and could be an alternative to the existing ML algorithms if there were evidence from direct like for like comparison with existing mainstream algorithms. But so far, it is mostly talking by by itself and lacks systematic and convincing evidence showing novelty and better performance.”

We thank the reviewer for these important comments agree that additional direct benchmarking strengthens the manuscript. In the revised version, we therefore added conventional single-tube reference analysis on the MLL9F dataset with random forest (ML method) and multinomial elastic-net (glmnet, statistical method**)** classifiers, evaluated on the same 100 repeated class-balanced 80/20 train-test splits as FlowXAI at hierarchy level 3:

The results section was revised to:

“To provide a conventional single-tube reference, we benchmarked random forest and multinomial elastic-net classifiers trained on flowFP fingerprints against FlowXAI at hierarchy level 3 across the same 100 repeated train-test splits (Fig. 5). Mean ± SD test MCC values for random forest were 0.79 ± 0.01, 0.72 ± 0.01, and 0.71 ± 0.01 for tubes 1–3, and for glmnet 0.79 ± 0.01, 0.73 ± 0.01, and 0.72 ± 0.01, respectively. The FlowXAI confident stratum reached 0.88 ± 0.01, 0.86 ± 0.01, and 0.81 ± 0.02 across tubes 1–3.”

We added to the discussion:

“Conventional fingerprint-based baselines provide a useful sample-level reference, while FlowXAI’s contribution extends beyond raw classification to hierarchical multi-tube decision support and native trustworthiness assessment.”

And provide Figure 5 in the main manuscript:

“Fig. 5. Benchmarking FlowXAI against conventional single-tube baselines on MLL9F at hierarchy level 3.

Benchmarking was performed using the same 100 repeated class-balanced 80/20 train-test splits as in Fig. 3 (15,594 training samples and 3,899 test samples per repetition). For each tube, sample-level flowFP fingerprints were generated and used as input to a random forest (rf) and a glmnet classifier. MD-plots show the distributions of held-out Matthews correlation coefficient (MCC) values across the 100 test folds for the two baseline classifiers and for FlowXAI stratified by trustworthiness: confident (gold), probable (green), and challenging (blue). Where shown, magenta outlines denote Gaussian fits to the empirical MCC distributions. Mean ± SD MCC values were: ube 1: RF 79% ± 1%, glmnet 79% ± 1%, FlowXAI confident 88% ± 1%, probable 78% ± 3%, challenging 68% ± 5%

tube 2: RF 72% ± 1%, glmnet 73% ± 1%, FlowXAI confident 86% ± 1%, probable 74% ± 3%, challenging 68% ± 3%

tube 3: RF 71% ± 1%, glmnet 72% ± 1%, FlowXAI confident 81% ± 2%, probable 69% ± 3%, challenging 56% ± 6%

Abbreviations: MCC, Matthews correlation coefficient; MD, mirrored density, rf, random forest, multinomial elastic-net, glmnet, rf, random forest”

In addition, we kindly point out that comparisons already were performed with a published deep-learning B-cell immunophenotyping approach and with CITRUS (page 14 to 15, Fig. 3) and with CITRUS (see Fig. 4). For comparison CITRUS was chosen „because of its conceptual similarity to population-driven immunophenotyping and its previously demonstrated high performance relative to other methods [1, 24].“

The comprehensive literature research which leads to our decision to restrict the benchmarking significantly is explained in detail in SI Methods page 17-19. The usage of typical machine learning algorithms is already limited for very small sample sizes as discussed in previous work of Ultsch et al. 2024.

We also clarify that the trustworthiness categories are native outputs of FlowXAI and should therefore be interpreted as validation of the framework’s internal self-assessment rather than as a direct algorithm-to-algorithm benchmark. We extended the revised manuscript in the results section with

“Because trustworthiness categories are native outputs of FlowXAI, the present analysis should be interpreted as validation of the framework’s internal trustworthiness stratification rather than as a direct algorithm-to-algorithm comparison.”

We revised the introduction of the manuscript to position the method accordingly:

“The novelty of FlowXAI lies in the combination of interpretable committee-based decision support, native trustworthiness stratification, and reduced training requirements, rather than in a claim of universal superiority over all alternative classifiers.”

R2.2

**Reviewer point:** *"* Also, *paper is written with substantial technical details with AI jargon and is quite difficult to follow by the clinical audience of PLOS Medicine. The paper may be more suitable for digital health journals elsewhere."."*

Indeed, medical and mathematical phrases are different jargons. At the same time, transparent reporting of the methodological workflow is important for reproducibility, and several other reviewer comments requested additional methodological clarification. We revised the text to reduce repetition, including tile mining rationale, and trustworthiness concept. We shortened and clarified the FlowXAI overview so that the methods now start with the clinical purpose and three major functions instead of a dense technical description. The FlowXAI technical description is moved to the supplementary.

In the revised manuscript, we respectfully believe that the so far targeted revisions improve readability for the readership of PLOS Medicine without reducing the methodological transparency of the study. Metrics that are more commonly used in a clinical audience were added in response to R1.9.

R2.3

**Reviewer point:** *"* 2. *Performance, Page 14. It says 'Performance within the confident category showed consistently high MCC values, ranging from 88% to 91% (median 90%), with an approximately Gaussian distribution (magenta frame) (Fig. 3d). The probable category yielded intermediate performance (median MCC 77%), while the challenging category exhibited lower and more variable performance (median MCC 60%). These results indicate that FlowXAI's internal self-assessment is informative with respect to diagnostic reliability'. What other mainstream algorithms can offer for these 3 categories based on the same dataset? The proposed system doesn't seem anything special here."*

We agree that a direct comparison of the confident/probable/challenging categories with another method would be informative where available.

We therefore revised the manuscript to clarify that this analysis validates the informativeness of FlowXAI’s trustworthiness stratification rather than serving as a direct algorithm-to-algorithm benchmark.

However, these categories are native outputs of FlowXAI’s internal self-assessment and are not reported in directly analogous form by the published comparator systems available for this task. We therefore revised the manuscript to clarify that this analysis validates the informativeness of FlowXAI’s trustworthiness stratification rather than serving as a direct algorithm-to-algorithm benchmark. The decision for our design was prompted by the human-in-the-loop approach, because immunophenotyping physicians include data quality in their reports. To the best of our knowledge, we do not know any published approach for AI based B-cell immunophenotyping which includes this judgement step on a single sample level. For external context, the manuscript already reports comparison with a published deep-learning B-cell immunophenotyping approach and with CITRUS, and we now explain more explicitly why other identified approaches were not directly comparable to the same task formulation.

The revised manuscripts states:

“Within FlowXAI, the native trustworthiness categories were informative with respect to diagnostic reliability. Because the published comparator systems considered here do not provide directly analogous confident/probable/challenging outputs for the same task (see SI Methods: Literature evaluation for seeking benchmark algorithms), this analysis is presented as a within-framework validation rather than as a cross-algorithm benchmark.”

R2.4

**Reviewer point:** *"* 3.Figure 1 shows the unsupervised clustering results. There are many well-establishing clustering algorithms around and they can do at least similar job. *It is not clear what the authors showed here which has never been done by others."*

We have therefore revised the manuscript to present Figure 1 explicitly as an exploratory, label-independent analysis of structural embodiment that provides biological context for the downstream classification task. The purpose of this passage related to Fig. 1 was to sensitize the reader for the general challenge of AI training if categories/classes vary.

We also separated the Methods and Results more clearly: the Methods now explain why a self-organizing swarm-intelligence framework was chosen for this exploratory analysis, whereas the Results focus only on the structures observed in the data. In particular, we clarify that the number and geometry of meaningful structures were not known a priori, that the analysis was intended to assess whether the measured immunophenotypic space contains clusterable structure corresponding to clinical categories, and that the Databionic Swarm was chosen because it provides a topographic visualization of intrinsic sample structure without requiring a prespecified number of clusters.

The methods section paragraph was revised to

“Exploratory analysis of structural embodiment in lymphoma immunophenotypes

To explore how clinically defined lymphoma entities are reflected in high-dimensional multiparameter flow-cytometry (MFC) data, we performed an exploratory structural-embodiment analysis independently of the clinical labels. Clinical labels remain the authoritative diagnostic standard for patient care, but they are assigned within evolving WHO classification systems and may show inter-observer and inter-institutional variability. In addition, some clinically relevant distinctions depend on information not contained in the present immunophenotyping panel, such as morphology, cytogenetics, molecular data, or clinical context. We therefore used this analysis to assess how strongly the measured MFC data embody clinically used categories, rather than to validate or challenge the clinical diagnoses themselves. In this context, structural embodiment refers to two complementary aspects: first, whether clinically established knowledge is represented in the measured data, and second, whether the resulting data-driven structures correspond to expert clinical labels.

For this purpose, we used the Databionic Swarm, a swarm-intelligence and self-organization framework for high-dimensional structure analysis [3]. The method combines projection-based self-organization with generalized U-matrix visualization[4, 5], yielding a topographic map in which valleys indicate regions of high similarity and hills indicate structural dissimilarity between samples. Because this step is fully unsupervised, any separation between presumed categories emerges from the self-organization of the data rather than from label information.

We chose this framework because the number and geometry of biologically meaningful structures in the immunophenotypic space were not known a priori. In contrast to many commonly used clustering algorithms, which optimize a predefined objective function and often require assumptions about cluster number, compactness, or geometry, the present exploratory approach does not require specification of the number of groups in advance and is well suited to assessing heterogeneous, irregularly shaped structures in high-dimensional biomedical data [6, 7]. In conjunction with clusterability diagnostics, such self-organizing approaches are also useful for evaluating whether the data support partitioning into distinct groups at all, rather than forcing a cluster solution where the underlying structure is weak or continuous[8, 9].

For the present study, full-cohort computation was performed using a multicore CPU TRIPOD of the Databionic Swarm [10] ”

The results result section was revised to

“To obtain a label-independent bioinformatic view of lymphoma immunophenotypes, we applied the structural-embodiment analysis to the previously published MLL9 dataset, comprising normal controls (NC) and the entities CLL, PL, MBL, HCL, MCL, MZL, LPL, and FL.

The resulting topographic map (Fig. 1a) revealed two dominant structures, two outlier-associated structures, and a number of highly atypical individual samples. At the global level, the analysis showed a clear separation between presumed healthy individuals and B-cell non-Hodgkin lymphomas (B-NHL) (Fig. 1b). Only 3 of 2,998 NC samples were structurally located within the B-NHL-dominated region, whereas a substantial subset of B-NHL samples was embedded within the NC-associated structure (SI Table 1). This asymmetric pattern indicates strong global separation between normal and neoplastic samples, while at the same time highlighting marked structural heterogeneity within the lymphoma group.

Within the lymphoma compartment, some entities were structurally more distinct than others. In particular, CLL-like cases formed a comparatively well-separated structure, whereas entities with more subtle or overlapping immunophenotypic features, such as MZL, LPL, and FL, were less clearly isolated at this global level of analysis.

“

R2.5

**Reviewer point:** *"* 4.External validation, Page 9. It says "To assess robustness under domain shift, FlowXAI was further evaluated using the PUM2 dataset, an independent external benchmark dataset originating from a separate diagnostic center and employing a different antibody panel and tube configuration". But, not sure it's make sense to test on datasets "employing a different antibody panel and tube configuration*. Also, the system seemed being trained again in the external dataset?"*

We agree that the heterogeneous panel structure of PUM2 could be described more explicitly. Because MLL9F and PUM2 differ in laboratory, antibody panel, and tube configuration, the PUM2 experiment should not be interpreted as a simple fixed-model transfer on identical predictors. We therefore revised the manuscript to frame PUM2 as a cross-site benchmark of the FlowXAI framework under heterogeneous conditions.

The manuscript was revised to

“Because MLL9F and PUM2 were acquired in different laboratories with different antibody panels and tube configurations, the PUM2 analysis was designed as a cross-site benchmark of the FlowXAI framework under heterogeneous conditions.”

Yes, and we now state explicitly that PUM2 was used as a cross-site benchmark under heterogeneous panel and tube conditions, not as a fixed-model transfer experiment on identical predictors. This distinction is now reflected consistently The question overlaps partly with Reviewer 1 1.3. The manuscript was revised to

R2.6

**Reviewer point:** *"Ultimately, results from external validation should be regarded as the final for model/system performance. However, in this paper, most performance results were from the training dataset which is inadequate."*

Indeed, external validation deserves particular emphasis to investigate concept drift. We respectfully clarify, however, that the principal performance estimates reported for MLL9F were not obtained on the training dataset, but on held-out test partitions from 100 repeated class-balanced train-test splits. The hold-out test partitions were not used for parameter optimization and any kind if information leakage between training and test set was prohibited. These results therefore represent internal validation on unseen samples from the same source population (“structural equality”). Generalizability estimation was performed in accordance to AI and statistical literature. Among plethora of literature, we refer to

Varma, S., Simon, R. Bias in error estimation when using cross-validation for model selection. BMC Bioinformatics 7, 91 (2006). https://doi.org/10.1186/1471-2105-7-91

Kohavi, R. Artif. Intell. 14, 1137–1145 (1995).

Walsh, I., Fishman, D., Garcia-Gasulla, D. et al. DOME: recommendations for supervised machine learning validation in biology. Nat Methods 18, 1122–1127 (2021). https://doi.org/10.1038/s41592-021-01205-4

Stier, Q.,Hoffmann, J. & Thrun, M. C.: Classifying with the Fine Structure of Distributions: Leveraging Distributional Information for Robust and Plausible Naïve Bayes, Machine Learning and Knowledge Extraction (MAKE), Vol. 8(1), 13, DOI: [10.3390/make8010013](https://doi.org/10.3390/make8010013), MDPI, 2026.

The manuscript stated clearly that the results reported are not on the training dataset but on the test data set upon repeated cross-validation, e.g., in the introduction:

“FlowXAI was evaluated using repeated cross-validation on 19,493 peripheral blood samples and further assessed on an independent external benchmark dataset generated at a separate diagnostic center using a different antibody panel.”

The section Generalization and Validation protocol stateted:

To evaluate generalizability and robustness, FlowXAI was assessed using repeated cross-validation and an independent external benchmark dataset. For the MLL9F dataset, 100 repetitions of class-balanced 80/20 train–test splits were performed (N = 15,594 training samples and N = 3,899 test samples), with and without Tile Mining (TM)–based elimination of atypical samples from the training set by unsupervised tile mining. This design ensured that unsupervised sample-quality assessment did not influence the composition of the test sets and allowed quantification of potential performance degradation when atypical samples were included.

In contrast to prior studies relying on single split evaluations [11], repeated cross-validation was employed to simultaneously estimate expected generalization performance and assess variability across random partitions, thereby reducing the risk of optimistic bias [12, 13].

We increase the generalization ability even a step further than, to our knowledge ever before:

“To quantify the effect of training data reduction, an additional experiment was conducted in which only 512 representative training samples (256 normal controls and 256 B-cell lymphoma samples) were used. For this experiment, 32 samples per lymphoma entity were selected once from the pool of typical samples identified by TM, and the resulting model was evaluated on all remaining samples, with performance reported separately for typical and atypical cases.

“

In sum, this distinction follows standard methodological guidance for machine-learning validation and prediction-model evaluation.

The additional PUM2 experiment addresses a different question, namely cross-site benchmarking (“external validation”) under heterogeneous experimental conditions (i.e., changing laboratory (other staining procedures, etc.), panel and flow cytometer). Other types of concept drifts (i.e., European laboratory accepts samples from Asia) deserve particular emphasis by another external validation but it is outside of the scope of our current work.

The manuscript was revised to

“A fixed-model evaluation on an independent matched-panel cohort would further strengthen transportability assessment and remains an important next step beyond the scope of the current work.”

R2.7

**Reviewer point:** *"* Although this is an AI application paper, it would be good to follow the TRIPOD statement for clinical prediction models*."*

We thank the reviewer for this important suggestion. We agree that transparent reporting according to TRIPOD+AI is appropriate for clinical AI model development and evaluation studies. We therefore added a completed TRIPOD+AI checklist as Supporting Information (TRIPOD statement for FlowXAI.docx) and inserted an explicit reporting statement in the Methods. Where individual checklist items were not applicable to the present retrospective diagnostic-classification study, this is stated in the checklist.

We revised the manuscript in the introdution to

„Moreover, transparency of machine learning algorithms is one of the major aspects of the harmonized TRIPODS+AI consensus guidelines which define the major prerequisites for reporting prediction and diagnosis models (21).“

And the methods section to

“For this study the S1 Checklist according to the TRIPOD+AI reporting guidance is provided (21).”

Reviewer #3

“This manuscript presents FlowXAI, an artificial intelligence framework combining an unsupervised preprocessing step (Tile Mining, TM) with a rule-based expert committee (ALPODS) to support the diagnosis of chronic B-cell lymphoproliferative disorders from multiparametric flow cytometry data. The objective of developing an interpretable and clinically aligned decision-support system is timely and addresses an important need in hematopathology.
The integration of unsupervised structure analysis, hierarchical decision-making, and explainability represents a clear strength of the work. The involvement of expert clinical teams, together with the use of real-world datasets, further enhances its relevance. In addition, the extensive supplementary material provides valuable insight into the behavior of the system.

That being said, several aspects of the methodology, validation strategy, and presentation would benefit from further clarification and refinement. Addressing the points below would substantially improve the transparency, reproducibility, and clinical interpretability of the study.”

R3.1

**Reviewer point:** *"The manuscript emphasizes the interpretability of the ALPODS framework, yet the underlying decision logic remains only partially accessible."*

We thank the reviewer for the helpful review. In the present study, interpretability is operationalized primarily at the level of case-specific explanatory outputs, namely the identification of diagnostically relevant cell populations, human-interpretable dot-plot outputs, and a traceable workflow that can be inspected by the user as stated in the manuscript:

“We have created a live, interactive demonstration of our FlowXAI platform at <https://plait.mathematik.uni-marburg.de/> with its source code available at https://zenodo.org/records/19661905. This online portal is specifically designed so that physicians, computational scientists, and readers can explore our proposed step-by-step classification workflow: Visitors can upload data (or select from example datasets) and see how FlowXAI automatically identifies relevant cell populations, performs classification, and provides self-explaining visual outputs. Each FlowXAI decision is grounded in traceable rules that mimic the logic of human experts. FlowXAI provides these understandable explanations stored in the FCS format.

“

To make this clearer, we revised the manuscript to strengthen the Methods description to:

“We provide inspectable case-level explanatory outputs, an interactive portal, reviewer-accessible source code, and exemplary decision logic that allow readers to trace diagnostically relevant populations and decision-support output.”

In addition, exemplary for PUM2, we now provide the full decision logic within an SI download attached to the manuscript.

R3.2

**Reviewer point:** *"* Although the supplementary material provides useful information on the architecture of the expert committee, the concrete decision rules that lead to the final classification are not presented in a form that can be readily examined or reproduced. In particular, explicit marker thresholds and clearly defined decision paths are not detailed, which makes it difficult to fully assess both the biological plausibility and the stability of the model. Providing representative decision trees or a concise set of decision rules would considerably strengthen the interpretability claims”*."*

We thank the reviewer and agree that the concrete decision rules should be more accessible. We therefore provide exemplary full decision logic for the PUM2 ALPODS committee as a supplementary digital attachment, including marker thresholds and decision paths. We also revised the Supplementary Figure 1 legend to point readers to this material.

The manuscript SI Figure 1 Flow chart for the ALPODS committee assembly was revised to

“A) Flow chart illustrating the assembly of the ALPODS expert committee within FlowXAI….

B) Exemplary decision logic of the ALPODS expert committee for the PUM2 dataset. It should be noted, that we did not meet minimal typical learning sample requirements for HCL or any other level 3 lymphoma (see SI table 1). Explicit marker thresholds and clearly defined decision paths are detailed in a digital attachment PUM2_DecisionLogic.zip.

Abbreviations 1= normal controls, 2= CLL-like, 3=MCL, 7=HCL, 10=all other lymphoma

“

R3.3

**Reviewer point:** *"Tile Mining is a central component of the proposed pipeline, but its formulation would benefit from a more explicit and formal description. "The 'strangeness' metric is introduced conceptually without a precise mathematical definition*, and the normalization and aggregation steps across marker-pair tiles are not fully described*."*

We thank the reviewer and agree that Tile Mining (TM) should be described more explicitly and formally. We have therefore rewritten the TM section so that the pipeline is defined sequentially, including preprocessing, marker-pair generation, tile construction, profile aggregation, strangeness calculation, and thresholding. This clarification was aligned with the corresponding comments of Reviewer 1 and is intended to improve methodological transparency without expanding the main text unnecessarily.

The manuscript is revised to

“After compensation and signed-log transformation, each sample was represented by the concatenated nine-tile summaries of all unique marker pairs. …

For a marker set of size d, let $H_{i}\left( d \right)$ denote the concatenated tile profile of case $i$. Across all marker pairs, this procedure yields

#$H_{i}\left( d \right)=9*\frac{d(d-1)}{2}$.

tiles per sample. For d=11 markers, this corresponds to$\#H\left( d=11 \right)=495$ tiles. For each tile $h\in H(d)$, the density $p(h)$ is defined as the proportion of events falling into that tile:

$$p\left( h \right)=\sum_{e \in E} f_{e},f_{e}=\left\{ \begin{matrix} 1 & e \epsilon h \\ 0 & e\notin h \end{matrix} \right.$$

where E denotes the set of all measured events in the sample. The resulting count matrix is internally normalized ensuring comparability across tiles within each sample.

Let $\overset{ˉ}{T}$ denote the robust reference tile profile estimated from the cohort. TM centers each $H_{i}(d)$ in reation to T by applying robust feature-wise standardization, yielding values $p_{ij}^{*}$. For a given patient i and tube t, the normalized tile values are aggregated to yield a strangeness value:
$\sigma_{i,t}=\frac{100}{p\#H_{i}\left( d \right)}\sum_{j=1}^{\#H_{i}\left( d \right)} p_{ij}^{*}(h)$.

Hence, $\sigma_{i,t}$ summarizes the average robustly standardized deviation of the case from the reference tile profile. Between-sample comparison is therefore performed in a common normalized tile-feature space rather than on identical absolute raw-value borders.”

The manuscript in the methods section was revised to

“All FCS files were processed using the available laboratory compensation information and transformed by signed-log scaling. Because MLL9F and PUM2 were acquired in different laboratories with different antibody panels and tube configurations, the PUM2 analysis was employed as a cross-site benchmark of the FlowXAI framework under heterogeneous conditions. MLL9F was measured using several instruments of the same type.”

R3.4

**Reviewer point:** *"*Furthermore, the method appears to rely on assumptions regarding the distribution of strangeness values and applies thresholding to define atypical samples*.* The supplementary material suggests that related distributions may deviate from normality in certain contexts, reflecting underlying data heterogeneity . In this setting, it would be helpful to provide visualizations of the strangeness distribution and to clarify whether the assumed distributional properties are appropriate*.* A clearer justification of the chosen threshold, supported for instance by sensitivity analyses, would further strengthen this component of the methodology"*"*

We thank the reviewer and agree that the role of the distributional assumption should be stated more carefully. In the revised manuscript, we clarify that the robust Gaussian model is used as a pragmatic thresholding rule for structural atypicality rather than as a claim that strangeness is exactly Gaussian in every cohort. We also specify the study-specific thresholding rule more explicitly and improve the explanation of how the empirical strangeness distribution is interpreted:

“A robust Gaussian reference model was fitted to the empirical strangeness distribution and used as a pragmatic decision rule for structural atypicality. We therefore use the normal model as an empirical thresholding device rather than as an assertion of exact Gaussianity in every dataset. Cases were classified as atypical when their strangeness values fell outside the two-sided limits implied by the chosen central confidence region. For a chosen central confidence level $1-\alpha$, a case was classified as atypical when $\sigma_{i,t}<m-k*sd$ or $\sigma_{i,t}>m+k*sd$, where $M$and $S$ denote the robustly estimated location and scale of the strangeness distribution and $k$ is the corresponding two-sided critical factor.”

To make the thresholding model more transparent, We added visualizations of the empirical strangeness distributions and the corresponding QQ plots in the Supplementary Information.

This directly addresses the reviewer’s request and also supports the between-sample comparability clarification in Reviewer 1 R1.2. The manuscript was revised to

““We provide visualizations of the empirical strangeness distributions for the study cohorts and discuss where the robust Gaussian approximation is adequate and where departures from Gaussian shape likely reflect cohort heterogeneity in SI Strangeness Distributions and Tile Mining Parameters.”

For the justification of the chosen threshold, we refer to our response in R3.6.

R3.5

**Reviewer point:** *"The use of TM to identify atypical samples raises an additional conceptual issue, namely the distinction between technical artefacts and biologically atypical but clinically meaningful cases. The supplementary material indicates that a substantial proportion of atypical samples may correspond to technical issues, although this assessment remains largely qualitative. As a result, there is a potential risk that clinically relevant variability could be inadvertently excluded from the training process "*

We agree and have aligned this point with Reviewer #1 R1.1 and R1.5. TM identifies structural atypicality relative to the reference cohort; it does not infer whether the cause is technical, biological, or both. We would like to emphasize, that the Flow XAI is not intended to replace the diagnostic physician but to present in particular outlier samples or samples classified as „non confident“ in an expert understandable manner (fcs file format) for critical evaluation as explained and illustrated in SI Fig 3, SI Fig 4 and SI Fig 5.

We sought to explain this aspect in the discussion section

“Second, it identifies and visualizes diagnostically relevant cell populations, enabling interactive inspection and explanation of AI-driven decisions.”

We now make explicit that some flagged cases may represent clinically meaningful biological heterogeneity, and that this is precisely why atypical cases were retained for independent evaluation and manual review rather than removed from the full analysis. The existing Results and SI already support this through the manual review of 100 atypical cases and the entity-wise typical/atypical counts. The manuscript was revised to

“TM identifies structural atypicality relative to the reference cohort but does not determine whether the underlying cause is technical, biological, or both. In the present study, TM was therefore used as a human-in-the-loop structural curation step for machine-learning training: atypical cases were excluded only from representative-case selection and supervised training, whereas independent evaluation retained both typical and atypical cases.”

R3.6

**Reviewer point:** *"* Several key parameters, including the number of representative samples, the TM threshold, and aspects of the hierarchical structure, appear to be defined empirically. The use of a reduced training set is an interesting feature of the approach and is supported by results presented in the supplementary material . Nevertheless, additional justification would be valuable to demonstrate that the reported performance is robust across different parameter settings. Even limited sensitivity analyses would help to support this point.*."*

We now state explicitly that these parameters were pragmatic design choices and not biologically fixed constants. Their values and implementation are available in the source code, and the limitation of not exhaustively optimizing them across all settings is now acknowledged.. The discussion of the manuscript was revised to

“The TM threshold and the number of representative training samples were treated as pragmatic design parameters rather than biologically fixed constants. We therefore avoid claims of unique optimality.”

Exemplary, all other used parameters are visible in the source code available at <https://zenodo.org/records/19661905> and can be interactively tested within https://plait.mathematik.uni-marburg.de/.

R3.7

**Reviewer point:** *"*The benchmarking strategy is a positive aspect of the manuscript, notably through the comparison with CITRUS. However, a more explicit positioning of the method relative to a broader set of approaches would help clarify its added value*."*

We thank the reviewer and agree that the benchmarking strategy should be positioned more explicitly. For comparison CITRUS was chosen as stated in the manuscript„because of its conceptual similarity to population-driven immunophenotyping and its previously demonstrated high performance relative to other methods [1, 24].“ The comprehensive literature research which lead to our decision to restrict the benchmarking significantly is explained in detail in SI Methods page 17-19.

In addition, the manuscript was revised to

“Within FlowXAI, the native trustworthiness categories were informative with respect to diagnostic reliability. Because the published comparator systems considered here do not provide directly analogous confident/probable/challenging outputs for the same task (see SI Methods: Literature evaluation for seeking benchmark algorithms), this analysis is presented as a within-framework validation rather than as a cross-algorithm benchmark.”

The results section was revised to:

“To provide a conventional single-tube reference, we benchmarked random forest and multinomial elastic-net classifiers trained on flowFP fingerprints against FlowXAI at hierarchy level 3 across the same 100 repeated train-test splits (Fig. 5). Mean ± SD test MCC values for random forest were 0.79 ± 0.01, 0.72 ± 0.01, and 0.71 ± 0.01 for tubes 1–3, and for glmnet 0.79 ± 0.01, 0.73 ± 0.01, and 0.72 ± 0.01, respectively. The FlowXAI confident stratum reached 0.88 ± 0.01, 0.86 ± 0.01, and 0.81 ± 0.02 across tubes 1–3.”

We added to the discussion:

“These conventional fingerprint-based baselines provide a useful sample-level reference, while FlowXAI’s contribution extends beyond raw classification to hierarchical multi-tube decision support and native trustworthiness assessment.”

And provide Figure 5 in the main manuscript:

“Fig. 5. Benchmarking FlowXAI against conventional single-tube baselines on MLL9F at hierarchy level 3.

Benchmarking was performed using the same 100 repeated class-balanced 80/20 train-test splits as in Fig. 3 (15,594 training samples and 3,899 test samples per repetition). For each tube, sample-level flowFP fingerprints were generated and used as input to a random forest (rf) and a glmnet classifier. MD-plots show the distributions of held-out Matthews correlation coefficient (MCC) values across the 100 test folds for the two baseline classifiers and for FlowXAI stratified by trustworthiness: confident (gold), probable (green), and challenging (blue). Where shown, magenta outlines denote Gaussian fits to the empirical MCC distributions. Mean ± SD MCC values were: ube 1: RF 79% ± 1%, glmnet 79% ± 1%, FlowXAI confident 88% ± 1%, probable 78% ± 3%, challenging 68% ± 5%

tube 2: RF 72% ± 1%, glmnet 73% ± 1%, FlowXAI confident 86% ± 1%, probable 74% ± 3%, challenging 68% ± 3%

tube 3: RF 71% ± 1%, glmnet 72% ± 1%, FlowXAI confident 81% ± 2%, probable 69% ± 3%, challenging 56% ± 6%

Abbreviations: MCC, Matthews correlation coefficient; MD, mirrored density, rf, random forest, multinomial elastic-net, glmnet, rf, random forest”

R3.8

**Reviewer point:** *“*In addition, the performance of the model appears uneven across diagnostic categories. The contingency tables provided in the supplementary material suggest that classification is most effective for common entities such as CLL-like cases, which are also the most represented in the dataset, whereas performance for less frequent entities appears more variable . This observation is understandable but would benefit from a more explicit discussion, particularly regarding the dependence of performance on class prevalence and the implications for clinical use*.”*

Indeed, the performance of differentiation varies between different lymphoma categories as indicated by the confusion matrices. Based on the additional analyses, we no longer consider class prevalence to be the main explanation for the observed differences between lymphoma entities. In particular, the one-vs-rest ROC analyses (SI Figs. 8 and 9) show that HCL is better separated than LPL and MZL, although this pattern is not readily explained by prevalence alone. Moreover, in the additional experiments using the same number of training sample files per class (N = 32; SI Tables 8–11), the relative performance differences between entities persisted. Together, these findings suggest that the dominant factor is biological separability in the measured immunophenotypic space and, for some entities, the limited discriminatory information provided by the available antibody panel. This interpretation is consistent with the structural-embodiment analysis shown in Figure 1, where some lymphoma entities formed clearer structures than others.

We therefore revised the Discussion to state more explicitly that performance differences should not be interpreted primarily as a consequence of prevalence, but rather as reflecting how well a given entity is resolved by the measured marker set. From a clinical perspective, this means that FlowXAI is likely to be most reliable for **immunophenotypically distinct entities,** whereas more overlapping entities should be interpreted with greater caution and in conjunction with morphology, molecular data, and expert review.

“Uneven performance across lymphoma entities does not appear to be explained primarily by class prevalence. In the one-vs-rest ROC analyses, HCL showed better separability than LPL and MZL (SI Figs. 8 and 9), and in additional experiments using the same number of training sample files per class (N = 32; SI Tables 8–11), the relative performance differences between entities persisted. Together, these findings indicate that entity-specific performance is driven mainly by the biological separability of lymphoma entities in the measured immunophenotypic space and by the discriminatory information content of the available antibody panel. This interpretation is consistent with the structural-embodiment analysis in Figure 1, which showed that some lymphoma entities form clearer structures than others. Clinically, these results suggest that FlowXAI is most reliable for immunophenotypically distinct entities, whereas more overlapping categories require more cautious interpretation and continued integration with morphology, molecular findings, and expert review.”

R3.9

**Reviewer point: “**Closely related to this point, the performance of the approach appears to depend on the information content of the antibody panel. The supplementary material indicates that some lymphoma entities are not clearly separable given the available markers. This suggests that the limitations of the model may partly reflect intrinsic biological constraints rather than purely algorithmic factors. A more explicit discussion of the relationship between panel design, biological separability, and model performance would provide important context for interpreting the results and assessing their generalizability.”

We agree that panel design, biological separability, and model performance are closely linked. We revised the Discussion to state explicitly that the panels used here reflect routine diagnostic practice rather than an optimized marker set for maximal separation of all lymphoma entities. Some distinctions are not expected to be fully resolvable by surface immunophenotyping alone, even for expert interpretation. Consistent with Fig. 1 and the selective one-vs-rest analyses, CLL-like cases and HCL showed comparatively clearer separability, whereas several other entities showed greater overlap. Thus, part of the observed performance ceiling is panel- and biology-related rather than purely algorithmic, and generalizability should be interpreted relative to the discriminatory information contained in the panel used. We have additionally added the following paragraph to the Introduction section:

“Exact labels of disease entities are highly desirable for patient care and follow internationally accepted classifications involving morphology, immunophenotype and genetics. They evolve over time as biological knowledge expands and inevitably incorporate expert judgment. Accordingly, recommendations for the optimal diagnostic panel of markers to optimally dissect different B-cell lymphomas still under investigation [14-16]. In clinical routine, MFC represents an essential and, in selected entities such as chronic lymphocytic leukemia (CLL) and hairy cell leukemia (HCL), often sufficient diagnostic modality [17, 18]. However, for the majority of B-cell lymphomas, definitive diagnosis requires integration of histopathological findings from lymph node or tissue biopsies, which remain the diagnostic gold standard[19]. Importantly, according to current standards, some lymphoma entities cannot be reliably diagnosed by flow cytometry alone, irrespective of the specific antibody panel used. [20]. However, an assignment of B-cell lymphoma without subtyping is frequently possible and a "best guess" for assignment of a subtype can be attempted.

“

R3.10

**Reviewer point:** *“* The validation strategy includes repeated train/test splits and the use of an additional dataset, which are both strengths. The inclusion of a dataset acquired with a different panel is particularly valuable, as it provides a stringent test of robustness across heterogeneous settings. At the same time, differences in panel composition and training strategy make it more difficult to isolate the respective contributions of the model and the experimental configuration. A complementary evaluation using a fixed trained model applied to an independent cohort would further strengthen the assessment of generalizability.*.”*

We thank the reviewer and agree that a fixed-model evaluation on an independent matched-panel cohort would further strengthen the assessment of transportability. In the present study, however, the PUM2 experiment was designed to address a different question, namely whether the FlowXAI framework remains informative under cross-site heterogeneity in antibody panel and tube configuration. We therefore revised the manuscript to describe PUM2 more explicitly as a cross-site benchmark under heterogeneous conditions and to avoid overstating what this experiment demonstrates. We now state more clearly that a fixed-model matched-panel evaluation would represent an important next step beyond the scope of the present revision.

The manuscript methods section was revised to

“Because MLL9F and PUM2 were acquired in different laboratories with different antibody panels and tube configurations, the PUM2 analysis was designed as a cross-site benchmark of the FlowXAI framework under heterogeneous conditions. “

And the results section to:

“A fixed-model evaluation on an independent matched-panel cohort would further strengthen transportability assessment and remains an important next step beyond the scope of the current work.”

R3.11

**Reviewer point:** *“*In addition, TM appears to be computed prior to data splitting; although this step is unsupervised, recomputing it within each training fold could be considered to further reinforce methodological rigor.”

We agree that fold-wise recomputation of TM would be methodologically cleaner. In the current study, TM was applied once globally as an unsupervised preprocessing step and used only for training-sample curation. We will now state this more explicitly and discuss it as a limitation. The current Methods already disclose the global unsupervised TM step.

The manuscript was refined to

“TM was applied as an unsupervised preprocessing step before repeated train/test splitting and was used exclusively for training-sample curation. We now acknowledge that fold-wise recomputation would represent a stricter design and the current implementation is acknowledged as a methodological limitation.”

R3.12

**Reviewer point: “**The manuscript reports global performance metrics such as accuracy and MCC, and the supplementary material provides detailed contingency tables stratified by confidence levels, which is a clear strength . Nevertheless, the evaluation could be further enriched by incorporating clinically oriented metrics such as predictive values and by examining the calibration of the confidence scores. Such analyses would enhance the clinical interpretability of the results*.”*

We agree that clinically oriented metrics improve interpretability. We added diagnosis-specific one-vs-rest operating-point metrics at the three FlowXAI trustworthiness categories and now report PPV, NPV, sensitivity/recall, and specificity, together with selective ROC and PR curves. We also added calibration analysis of the trustworthiness output using reliability plots, ECE, and Brier score. These additions allow readers to assess how FlowXAI’s confidence categories relate to clinically meaningful predictive values and observed correctness.

We propose a new subparagraph in the revised manuscript to the results section:

“Trustworthiness validation.“

FlowXAI’s trustworthiness output was informative with respect to diagnostic reliability [1, 2]. The observed correctness rate increased from 79.5% (95% CI 79.3% to 79.8%) for challenging cases to 86.9% (95% CI 86.6% to 87.1%) for probable cases and 94.3% (95% CI 94.2% to 94.4%) for confident cases. Overall, calibration of predicted correctness showed low average miscalibration (ECE 0.024; Brier score 0.089). In the upper reliability bins, calibrated predicted correctness ranged from 90.1% to 90.6%, whereas observed correctness ranged from 93.5% to 94.0%, indicating conservative calibration in the highest-confidence region.

Selective one-vs-rest ROC and precision-recall analyses based on the ordered FlowXAI trust strata showed strongest selective discrimination for NC, CLL-like, and HCL, intermediate performance for MZL, and limited selective utility for MCL, FL, and LPL (Supplementary Selective one-vs-rest ROC and precision-recall analysis from FlowXAI categories of trustworthiness Figs. 7–8).

And

“Selective one-vs-rest ROC and precision-recall analyses based on the ordered FlowXAI trust strata showed strongest selective discrimination for NC, CLL-like, and HCL, intermediate performance for MZL, and limited selective utility for MCL, FL, and LPL (Supplementary Selective one-vs-rest ROC and precision-recall analysis from FlowXAI categories of trustworthiness Figs. 7–8).”

And to the discussion:

“The trustworthiness validation supports the clinical usefulness of FlowXAI’s internal trustworthiness stratification. Lower-trust cases carried a substantially higher error risk, whereas confident cases were associated with markedly higher observed diagnostic reliability. Selective one-vs-rest ROC and precision-recall analyses indicate that FlowXAI trustworthiness categories have entity-specific clinical value: high-trust predictions are most actionable for NC, CLL-like cases, and HCL; MZL shows intermediate decision-support utility; and MCL, FL, and LPL remain predominantly expert-review entities.

“

R3.13

**Reviewer point:** *"* Finally, the positioning of the system within clinical practice would benefit from clarification. While the manuscript sometimes presents FlowXAI as a diagnostic system, the results and supplementary material indicate that certain diagnostic decisions still require additional information, including morphology or molecular data . Framing the system more explicitly as a decision-support or triage tool would align its description with its demonstrated capabilities.”

Indeed, the reviewers raises another important issue which is the discrepancy between comprehensive studies of academic centers and real-world diagnostic procedures for lymphoma definition. We therefore repeatedly refer to the new WHO lymphoma classification which integrates information from different diagnostic methods in order to optimize separation of classes. In clinical practice diagnosis relies on morphology and immunophenotyping and molecular genetic analysis are only performed in case of diagnostic or therapeutic relevance such as TP53 mutational status in B-CLL and if available.

We stated in the conclusion at the end of the discussion “The system is self-explanatory, knowledge-based by design, explicitly integrates clinical diagnostic priorities while respecting the limitations of immunophenotypic data and its self-assessment capabilities facilitate a transparent and interactive mode with human experts”

Now we positioned FlowXAI within clinical practice more clearly. The intention of the present work is not to propose an autonomous diagnostic system, but a decision-support and triage tool that assists expert interpretation of flow cytometry data.

To the manuscript conclusion the sentence was added:

“FlowXAI is intended as a decision-support and triage tool for expert users, rather than as a fully autonomous diagnostic system. Diagnostic integration with morphology, molecular findings, and expert review remains necessary for specific clinical distinctions.”

R3.14

**Reviewer point:** *"The description of the cohorts could be expanded to include more detailed demographic and clinical characteristics."*

We thank the reviewer and agree that cohort description should be as transparent as possible. Unfortunately, this study uses MLL9F data published elsewhere for which no more details are accessible. In addition, demographic or clinical metadata for PUM2 were not available in the retrospective source datasets. The supplementary states this now explicitly in supplementary information table 3:

“The MLL9F and PUM2 datasets were retrospective, anonymized flow-cytometry datasets derived from routine diagnostic workflows. The available metadata comprised the sample identifier, diagnostic reference label, antibody panel/tube information, and the corresponding FCS-derived measurements used for analysis. Additional patient-level demographic and clinical variables, including age, sex, disease stage, treatment status, blood counts, morphology, cytogenetic or molecular findings, and clinical follow-up, were not available to us in a sufficiently complete and standardized form for analysis.”

R3.15

**Reviewer point:** *"The statistical methods used for comparisons are not always explicitly specified and would benefit from clarification."*

We revised the Methods section to specify the validation design, the performance metrics, and the way comparisons between models and trustworthiness strata were summarized. In particular, we now state explicitly that model performance was evaluated on held-out test samples from 100 repeated class-balanced 80/20 train–test splits, that multiclass performance was primarily summarized using MCC, and that additional analyses used accuracy, PPV, NPV, sensitivity/recall, specificity, ROC/PR curves, calibration plots, ECE, and Brier score where appropriate.

R3.16

**Reviewer point:** *"* Several minor typographical and language issues are present throughout the manuscript. For example, expressions such as "NC-associated structure" and "self-competence" are unclear or appear to be used in a non-standard way in this context. In addition, capitalization is sometimes inconsistent (for instance, alternating between "Flow Cytometry" and "flow cytometry"). A careful linguistic revision would improve overall clarity and readability. More generally, some sentences—particularly in the Methods section—are syntactically dense or ambiguous, which may hinder comprehension for readers*."*

We thank the reviewer for the careful language review. We revised the manuscript for terminology, capitalization, and readability, including consistent use of “flow cytometry,” “FlowXAI” and related technical terms.

R3.17

**Reviewer point:** *"* The figures are generally informative, and the supplementary material provides valuable visual support. However, several aspects could be improved to enhance readability and interpretability. In particular, some figure legends lack sufficient detail, and the meaning of color scales is not always immediately clear. Axis labels are occasionally ambiguous, and certain figures require substantial prior knowledge to be fully interpreted.”

We improved figure readability and clinical interpretation of Figure 1-4 in the revised manuscript, especially figure 1 was revised to

“…The elevation ranges are mapped to high-dimensional data distances and densities of the projected points. The exact mapping is defined in [65]. …

A Self-organization of a subset of the MLL9F dataset (3,000 NC, 3,000 CLL-like, 3,000 non-CLL-like B-NHL samples) reveals two dominant valleys that contain the clusters in SI table 1, two outlier-associated structures represented as outlier groups in the same table, and isolated atypical samples depicted as various outliers in above referenced table.

B The same topographic map highlights a pronounced structural separation between normal controls (NC) and B-NHL samples (see SI Tab 1 for contingency table.). Without TM incorporation, many single outliers scatter across the topographic map, illustrated as single dots in single valleys.

C After application of Tile Mining (TM) for elimination of structurally atypical samples, the topographic map representing the self-organization of lymphoma cases reveals three dominant valleys that contain the clusters in SI table 2 and markedly fewer isolated outliers.

D Mapping of clinical labels onto the TM-filtered topographic map shows correspondence of major structures with CLL-like entities, HCL, and all other lymphomas (AOL), while some entities remain not clearly separable (see SI Table 2 for contingency table).”

R3.18

**Reviewer point:** *"* The manuscript would benefit from including more explicit visualizations of the decision logic, such as simplified decision trees or representative rule paths. A schematic representation of the confidence categories described in the text would also improve clarity. In addition, a visualization of the strangeness distribution would be particularly relevant given its central role in the methodology. Finally, some supplementary figures are difficult to read at standard resolution and could benefit from improved graphical quality*."*

We thank the reviewer and agree that the visual presentation of the workflow should guide readers more directly. Our proposition is a traceable workflow that can be inspected by the user as stated in the manuscript:

“We have created a live, interactive demonstration of our FlowXAI platform at <https://plait.mathematik.uni-marburg.de/>. This online portal is specifically designed so that physicians, computational scientists, and readers can explore our proposed step-by-step classification workflow: Visitors can upload data (or select from example datasets) and see how FlowXAI automatically identifies relevant cell populations, performs classification, and provides self-explaining visual outputs. Each FlowXAI decision is grounded in traceable rules that mimic the logic of human experts. FlowXAI provides these understandable explanations stored in the FCS format. “

Rather than exhaustively visualizing all internal rule combinations, we improved the explanatory value of the existing workflow figures and supplementary material, added strangeness-distribution visualizations, and provided source code and exemplary decision logic as supplementary material for readers who want to inspect the full rule logic.

Upon checking within the submission system, the high-resolution figures have to be downloaded by clicking on the link within the pdf, every figure is now provided separately but the resolution shown in the pdf remains low.

1. Van Calster B, McLernon DJ, Van Smeden M, Wynants L, Steyerberg EW. Calibration: the Achilles heel of predictive analytics. BMC medicine. 2019;17(1):230.

2. Niculescu-Mizil A, Caruana R. Predicting good probabilities with supervised learning. Proceedings of the 22nd international conference on Machine learning2005. p. 625-32.

3. Thrun MC, Ultsch A. Swarm Intelligence for Self-Organized Clustering. Artificial Intelligence. 2021;290:103237. doi: 10.1016/j.artint.2020.103237.

4. Thrun MC, Ultsch A. Uncovering High-Dimensional Structures of Projections from Dimensionality Reduction Methods. MethodsX. 2020;7:101093. doi: 10.1016/j.mex.2020.101093.

5. Ultsch A. Maps for the visualization of high-dimensional data spaces. Workshop on Self organizing Maps (WSOM). Kyushu, Japan2003. p. 225-30.

6. Thrun MC, Pape F, Ultsch A. Conventional Displays of Structures in Data Compared With Interactive Projection-Based Clustering (IPBC). International Journal of Data Science and Analytics. 2021;12(3):249-71. doi: 10.1007/s41060-021-00264-2.

7. Ultsch A, Lötsch J. Machine-learned cluster identification in high-dimensional data. Journal of Biomedical Informatics. 2017;66(C):95-104.

8. Thrun MC. Improving the Sensitivity of Statistical Testing for Clusterability with Mirrored-Density Plot. In: Archambault D, Nabney I, Peltonen J, editors. Machine Learning Methods in Visualisation for Big Data. Norrköping , Sweden: The Eurographics Association; 2020.

9. Adolfsson A, Ackerman M, Brownstein NC. To cluster, or not to cluster: An analysis of clusterability methods. Pattern Recognition. 2019;88:13-26.

10. Stier Q, Thrun MC. An efficient multicore CPU implementation of the DatabionicSwarm. International Federation of Classification Societies (IFCS). San José, Costa Rica2024. p. 1-8.

11. Zhao M, Mallesh N, Höllein A, Schabath R, Haferlach C, Haferlach T, et al. Hematologist‐Level Classification of Mature B‐Cell Neoplasm Using Deep Learning on Multiparameter Flow Cytometry Data. Cytometry Part A. 2020;97:1073-80. doi: 10.1002/cyto.a.24159.

12. Bishop CM. Pattern recognition. Mach Learn. Singapore: Springer; 2006.

13. Goodfellow I, Bengio Y, Courville A. Deep learning. Cambridge, Massachusetts, USA: MIT press; 2016.

14. Hoffmann J, Rother M, Kaiser U, Thrun MC, Wilhelm C, Gruen A, et al. Determination of CD43 and CD200 surface expression improves accuracy of B-cell lymphoma immunophenotyping. Cytometry Part B: Clinical Cytometry. 2020;98(6):476-82. doi: 10.1002/cyto.b.21936.

15. Van Dongen J, Lhermitte L, Böttcher S, Almeida J, Van Der Velden V, Flores-Montero J, et al. EuroFlow antibody panels for standardized n-dimensional flow cytometric immunophenotyping of normal, reactive and malignant leukocytes. Leukemia. 2012;26(9):1908-75.

16. Rawstron AC, Kreuzer KA, Soosapilla A, Spacek M, Stehlikova O, Gambell P, et al. Reproducible diagnosis of chronic lymphocytic leukemia by flow cytometry: An European Research Initiative on CLL (ERIC) & European Society for Clinical Cell Analysis (ESCCA) Harmonisation project. Cytometry Part B: Clinical Cytometry. 2018;94(1):121-8.

17. Shadman M. Diagnosis and treatment of chronic lymphocytic leukemia: a review. Jama. 2023;329(11):918-33.

18. Falini B, Tiacci E. Hairy-cell leukemia. New England Journal of Medicine. 2024;391(14):1328-41.

19. Kroft SH, Sever CE, Bagg A, Billman B, Diefenbach C, Dorfman DM, et al. Laboratory workup of lymphoma in adults: Guideline from the American Society for Clinical Pathology and the College of American Pathologists. American Journal of Clinical Pathology. 2021;155(1):12-37.

20. Ehinger M, Béné MC. Morphology and multiparameter flow cytometry combined for integrated lymphoma diagnosis on small volume samples: possibilities and limitations. Virchows Archiv. 2024;485(4):591-604.

We hope that the revision now meets the criteria for publication in PLOS Medicine. We are very much looking forward to hearing from you.

Yours sincerely,

Cornelia Brendel and Michael Thrun (on behalf of the other authors)
