# Supplementary material for: Self-explaining artificial intelligence for the classification of B cell non-Hodgkin lymphoma: A diagnostic decision support study
Source: PLoS Med. 2026 Jul 13;23(7):e1004889. doi: 10.1371/journal.pmed.1004889 (PMC13421771; doi:10.1371/journal.pmed.1004889)
Supplement: Attachment — Submitted filename: response_to_reviewer3_second_round_V4.docx [file pmed.1004889.s008.docx]

Dear Editor-in-Chief, dear Associate Editor Dr. Starowicz,

Thank you for handling our manuscript, “Self-explaining artificial intelligence for the classification of B cell non-Hodgkin lymphoma” (PMEDICINE-D-25-04525R2), and for giving us the opportunity to address the remaining comments from Reviewer 3.

We are grateful that Reviewers 1 and 2 had no further major concerns and that Reviewer 3 acknowledged the substantial improvement of the manuscript. We have now addressed the remaining points raised by Reviewer 3 in a targeted manner marked in red.

Reviewer #3:

“I thank the authors for their careful revision of the manuscript and for the detailed responses provided. The manuscript has improved compared to the initial submission, particularly with respect to clarity of presentation and the documentation of key methodological components.

The description of the Tile Mining (TM) procedure is now more explicit, with the addition of more structured explanations in the main text and in the supplementary material. The clarification of its intended role as a sample-level structural filtering step is helpful. The authors have also expanded the description of the ALPODS framework and provided additional material regarding the organization of the expert committee, which contributes to a better understanding of the overall approach.

That being said, several points would still benefit from further clarification or more explicit “

R3.1

**Reviewer point: “**The interpretability of the ALPODS framework, although improved, remains somewhat indirect. While additional material is now available, the decision logic is not yet presented in a concise and directly accessible form within the main manuscript. In particular, the exact nature of the individual "experts" remains somewhat implicit. It is not entirely clear to what extent these components correspond to explicit rule-based systems, learned classifiers, or hybrid decision modules. Clarifying this point, and providing representative decision paths or simplified rule sets, would facilitate a more direct evaluation of the interpretability claims.”

We are delighted about the precise and well-founded feedback reflections and remarks regarding our adjusted second manuscript version.

We particularly thank reviewer 3 for the additional suggestions and agree that the ALPODS decision logic should be more directly accessible. We revised the Methods to define an ALPODS expert as a data-derived, rule-based decision module: relevant cell populations and marker conditions are learned from training data, while the output remains explicit marker-threshold logic. We also provide supplementary digital PUM2 decision-logic files for the tube- and task-specific experts, so representative rule logic can be inspected without relying on the online portal.

The manuscript was revised to

“This supervised component consists of tube- and task-specific ALPODS experts, an extension of the original ALPODS algorithm [29]. In this context, an ‘expert’ denotes a data-derived rule-based decision module. Each expert is trained for a specific tube and diagnostic task. During training, ALPODS identifies diagnostically informative cell subpopulations, derives rules for these populations, and returns a task-specific output. The relevant cell populations are defined by rules learned from a representative sample of the training data. The rules are composed of conditions which recursively partition the data set on the most informative features at each level. The resulting decision support logic can be expressed as explicit marker-threshold logic.

To make the decision support logic inspectable, we provide exported PUM2 decision logic as supplementary digital material. The provided rule files represent the tube 1 and tube 2 experts. Each rule file assigns samples to rule-defined populations and diagnostic outputs through explicit marker conditions. The accompanying subject-level population-frequency files link the rule logic to case-level explanatory outputs. Full algorithmic details are provided in the Supplementary Methods, SI Figure 1, the supplementary digital rule files.”

R3.2

**Reviewer point: “**Regarding the TM procedure, the formalization is clearer than in the initial version. However, the thresholding strategy remains primarily empirically motivated. While the authors provide a rationale for the use of a Gaussian-based rule, the sensitivity of the results to this choice is not explored in detail. A brief discussion of this aspect would help to better contextualize the robustness of the method. More generally, the TM step appears to capture distributional structures of cell populations across marker pairs, which can be seen as an implicit representation of population-level organization. It would be helpful to clarify how stable these representations are across datasets and parameter settings.”

We added a short Discussion statement clarifying that the TM threshold is cohort-specific and should not be interpreted as a universal biological cutoff. We also state that stability may depend on cohort composition, preprocessing, antibody panel, and tube configuration.

**The manuscript was revised to**

“Because the TM threshold is empirically estimated from cohort-specific strangeness distributions, its numerical value should not be interpreted as a universal biological cutoff. Rather, TM provides a representation of population-level organization within the measured marker-pair space, while the exact threshold may vary with cohort composition, preprocessing, antibody panel, and tube configuration. Future work should therefore evaluate TM threshold sensitivity prospectively and across matched-panel datasets.”

R3.3

**Reviewer point:** “The clarification that TM does not distinguish between technical artefacts and biologically atypical samples is useful. At the same time, this highlights a limitation that may affect the representativeness of the training data, particularly for rare or heterogeneous entities. This point would benefit from being more explicitly acknowledged in the discussion.”

This aspect is indeed one the major challenges of our approach, because even for the human expert this discrimination in often challenging. To the best of our knowledge physicians need at least one year of supervised training to rule out misinterpretation either due to technical or biological artifacts. In routine diagnostic laboratories repeated staining and measurement of peculiar (outlier) samples from a reserve blood sample is a common procedure. However, we believe that similar to human learning discrimination of clearly degraded samples or samples with inherent biological deviation will improve with the number of training samples and feedback from the human expert. We sought to point to this limitation more explicit and revised the discussion section:

„

TM defines structural atypicality relative to the reference cohort and the measured antibody-panel space; it cannot determine whether an atypical profile reflects a technical artefact, genuine biological variation, or insufficiently represented lymphoma phenotype. Consequently, excluding TM-atypical cases from representative-case selection and supervised training may stabilize learning for common phenotypes but may also reduce the representation of heterogeneous entities in the learned decision rules. For this reason, TM should be interpreted as a human-in-the-loop curation and flagging step rather than as an autonomous exclusion criterion: atypical cases require expert review and, in the present study, were excluded only from representative-case selection and supervised training, whereas evaluation retained both typical and atypical cases “

R3.4

**Reviewer point:** “The validation framework has been clarified, and the distinction between cross-site benchmarking and strict external validation is now appropriately stated. This is a positive point. However, this also indicates that a full assessment of model generalizability on an independent cohort with identical predictors has not yet been performed, which could be more clearly discussed as a limitation.”

We thank the reviewer and agree. We revised the limitation statement to specify that strict fixed-model external validation would require an independent cohort acquired with identical or matched predictors.

**The manuscript was revised by precising and then moving the following sentence:**

“A fixed-model evaluation on an independent cohort acquired with an identical or matched antibody panel would further strengthen assessment of model generalizability and remains an important next step beyond the scope of the current work.”

R3.5

**Reviewer point:** “The additional analyses provided by the authors confirm that performance is not uniform across diagnostic categories. As reported in the revised manuscript, classification appears strongest for entities such as normal controls, CLL-like cases, and HCL, while remaining more limited for others such as MCL, FL, and LPL. This heterogeneity is clinically relevant and would merit a more prominent discussion in the main text, as it directly affects the potential scope of application of the method. More generally, the performance of the approach appears to depend on the information content of the antibody panel. The supplementary material suggests that some entities are not clearly separable given the available markers. This indicates that part of the limitation may be related to intrinsic biological separability rather than algorithmic performance alone. A clearer discussion of this dependency would provide useful context for interpreting the results.”

The reviewer points to one of the most central aspects of the approach of automated B-cell immunophenotyping. Human immunophenotyping experts report CLL-linke cases and HCL with stronger evidence but even for these diagnoses they sometimes state „compatible with CLL“. MCL, FL and LPL are mostly reported as „proof of B-NHL“ without further specification. The fact that some lymphoma entities such as LPL and MZL cluster together and are therefore not always clearly distinguishable have also been described by others (Zhao Cytometry A 2020, Van der Velden, Blood 2014 and Bassarova et al., Am J Clin Path 2015). In awareness of this problem we dedicated the first chapter to this particular problem trying to highlight the mathematical ground truth within the diagnostic data. In the discussion section we explained that additional „comprehensive clinical evidence, including genetic and histopathological information“ is required for the correct diagnosis of some lymphoma. We further stated that

„In the present study, these analyses revealed a clear separation between normal controls and lymphoma samples, as well as distinct structures corresponding to CLL-like entities and hairy cell leukemia (HCL). However, other lymphoma entities were not clearly separable when analysis was restricted to the specific antibody panels used. Importantly, the absence of a distinct structure in this setting does not imply the absence of a valid disease entity; rather, it reflects the limited discriminatory power of the selected immunophenotypic markers. ~~Whether the inclusion of additional markers would allow finer resolution of WHO-defined entities remains an open question.~~“

We now additionally explain that

„It has been shown that addition of antigens in a given diagnostic panel may enhance the separability for certain lymphoma subtypes such as CD43, CD200 and ROR1 for B-CLL [1-5], or transferrin receptor in high grade lymphomas[6]. However, an optimal antibody panel for B-cell immunophenotyping has not been defined yet [3] but resolution could be enhanced with identification of further discriminatory antigens in future [1].”

…

“Accordingly, reduced performance for some entities should not be interpreted solely as algorithmic failure, but also as a consequence of limited panel information content and intrinsic immunophenotypic overlap between biologically related lymphoma entities.”

R3.7

**Reviewer point:** “The manuscript refers to an online platform for interactive exploration of the model. Access to this resource requires user registration, which prevented its evaluation in the context of this review. While such a tool may be useful, it would be important to ensure that all essential elements required for understanding and assessing the method remain fully accessible within the manuscript and supplementary material.”

We thank the reviewer for noting this access issue. We revised the manuscript to clarify that the online portal is optional. The workflow, model architecture, representative explanations, decision logic, and implementation details are provided in the manuscript, Supplementary Information, supplementary digital files, and reviewer-accessible source code.

**The manuscript was revised to**

“…which is intended as an optional interactive demonstration.”

R3.8

**Reviewer point:** “Finally, while the manuscript reports appropriate global performance metrics, the inclusion of additional clinically oriented metrics or calibration analyses could further improve the interpretability of the results.”

We thank the reviewer. This point has been addressed in the first revision by adding trustworthiness-validation analyses and selective one-vs-rest ROC/precision-recall analyses. The revised results reported observed correctness rates with 95% confidence intervals, expected calibration error, Brier score, and entity-specific selective performance across the trustworthiness categories.

**We kindly point the reviewer to the section:**

“FlowXAI’s trustworthiness output was informative with respect to diagnostic reliability [7, 8]. The observed correctness rate increased from 79.5% (95% CI 79.3% to 79.8%) for challenging cases to 86.9% (95% CI 86.6% to 87.1%) for probable cases and 94.3% (95% CI 94.2% to 94.4%) for confident cases. Overall, calibration of predicted correctness showed low average miscalibration (ECE 0.024; Brier score 0.089). In the upper reliability bins, calibrated predicted correctness ranged from 90.1% to 90.6%, whereas observed correctness ranged from 93.5% to 94.0%, indicating conservative calibration in the highest-confidence region.

Selective one-vs-rest ROC and precision-recall analyses based on the ordered FlowXAI trust strata showed strongest selective discrimination for NC, CLL-like, and HCL, intermediate performance for MZL, and limited selective utility for MCL, FL, and LPL (Supplementary Selective one-vs-rest ROC and precision-recall analysis from FlowXAI categories of trustworthiness Figs. 7–8).”

**Reviewer concluding comment:**

“The manuscript has improved following revision and now presents a coherent and potentially useful approach. The remaining points mainly concern clarification, accessibility, and positioning of the results rather than fundamental methodological issues. Addressing these aspects would further strengthen the clarity and clinical relevance of the work.”

We thank the reviewer for this positive overall assessment. We believe the targeted revisions above further improve clarity, accessibility, and clinical positioning while preserving the core manuscript structure of the revised submission.

1.	Rawstron, A., et al., *Improving efficiency and sensitivity: European Research Initiative in CLL (ERIC) update on the international harmonised approach for flow cytometric residual disease monitoring in CLL.* Leukemia, 2013. **27**(1): p. 142-149.

2.	Fan, L., et al., *Expression patterns of CD200 and CD148 in leukemic B-cell chronic lymphoproliferative disorders and their potential value in differential diagnosis.* Leukemia & lymphoma, 2015. **56**(12): p. 3329-3335.

3.	Rawstron, A.C., et al., *Reproducible diagnosis of chronic lymphocytic leukemia by flow cytometry: An European Research Initiative on CLL (ERIC) & European Society for Clinical Cell Analysis (ESCCA) Harmonisation project.* Cytometry Part B: Clinical Cytometry, 2018. **94**(1): p. 121-128.

4.	Hoffmann, J., et al., *Determination of CD43 and CD200 surface expression improves accuracy of B-cell lymphoma immunophenotyping.* Cytometry Part B: Clinical Cytometry, 2020. **98**(6): p. 476-482.

5.	Uhrmacher, S., et al., *Use of the receptor tyrosine kinase-like orphan receptor 1 (ROR1) as a diagnostic tool in chronic lymphocytic leukemia (CLL).* Leukemia research, 2011. **35**(10): p. 1360-1366.

6.	Esserman, L., et al., *An epitope of the transferrin receptor is exposed on the cell surface of high-grade but not low-grade human lymphomas.* 1989.

7.	Van Calster, B., et al., *Calibration: the Achilles heel of predictive analytics.* BMC medicine, 2019. **17**(1): p. 230.

8.	Niculescu-Mizil, A. and R. Caruana. *Predicting good probabilities with supervised learning*. in *Proceedings of the 22nd international conference on Machine learning*. 2005.

We hope that the revised manuscript and response adequately address the remaining concerns and are grateful for your continued consideration of our work for publication in PLOS Medicine.

Sincerely,

Michael C. Thrun

on behalf of all authors
