# Supplementary material for: Self-explaining artificial intelligence for the classification of B cell non-Hodgkin lymphoma: A diagnostic decision support study
Source: PLoS Med. 2026 Jul 13;23(7):e1004889. doi: 10.1371/journal.pmed.1004889 (PMC13421771; doi:10.1371/journal.pmed.1004889)
Supplement: Peer review history

# Editor report: Decision Letter 0

**DOI:** 10.1371/journal.pmed.1004889.r001

**Authors:** Heather Van Epps

30 Dec 2025

Dear Dr Thrun,

Thank you for submitting your manuscript entitled "Self-explaining Artificial Intelligence for the Classification of B cell Non-Hodgkin Lymphoma" for consideration by PLOS Medicine.

Your manuscript has now been evaluated by the PLOS Medicine editorial staff, and I am writing to let you know that we would like to send your submission out for external peer review.

However, before we can send your manuscript to reviewers, we need you to complete your submission by providing the metadata that is required for full assessment. To this end, please login to Editorial Manager where you will find the paper in the 'Submissions Needing Revisions' folder on your homepage. Please click 'Revise Submission' from the Action Links and complete all additional questions in the submission questionnaire.

For clinical studies, please upload a copy of your trial study protocol as a supporting information file. The study protocol should be the version submitted for approval to the institutional review board or ethics committee, should include any amendments to the study protocol, as well as the date of their approval by the institutional review or ethics committee. Please also detail any deviations from the study protocol in the Methods section of your manuscript. The editors will consider the protocol and study conduct prior to a final decision for external review.

Please re-submit your manuscript within two working days, i.e. by Jan 1st. However, in view of the holidays, please just resubmit the paper at your earliest convenience.

Login to Editorial Manager here: https://www.editorialmanager.com/pmedicine

Once your full submission is complete, your paper will undergo a series of checks in preparation for peer review. Once your manuscript has passed all checks it will be sent out for review.

Feel free to email us at plosmedicine@plos.org if you have any queries relating to your submission.

Kind regards,

Heather Van Epps, PhD

Consulting Editor

PLOS Medicine

# Peer review: Decision Letter 1

**DOI:** 10.1371/journal.pmed.1004889.r002

**Authors:** Katarzyna Starowicz

1 Apr 2026

Dear Dr Thrun,

Many thanks for submitting your manuscript "Self-explaining Artificial Intelligence for the Classification of B cell Non-Hodgkin Lymphoma" (PMEDICINE-D-25-04525R1) to PLOS Medicine. Please accept my sincere apologies for delay in decision regarding your manuscript. The paper has been reviewed by subject experts and a statistician; their comments are included below and can also be accessed here: [LINK]

After discussing the paper with the editorial team and an academic editor with relevant expertise, I'm pleased to invite you to revise the paper in response to the reviewers' comments.

As you will see, the reviewers find your study of interest however all reviewers are pointing out several areas for revision: more information on the validation step (statistical/validation part needs to be more robust), comparison to other existing methods, greater clarity in presentation and explanation of how it would be incorporated into clinical workflows and highlighting the clinical relevance, and additionally a better understanding of whether it can identify rare B cell entities.

We would be interested in considering the revised manuscript that addresses all of the reviewers' concerns, but we cannot provide any guarantees at this stage regarding publication.

In addition to these revisions, you may need to complete some formatting changes, which you will receive in a follow up email. A member of our team will be in touch with a set of requests shortly. If you do not receive a separate email within a few days, please assume that checks have been completed, and no additional changes are required.

When you upload your revision, please include a point-by-point response that addresses all of the reviewer and editorial points, indicating the changes made in the manuscript and either an excerpt of the revised text or the location (eg: page and line number) where each change can be found. Please also be sure to check the general editorial comments at the end of this letter and include these in your point-by-point response. When you resubmit your paper, please include a clean version of the paper as the main article file and a version with changes tracked as a marked-up manuscript. It may also be helpful to check the guidelines for revised papers at http://journals.plos.org/plosmedicine/s/revising-your-manuscript for any that apply to your paper.

We ask that you submit your revision by Apr 22 2026 11:59PM. However, if this deadline is not feasible, please contact me by email, and we can discuss a suitable alternative.

Don't hesitate to contact me directly with any questions (kstarowicz@plos.org).

Best regards,

Katarzyna

Katarzyna Starowicz, PhD

Associate Editor

PLOS Medicine

kstarowicz@plos.org

-----------------------------------------------------------

Comments from the reviewers:

Reviewer #1: This manuscript presents FlowXAI, an AI system for B‑NHL classification from flow data. The topic is important and the dataset is sizable however several key methodological details are unclear and the presentation is difficult to follow.

Major Issues

1. The purpose and advantages of TM over existing QC methods are unclear. Because tile boundaries are case‑specific, it is uncertain how biological variation is distinguished from technical noise. The strangeness metric is not mathematically convincing, and between‑sample comparability is not demonstrated.

2. The manuscript claims successful generalization to an external dataset but provides no information on normalization steps, compensation harmonization, scaling across instruments, or cross‑site preprocessing. Without these details, robustness claims cannot be evaluated.

3. Technical robustness is not assessed as no experiments to examine the impact of instrument variability, compensation perturbations, fluorescence drift, or antibody lot differences—factors known to affect MFC‑based AI systems were performed

4. Removing atypical cases risks eliminating biologically meaningful or rare variants, artificially inflating performance. The consequences of TM filtering on specific lymphoma subtypes are not quantified.

5. Although the system is described as self‑explaining, few concrete examples of clinically interpretable rules are shown, and no comparison with expert gating is provided.

6. Grouping MBL/CLL/PL and handling of HCL within the hierarchy appear model‑driven rather than clinically motivated. Given the changes in the classification; its not possible to reconcile these old labels to the new WHO classification.

7. Weblink does not work. Maybe use a weblink shortener? Code is promised but not available for review, and key algorithmic steps (TM, ALPODS committee) lack sufficient technical detail.

8. Per‑entity performance, calibration, ROC/AUC-PR curves, and confusion matrices should be included to assess real‑world utility given class imbalance.

Minor Issues

The manuscript's organization is dense and repetitive. TM, the diagnostic hierarchy, and background concepts are described multiple times. Some figures require clearer legends and clinical context.

The approach is interesting, however the manuscript requires substantial clarification and additional experiments—particularly around TM, domain shift, and robustness—before the publication.

Reviewer #2: This is an interesting study on using Self-explaining Artificial Intelligence to classify B cell Non-Hodgkin Lymphoma. However, there are a few major issues needing attention.

1. One standing out issue is that the paper is mostly about explaining the advantages of its own FlowXAI system, but lacks systematic and like for like comparisons with other existing machine learning and statistical methods, apart from some ad hoc comparisons in parts of the paper. The proposed FlowXAI system looks well structured, logical and useful AI diagnostic tool, and could be an alternative to the existing ML algorithms if there were evidence from direct like for like comparison with existing mainstream algorithms. But so far, it is mostly talking by by itself and lacks systematic and convincing evidence showing novelty and better performance. Also, the paper is written with a substantial technical details with AI jargons and quite difficult to follow by clinical audience of PLOS Med. The paper may be more suitable for digital health journals elsewhere.

2. Performance, Page 14. It says 'Performance within the confident category showed consistently high MCC values, ranging from 88% to 91% (median 90%), with an approximately Gaussian distribution (magenta frame) (Fig. 3d). The probable category yielded intermediate performance (median MCC 77%), while the challenging category exhibited lower and more variable performance (median MCC 60%). These results indicate that FlowXAI's internal self-assessment is informative with respect to diagnostic reliability'. What other mainstream algorithms can offer for these 3 categories based on the same dataset? The proposed system doesn't seem anything special here.

3. Figure 1 shows the unsupervised clustering results. There are many well-establishing clustering algorithms around and they can do at least similar job. It is not clear what the authors showed here which has never been done by others.

4. External validation, Page 9. It says "To assess robustness under domain shift, FlowXAI was further evaluated using the PUM2 dataset, an independent external benchmark dataset originating from a separate diagnostic center and employing a different antibody panel and tube configuration". But, not sure it's make sense to test on datasets "employing a different antibody panel and tube configuration". Also, the system seemed being trained again in the external dataset? Ultimately, resutls from external validation should be regarded as the final for model/system performance. However, in this paper, most performance results were from the training dataset which is inadequate. Although this is an AI application paper, it would be good to follow the TRIPOD statement for clinical prediction models.

Reviewer #3: This manuscript presents FlowXAI, an artificial intelligence framework combining an unsupervised preprocessing step (Tile Mining, TM) with a rule-based expert committee (ALPODS) to support the diagnosis of chronic B-cell lymphoproliferative disorders from multiparametric flow cytometry data. The objective of developing an interpretable and clinically aligned decision-support system is timely and addresses an important need in hematopathology.

The integration of unsupervised structure analysis, hierarchical decision-making, and explainability represents a clear strength of the work. The involvement of expert clinical teams, together with the use of real-world datasets, further enhances its relevance. In addition, the extensive supplementary material provides valuable insight into the behavior of the system.

That being said, several aspects of the methodology, validation strategy, and presentation would benefit from further clarification and refinement. Addressing the points below would substantially improve the transparency, reproducibility, and clinical interpretability of the study.

Major Concerns

The manuscript emphasizes the interpretability of the ALPODS framework, yet the underlying decision logic remains only partially accessible. Although the supplementary material provides useful information on the architecture of the expert committee, the concrete decision rules that lead to the final classification are not presented in a form that can be readily examined or reproduced . In particular, explicit marker thresholds and clearly defined decision paths are not detailed, which makes it difficult to fully assess both the biological plausibility and the stability of the model. Providing representative decision trees or a concise set of decision rules would considerably strengthen the interpretability claims.

Tile Mining is a central component of the proposed pipeline, but its formulation would benefit from a more explicit and formal description. The "strangeness" metric is introduced conceptually without a precise mathematical definition, and the normalization and aggregation steps across marker-pair tiles are not fully described. Furthermore, the method appears to rely on assumptions regarding the distribution of strangeness values and applies thresholding to define atypical samples. The supplementary material suggests that related distributions may deviate from normality in certain contexts, reflecting underlying data heterogeneity . In this setting, it would be helpful to provide visualizations of the strangeness distribution and to clarify whether the assumed distributional properties are appropriate. A clearer justification of the chosen threshold, supported for instance by sensitivity analyses, would further strengthen this component of the methodology.

The use of TM to identify atypical samples raises an additional conceptual issue, namely the distinction between technical artefacts and biologically atypical but clinically meaningful cases. The supplementary material indicates that a substantial proportion of atypical samples may correspond to technical issues, such as incomplete lysis or staining artefacts, although this assessment remains largely qualitative . As a result, there is a potential risk that clinically relevant variability could be inadvertently excluded from the training process. A more explicit discussion of this distinction, or additional analyses where feasible, would strengthen the interpretation of the results.

Several key parameters, including the number of representative samples, the TM threshold, and aspects of the hierarchical structure, appear to be defined empirically. The use of a reduced training set is an interesting feature of the approach and is supported by results presented in the supplementary material . Nevertheless, additional justification would be valuable to demonstrate that the reported performance is robust across different parameter settings. Even limited sensitivity analyses would help to support this point.

The benchmarking strategy is a positive aspect of the manuscript, notably through the comparison with CITRUS. However, a more explicit positioning of the method relative to a broader set of approaches would help clarify its added value. In addition, the performance of the model appears uneven across diagnostic categories. The contingency tables provided in the supplementary material suggest that classification is most effective for common entities such as CLL-like cases, which are also the most represented in the dataset, whereas performance for less frequent entities appears more variable . This observation is understandable but would benefit from a more explicit discussion, particularly regarding the dependence of performance on class prevalence and the implications for clinical use.

Closely related to this point, the performance of the approach appears to depend on the information content of the antibody panel. The supplementary material indicates that some lymphoma entities are not clearly separable given the available markers . This suggests that the limitations of the model may partly reflect intrinsic biological constraints rather than purely algorithmic factors. A more explicit discussion of the relationship between panel design, biological separability, and model performance would provide important context for interpreting the results and assessing their generalizability.

The validation strategy includes repeated train/test splits and the use of an additional dataset, which are both strengths. The inclusion of a dataset acquired with a different panel is particularly valuable, as it provides a stringent test of robustness across heterogeneous settings. At the same time, differences in panel composition and training strategy make it more difficult to isolate the respective contributions of the model and the experimental configuration. A complementary evaluation using a fixed trained model applied to an independent cohort would further strengthen the assessment of generalizability. In addition, TM appears to be computed prior to data splitting; although this step is unsupervised, recomputing it within each training fold could be considered to further reinforce methodological rigor.

The manuscript reports global performance metrics such as accuracy and MCC, and the supplementary material provides detailed contingency tables stratified by confidence levels, which is a clear strength . Nevertheless, the evaluation could be further enriched by incorporating clinically oriented metrics such as predictive values and by examining the calibration of the confidence scores. Such analyses would enhance the clinical interpretability of the results.

Finally, the positioning of the system within clinical practice would benefit from clarification. While the manuscript sometimes presents FlowXAI as a diagnostic system, the results and supplementary material indicate that certain diagnostic decisions still require additional information, including morphology or molecular data . Framing the system more explicitly as a decision-support or triage tool would align its description with its demonstrated capabilities.

Minor Concerns

The description of the cohorts could be expanded to include more detailed demographic and clinical characteristics.

The statistical methods used for comparisons are not always explicitly specified and would benefit from clarification.

Several minor typographical and language issues are present throughout the manuscript. For example, expressions such as "NC-associated structure" and "self-competence" are unclear or appear to be used in a non-standard way in this context. In addition, capitalization is sometimes inconsistent (for instance, alternating between "Flow Cytometry" and "flow cytometry"). A careful linguistic revision would improve overall clarity and readability. More generally, some sentences—particularly in the Methods section—are syntactically dense or ambiguous, which may hinder comprehension for readers.

The figures are generally informative, and the supplementary material provides valuable visual support. However, several aspects could be improved to enhance readability and interpretability. In particular, some figure legends lack sufficient detail, and the meaning of color scales is not always immediately clear. Axis labels are occasionally ambiguous, and certain figures require substantial prior knowledge to be fully interpreted.

The manuscript would benefit from including more explicit visualizations of the decision logic, such as simplified decision trees or representative rule paths. A schematic representation of the confidence categories described in the text would also improve clarity. In addition, a visualization of the strangeness distribution would be particularly relevant given its central role in the methodology. Finally, some supplementary figures are difficult to read at standard resolution and could benefit from improved graphical quality.

Any attachments provided with reviews can be seen via the following link: [LINK]

--------------------------------------------------------- ---

General editorial requests:

(Note: not all will apply to your paper, but please check each item carefully)

*We ask every co-author listed on the manuscript to fill in a contributing author statement, making sure to declare all competing interests. If any of the co-authors have not filled in the statement, we will remind them to do so when the paper is revised. If all statements are not completed in a timely fashion this could hold up the re-review process. If new competing interests are declared later in the revision process, this may also hold up the submission. Should there be a problem getting one of your co-authors to fill in a statement we will be in contact. Please do not add or remove authors without first discussing this with the handling editor. You can see our competing interests policy here: http://journals.plos.org/plosmedicine/s/competing-interests.

*Please upload any figures associated with your paper as individual TIF or EPS files with 300dpi resolution at resubmission; please read our figure guidelines for more information on our requirements: http://journals.plos.org/plosmedicine/s/figures. While revising your submission, we strongly recommend that you use PLOS's NAAS tool (https://ngplosjournals.pagemajik.ai/artanalysis) to test your figure files. NAAS can convert your figure files to the TIFF file type and meet basic requirements (such as print size, resolution), or provide you with a report on issues that do not meet our requirements and that NAAS cannot fix.

After uploading your figures to PLOS's NAAS tool - https://ngplosjournals.pagemajik.ai/artanalysis, NAAS will process the files provided and display the results in the "Uploaded Files" section of the page as the processing is complete.

If the uploaded figures meet our requirements (or NAAS is able to fix the files to meet our requirements), the figure will be marked as "fixed" above. If NAAS is unable to fix the files, a red "failed" label will appear above.

When NAAS has confirmed that the figure files meet our requirements, please download the file via the download option, and include these NAAS processed figure files when submitting your revised manuscript.

*Please ensure that the paper adheres to the PLOS Data Availability Policy (see http://journals.plos.org/plosmedicine/s/data-availability), which requires that all data underlying the study's findings be provided in a repository or as Supporting Information. For data residing with a third party, authors are required to provide instructions with contact information (web or email address) for obtaining the data. Please note that a study author cannot be the contact person for the data. PLOS journals do not allow statements supported by "data not shown" or "unpublished results." For such statements, authors must provide supporting data or cite public sources that include it.

*We expect all researchers with submissions to PLOS in which author-generated code underpins the findings in the manuscript to make all author-generated code available without restrictions upon publication of the work. In cases where code is central to the manuscript, we may require the code to be made available as a condition of publication. Authors are responsible for ensuring that the code is reusable and well documented. Please make any custom code available, either as part of your data deposition or as a supplementary file. Please add a sentence to your data availability statement regarding any code used in the study, e.g. "The code used in the analysis is available from Github [URL] and archived in Zenodo [DOI link]" Please review our guidelines at https://journals.plos.org/plosmedicine/s/materials-software-and-code-sharing and ensure that your code is shared in a way that follows best practice and facilitates reproducibility and reuse. Because Github depositions can be readily changed or deleted, we encourage you to make a permanent DOI'd copy (e.g. in Zenodo) and provide the URL.

* Please, made the code available for the review (as requested by reviewer #1)

*Please ensure that the study is reported according to the TRIPOD guideline and include the completed TRIPOD (-AI) checklist as Supporting Information. When completing the checklist, please use section and paragraph numbers, rather than page numbers. Please add the following statement, or similar, to the Methods: "This study is reported as per [XXXX] guideline (S1 Checklist)."

FORMATTING - GENERAL

*Abstract: Please structure your abstract using the PLOS Medicine headings (Background, Methods and Findings, Conclusions). Please combine the Methods and Findings sections into one section.

*At this stage, we ask that you include a short, non-technical Author Summary of your research to make findings accessible to a wide audience that includes both scientists and non-scientists. The Author Summary should immediately follow the Abstract in your revised manuscript. This text is subject to editorial change and should be distinct from the scientific abstract. Ideally each sub-heading should contain 2-3 single sentence, concise bullet points containing the most salient points from your study. In the final bullet point of 'What Do These Findings Mean?', please include the main limitations of the study in non-technical language. Please see our author guidelines for more information: https://journals.plos.org/plosmedicine/s/revising-your-manuscript#loc-author-summary.

*Please express the main results with 95% CIs as well as p values. When reporting p values please report as p<0.001 and where higher as the exact p value p=0.002, for example. Throughout, suggest reporting statistical information as follows to improve clarity for the reader "22% (95% CI [13%,28%]; p</=)". Please be sure to define all numerical values at first use.

*Please include page numbers and line numbers in the manuscript file. Use continuous line numbers (do not restart the numbering on each page).

*Please cite the reference numbers in square brackets. Citations should precede punctuation.

FIGURES AND TABLES

*Please provide titles and legends for all figures and tables (including those in Supporting Information files).

*Please define all abbreviations used in each figure/table (including those in Supporting Information files).

*Please consider avoiding the use of red and green in order to make your figure more accessible to those with color blindness.

SUPPLEMENTARY MATERIAL

*Please note that supplementary material will be posted as supplied by the authors. Therefore, please amend it according to the relevant comments outlined here.

*Please cite your Supporting Information as outlined here: https://journals.plos.org/plosmedicine/s/supporting-information

REFERENCES

*PLOS uses the numbered citation (citation-sequence) method and first six authors, et al.

*Please ensure that journal name abbreviations match those found in the National Center for Biotechnology Information (NCBI) databases (http://www.ncbi.nlm.nih.gov/nlmcatalog/journals), and are appropriately formatted and capitalised.

*Where website addresses are cited, please include the complete URL and specify the date of access (e.g. [accessed: 12/06/2023]).

*Please also see https://journals.plos.org/plosmedicine/s/submission-guidelines#loc-references for further details on reference formatting.

STUDY TYPE-SPECIFIC REQUESTS:

MODELLING STUDIES

The following list is derived from Geoffrey P Garnett, Simon Cousens, Timothy B Hallett, Richard Steketee, Neff Walker. Mathematical models in the evaluation of health programmes. (2011) Lancet DOI:10.1016/S0140-6736(10)61505-X:

*If pertinent, please provide a diagram that shows the model structure, including how the natural history of the disease is represented, the process and determinants of disease acquisition, and how the putative intervention could affect the system.

*Please provide a complete list of model parameters, including clear and precise descriptions of the meaning of each parameter, together with the values or ranges for each, with justification or the primary source cited and important caveats about the use of these values noted.

*Please provide a clear statement about how the model was fitted to the data, including goodness-of-fit measure, the numerical algorithm used, which parameter varied, constraints imposed on parameter values, and starting conditions.

*For uncertainty analyses, please state the sources of uncertainties quantified and not quantified [can include parameter, data, and model structure].

*Please provide sensitivity analyses to identify which parameter values are most important in the model. Uncertainty estimates seek to derive a range of credible results on the basis of an exploration of the range of reasonable parameter values. The choice of method should be presented and justified.

*Please discuss the scientific rationale for the choice of model structure and identify points where this choice could influence conclusions drawn. Please also describe the strength of the scientific basis underlying the key model assumptions.

*For studies that develop a prediction model or evaluate its performance, please ensure that the study is reported according to the TRIPOD statement (https://www.equator-network.org/reporting-guidelines/tripod-statement) and include the completed checklist as Supporting Information. Please add the following statement, or similar, to the Methods: "This study is reported as per the Transparent Reporting of a Multivariable Prediction Model for Individual Prognosis Or Diagnosis (TRIPOD) statement (S1 Checklist)." For studies using machine learning, please use the TRIPOD-AI checklist. When completing the checklist, please use section and paragraph numbers, rather than page numbers.

# Author comment: Author response to Decision Letter 2

**DOI:** 10.1371/journal.pmed.1004889.r003

22 Apr 2026

Attachment Submitted filename: response2reviewers_V9.docx

# Peer review: Decision Letter 2

**DOI:** 10.1371/journal.pmed.1004889.r004

**Authors:** Katarzyna Starowicz

26 May 2026

Dear Dr. Thrun,

Thank you very much for re-submitting your manuscript "Self-explaining Artificial Intelligence for the Classification of B cell Non-Hodgkin Lymphoma" (PMEDICINE-D-25-04525R2) for review by PLOS Medicine.

I have discussed the paper with my colleagues and the academic editor and it was also seen again by three reviewers. There are remaining editorial and reviewers concerns that need to be address in full.

The remaining issues that need to be addressed are listed at the end of this email. As previously, please provide point-by-point response to ALL editorial and reviewers requests to avoid delay in processing your manuscript. Any accompanying reviewer attachments can be seen via the link below. Please take these into account before resubmitting your manuscript:

[LINK]

***Please note while forming your response, if your article is accepted, you may have the opportunity to make the peer review history publicly available. The record will include editor decision letters (with reviews) and your responses to reviewer comments. If eligible, we will contact you to opt in or out.***

In revising the manuscript for further consideration here, please ensure you address the specific points made by each reviewer and the editors. In your rebuttal letter you should indicate your response to the reviewers' and editors' comments and the changes you have made in the manuscript. Please submit a clean version of the paper as the main article file. A version with changes marked must also be uploaded as a marked up manuscript file.

Please also check the guidelines for revised papers at http://journals.plos.org/plosmedicine/s/revising-your-manuscript for any that apply to your paper. If you haven't already, we ask that you provide a short, non-technical Author Summary of your research to make findings accessible to a wide audience that includes both scientists and non-scientists. The Author Summary should immediately follow the Abstract in your revised manuscript. This text is subject to editorial change and should be distinct from the scientific abstract.

In addition to these revisions, you may need to complete some formatting changes, which you will receive in a follow up email. A member of our team will be in touch with a set of requests shortly. If you do not receive a separate email within a few days, please assume that checks have been completed, and no additional changes are required.

We expect to receive your revised manuscript within 1 week. Please email us (plosmedicine@plos.org) if you have any questions or concerns.

We ask every co-author listed on the manuscript to fill in a contributing author statement. If any of the co-authors have not filled in the statement, we will remind them to do so when the paper is revised. If all statements are not completed in a timely fashion this could hold up the re-review process. Should there be a problem getting one of your co-authors to fill in a statement we will be in contact. YOU MUST NOT ADD OR REMOVE AUTHORS UNLESS YOU HAVE ALERTED THE EDITOR HANDLING THE MANUSCRIPT TO THE CHANGE AND THEY SPECIFICALLY HAVE AGREED TO IT.

Please ensure that the paper adheres to the PLOS Data Availability Policy (see http://journals.plos.org/plosmedicine/s/data-availability), which requires that all data underlying the study's findings be provided in a repository or as Supporting Information. For data residing with a third party, authors are required to provide instructions with contact information for obtaining the data. PLOS journals do not allow statements supported by "data not shown" or "unpublished results." For such statements, authors must provide supporting data or cite public sources that include it.

To enhance the reproducibility of your results, we recommend that you deposit your laboratory protocols in protocols.io, where a protocol can be assigned its own identifier (DOI) such that it can be cited independently in the future. Additionally, PLOS ONE offers an option to publish peer-reviewed clinical study protocols. Read more information on sharing protocols at https://plos.org/protocols?utm_medium=editorial-email&utm_source=authorletters&utm_campaign=protocols

Please review your reference list to ensure that it is complete and correct. If you have cited papers that have been retracted, please include the rationale for doing so in the manuscript text, or remove these references and replace them with relevant current references. Any changes to the reference list should be mentioned in the rebuttal letter that accompanies your revised manuscript.

Please note, when your manuscript is accepted, an uncorrected proof of your manuscript will be published online ahead of the final version, unless you've already opted out via the online submission form. If, for any reason, you do not want an earlier version of your manuscript published online or are unsure if you have already indicated as such, please let the journal staff know immediately at plosmedicine@plos.org.

If you have any questions in the meantime, please contact me or the journal staff on plosmedicine@plos.org.

We look forward to receiving the revised manuscript by Jun 05 2026 11:59PM.

Sincerely,

Katarzyna Starowicz, PhD

Senior Editor

PLOS Medicine

plosmedicine.org

------------------------------------------------------------

Requests from Editors:

* At this stage, we ask that you include a short, non-technical Author Summary of your research to make findings accessible to a wide audience that includes both scientists and non-scientists. The Author Summary should immediately follow the Abstract in your revised manuscript. This text is subject to editorial change and should be distinct from the scientific abstract. Ideally each sub-heading should contain 2-3 single sentence, concise bullet points containing the most salient points from your study.

* In the author summary, please revise formatting and ensure you use bullet points.

* In the final bullet point of ‘What Do These Findings Mean?’ Please include the main limitations of the study in non-technical language.

Please see our author guidelines for more information: https://journals.plos.org/plosmedicine/s/revising-your-manuscript#loc-author-summary.

* Please confirm that your title complies with PLOS Medicine's style. Your title must be nondeclarative and not a question. It should begin with main concept if possible. "Effect of" should be used only if causality can be inferred, i.e., for an RCT. Please place the study design ("A randomized controlled trial," "A retrospective study," "A modelling study," etc.) in the subtitle (ie, after a colon).

* Please confirm that your abstract complies with our requirements, including format (three sections: Background, Methods and Findings, and Conclusions) and providing all the information relevant to this study type https://journals.plos.org/plosmedicine/s/submission-guidelines#loc-abstract

* In the last sentence of the Abstract Methods and Findings section, please describe the main limitation(s) of the study's methodology.

* In the abstract, please include the important dependent variables that are adjusted for in the analyses.

* Please ensure that the Introduction ends with a clear description of the study question or hypothesis.

* Please ensure that all abbreviations are defined at first use throughout the text.

* Please confirm that all numbers presented in the abstract are present and identical to numbers presented in the main manuscript text.

* Please disclose any use of AI tools and technologies to the study or article content in the Methods, including the name(s) of any tool(s) used and a description of their use. Please see our guidelines for more information: https://journals.plos.org/plosmedicine/s/ethical-publishing-practice#loc-artificial-intelligence-tools-and-technologies.

* Please review your text for claims of novelty or primacy (e.g. 'for the first time') and remove this language. In addition, please check that any use of statistical terms (such as trend or significant) are supported by the data, and if not please remove them.

* Statistical reporting: Please revise throughout the manuscript, including tables and figures.

- Please report statistical information as follows to improve clarity for the reader ""22% (95% CI [13,28]; p</=)"".

- Please separate upper and lower bounds with commas instead of hyphens as the latter can be confused with reporting of negative values.

- Please repeat statistical definitions (HR, CI etc.) for each set of parentheses."

* The funding statement should include: specific grant numbers, initials of authors who received each award, URLs to sponsors’ websites. Also, please state whether any sponsors or funders (other than the named authors) played any role in study design, data collection and analysis, the decision to publish, or preparation of the manuscript. If they had no role in the research, include this sentence: “The funders had no role in study design, data collection and analysis, decision to publish, or preparation of the manuscript.”

* All authors must declare their relevant competing interests per the PLOS policy, which can be seen here: https://journals.plos.org/plosmedicine/s/competing-interests For authors with ties to industry, please indicate whether any of the interests has a financial stake in the results of the current study.

*Please amend your competing interests statement. Funding information should go to the relevant section and include URLs.

"* PLOS Medicine requires that the de-identified data underlying the specific results in a published article be made available, without restrictions on access, in a public repository or as Supporting Information at the time of article publication, provided it is legal and ethical to do so. Please see the policy at

http://journals.plos.org/plosmedicine/s/data-availability

and FAQs at

http://journals.plos.org/plosmedicine/s/data-availability#loc-faqs-for-data-policy "

* PLOS defines the “minimal data set” to consist of the data set used to reach the conclusions drawn in the manuscript with related metadata and methods, and any additional data required to replicate the reported study findings in their entirety. Authors do not need to submit their entire data set, or the raw data collected during an investigation. Please submit the following data:

The values behind the means, standard deviations and other measures reported;

The values used to build graphs;

The points extracted from images for analysis."

* The Data Availability Statement (DAS) requires revision. For each data source used in your study:

a) If the data are freely or publicly available, note this and state the location of the data: within the paper, in Supporting Information files, or in a public repository (include the DOI or accession number).

b) If the data are owned by a third party but freely available upon request, please note this and state the owner of the data set and contact information for data requests (web or email address). Note that a study author cannot be the contact person for the data.

c) If the data are not freely available, please describe briefly the ethical, legal, or contractual restriction that prevents you from sharing it. Please also include an appropriate contact (web or email address) for inquiries (again, this cannot be a study author).

* Please revise for use of patient-centered language. Please note that patient-centered language is constructed with the use of post-modified nouns (e.g. 'patients with psoriasis’ (or similar) instead of ‘psoriasis patients’) putting the person first in the sentence structure.

* Please specify whether informed consent was written or oral. Please ensure that the research complies with the PLOS policy in full: https://journals.plos.org/plosmedicine/s/human-subjects-research#loc-patient-privacy-and-informed-consent-for-publication

* Figures cannot be reproduced from other sources that are not CC-BY. Please provide a different figure.

* Please provide titles and legends for all figures and tables (including those in Supporting Information files). Please define all acronyms used in each figure or table in its corresponding legend.

* Please ensure that where relevant figures include 95% CIs.

* When a p value is given, please specify the statistical test used to determine it in the legend.

* Please consider avoiding the use of red and green in order to make your figure more accessible

* Please consider if moving some of the figures shared in the supplementary information into the main text would aid the reader. I think moving workflow/model structure to main manuscript body will be beneficial for readers.

* Please include an acknowledgment of study participants.

* Thank you for providing TRIPOD statement document. Could you please amend it's name to S1 Checklist and attach it as supporting information. Please revise your manuscript to include following statement: "This study is reported as per TRIPOD-AI guideline (S1 Checklist)."

* Please revise your TRIPOD statement and as instructed previously when completing the checklist, please use section and paragraph numbers, rather than page numbers.

----------------------------------

Comments from Reviewers:

Reviewer #1: The edits and changes appear to resolve the concerns brought up in the prior review.

Reviewer #2: Many thanks authors for their great effort to improve the manuscript. The authors have addressed my concerns very well in a professional way. I am satisfied with the response and revision. No further issues needing attention.

Reviewer #3: I thank the authors for their careful revision of the manuscript and for the detailed responses provided. The manuscript has improved compared to the initial submission, particularly with respect to clarity of presentation and the documentation of key methodological components.

The description of the Tile Mining (TM) procedure is now more explicit, with the addition of more structured explanations in the main text and in the supplementary material. The clarification of its intended role as a sample-level structural filtering step is helpful. The authors have also expanded the description of the ALPODS framework and provided additional material regarding the organization of the expert committee, which contributes to a better understanding of the overall approach.

That being said, several points would still benefit from further clarification or more explicit discussion.

The interpretability of the ALPODS framework, although improved, remains somewhat indirect. While additional material is now available, the decision logic is not yet presented in a concise and directly accessible form within the main manuscript. In particular, the exact nature of the individual "experts" remains somewhat implicit. It is not entirely clear to what extent these components correspond to explicit rule-based systems, learned classifiers, or hybrid decision modules. Clarifying this point, and providing representative decision paths or simplified rule sets, would facilitate a more direct evaluation of the interpretability claims.

Regarding the TM procedure, the formalization is clearer than in the initial version. However, the thresholding strategy remains primarily empirically motivated. While the authors provide a rationale for the use of a Gaussian-based rule, the sensitivity of the results to this choice is not explored in detail. A brief discussion of this aspect would help to better contextualize the robustness of the method. More generally, the TM step appears to capture distributional structures of cell populations across marker pairs, which can be seen as an implicit representation of population-level organization. It would be helpful to clarify how stable these representations are across datasets and parameter settings.

The clarification that TM does not distinguish between technical artefacts and biologically atypical samples is useful. At the same time, this highlights a limitation that may affect the representativeness of the training data, particularly for rare or heterogeneous entities. This point would benefit from being more explicitly acknowledged in the discussion.

The validation framework has been clarified, and the distinction between cross-site benchmarking and strict external validation is now appropriately stated. This is a positive point. However, this also indicates that a full assessment of model generalizability on an independent cohort with identical predictors has not yet been performed, which could be more clearly discussed as a limitation.

The additional analyses provided by the authors confirm that performance is not uniform across diagnostic categories. As reported in the revised manuscript, classification appears strongest for entities such as normal controls, CLL-like cases, and HCL, while remaining more limited for others such as MCL, FL, and LPL. This heterogeneity is clinically relevant and would merit a more prominent discussion in the main text, as it directly affects the potential scope of application of the method.

More generally, the performance of the approach appears to depend on the information content of the antibody panel. The supplementary material suggests that some entities are not clearly separable given the available markers. This indicates that part of the limitation may be related to intrinsic biological separability rather than algorithmic performance alone. A clearer discussion of this dependency would provide useful context for interpreting the results.

The manuscript refers to an online platform for interactive exploration of the model. Access to this resource requires user registration, which prevented its evaluation in the context of this review. While such a tool may be useful, it would be important to ensure that all essential elements required for understanding and assessing the method remain fully accessible within the manuscript and supplementary material.

Finally, while the manuscript reports appropriate global performance metrics, the inclusion of additional clinically oriented metrics or calibration analyses could further improve the interpretability of the results.

The manuscript has improved following revision and now presents a coherent and potentially useful approach. The remaining points mainly concern clarification, accessibility, and positioning of the results rather than fundamental methodological issues. Addressing these aspects would further strengthen the clarity and clinical relevance of the work.

Any attachments provided with reviews can be seen via the following link:

[LINK]

# Author comment: Author response to Decision Letter 3

**DOI:** 10.1371/journal.pmed.1004889.r005

5 Jun 2026

Attachment Submitted filename: response_to_reviewer3_second_round_V4.docx

# Peer review: Decision Letter 3

**DOI:** 10.1371/journal.pmed.1004889.r006

**Authors:** Katarzyna Starowicz

18 Jun 2026

Dear Dr Thrun,

On behalf of my colleagues and the Academic Editor, Nic Gabriel Reitsam, I am pleased to inform you that we have agreed to publish your manuscript "Self-explaining artificial intelligence for the classification of B cell non-Hodgkin lymphoma:

A trustworthiness-aware diagnostic decision support development and external validation study using flow cytometry." (PMEDICINE-D-25-04525R3) in PLOS Medicine.

Before your manuscript can be formally accepted you will need to complete some formatting changes, which you will receive in a follow up email. Please be aware that it may take several days for you to receive this email; during this time no action is required by you. Once you have received these formatting requests, please note that your manuscript will not be scheduled for publication until you have made the required changes.

In the meantime, please log into Editorial Manager at http://www.editorialmanager.com/pmedicine/, click the "Update My Information" link at the top of the page, and update your user information to ensure an efficient production process.

PRESS

We frequently collaborate with press offices. If your institution or institutions have a press office, please notify them about your upcoming paper at this point, to enable them to help maximise its impact. If the press office is planning to promote your findings, we would be grateful if they could coordinate with medicinepress@plos.org. If you have not yet opted out of the early version process, we ask that you notify us immediately of any press plans so that we may do so on your behalf.

We also ask that you take this opportunity to read our Embargo Policy regarding the discussion, promotion and media coverage of work that is yet to be published by PLOS. As your manuscript is not yet published, it is bound by the conditions of our Embargo Policy. Please be aware that this policy is in place both to ensure that any press coverage of your article is fully substantiated and to provide a direct link between such coverage and the published work. For full details of our Embargo Policy, please visit http://www.plos.org/about/media-inquiries/embargo-policy/.

To enhance the reproducibility of your results, we recommend that you deposit your laboratory protocols in protocols.io, where a protocol can be assigned its own identifier (DOI) such that it can be cited independently in the future. Additionally, PLOS ONE offers an option to publish peer-reviewed clinical study protocols. Read more information on sharing protocols at https://plos.org/protocols?utm_medium=editorial-email&utm_source=authorletters&utm_campaign=protocols

Thank you again for submitting to PLOS Medicine. We look forward to publishing your paper.

Sincerely,

Katarzyna Starowicz, PhD

Associate Editor

PLOS Medicine
